# A multi-dimensional, time-lapse, high content screening platform applied to schistosomiasis drug discovery

**DOI:** 10.1038/s42003-020-01402-5

**Published:** 2020-12-21

**Authors:** Steven Chen, Brian M. Suzuki, Jakob Dohrmann, Rahul Singh, Michelle R. Arkin, Conor R. Caffrey

**Affiliations:** 1 Department of Pharmaceutical Chemistry and Small Molecule Discovery Center, University of California, San Francisco, CA 94143 USA; 2 Center for Discovery and Innovation in Parasitic Diseases, Department of Pathology, University of California, San Francisco, CA 94158 USA; 3 Center for Discovery and Innovation in Parasitic Diseases, Skaggs School of Pharmacy and Pharmaceutical Sciences, University of California, San Diego, La Jolla, CA 92093 USA; 4 Department of Computer Science, San Francisco State University, San Francisco, CA 94132 USA

**Keywords:** Time-lapse imaging, Parasite biology, Screening

## Abstract

Approximately 10% of the world’s population is at risk of schistosomiasis, a disease of poverty caused by the *Schistosoma* parasite. To facilitate drug discovery for this complex flatworm, we developed an automated high-content screen to quantify the multidimensional responses of *Schistosoma mansoni* post-infective larvae (somules) to chemical insult. We describe an integrated platform to process worms at scale, collect time-lapsed, bright-field images, segment highly variable and touching worms, and then store, visualize, and query dynamic phenotypes. To demonstrate the methodology, we treated somules with seven drugs that generated diverse responses and evaluated 45 static and kinetic response descriptors relative to concentration and time. For compound screening, we used the Mahalanobis distance to compare multidimensional phenotypic effects induced by 1323 approved drugs. Overall, we characterize both known anti-schistosomals and identify new bioactives. Apart from facilitating drug discovery, the multidimensional quantification provided by this platform will allow mapping of chemistry to phenotype.

## Abstract (web summary)

Steven Chen et al. develop an automated, time-lapsed, high-content screen to quantify the responses of *Schistosoma mansoni* larvae to chemical insult. They apply their method to evaluate 45 static and kinetic response endpoints for seven drugs and screen 1323 approved drugs. Their work identifies anti-schistosomal compounds and underscores the value of quantifying motion in phenotypic drug discovery.

## Introduction

The *Schistosoma* blood fluke (helminth) causes schistosomiasis, a neglected tropical disease^1–3^ that infects over 200 million people and puts more than 700 million people at risk of infection in 78 countries^4,5^ (https://www.who.int/en/news-room/fact-sheets/detail/schistosomiasis). Parasite eggs cause chronic inflammatory and fibrotic responses that impair visceral and/or urogenital organ function; co-morbidities include increased risks for bladder cancer and HIV^6,7^. Praziquantel (PZQ) is the only available drug for schistosomiasis. Although reasonably active against mature schistosomes, PZQ displays little to no efficacy against developing parasites^8,9^. Also, increased utilization of PZQ raises concerns that drug resistance will emerge. Thus, new drugs are needed^10^.

Anthelmintic drug discovery has traditionally relied on phenotypic screens using parasites in culture or in small animal models^11^. Primary screening of cultured schistosomes has often used post-infective larvae (called schistosomula or somules) that can be obtained in their thousands to tens of thousands from vector snails for relatively little effort and cost, in contrast to adult worms that can only be harvested in low numbers (hundreds) from small mammals. Single-metric assays, in which somules are scored as alive or dead, have been reported. (e.g., see ref. ^12^ for review). However, single-metric approaches have a number of drawbacks; in some cases, even the clinically used drugs do not score as active in these assays^12,13^. High-content imaging of live somules offers the potential to visualize complex and non-lethal (but potentially therapeutically relevant) phenotypic responses to drug treatment.

As part of our research program to develop methods for anti-schistosomal drug discovery^14–16^, we report a fully integrated, automated and multiparametric image-analysis platform for high-throughput phenotyping of living parasites. Starting with a set of seven drugs known to induce changes in shape and motion^14,17^, we describe a set of protocols to quantify those changes as a function of time and concentration. We then demonstrate the utility of the method for high-throughput screening using a set of 1323 approved drugs. Our approach offers key advances in method integration, including several of general utility to the drug screening/imaging community as follows: (a) automated liquid handling of 100 µm-sized organisms; (b) manipulation of the focal plane to facilitate identification of low-contrast, variable, and touching objects; (c) time-lapsed tracking to define frequencies and rates of motion; (d) a public system for storage, visualization and querying of the complex phenotypic data; and (e) use of a statistical metric (the Mahalanobis distance, *d*_M_) to compare multi-dimensional phenotypes for high-throughput screening.

## Results

### High-throughput sample handling for *Schistosoma mansoni* somules

**Fig. 1:**
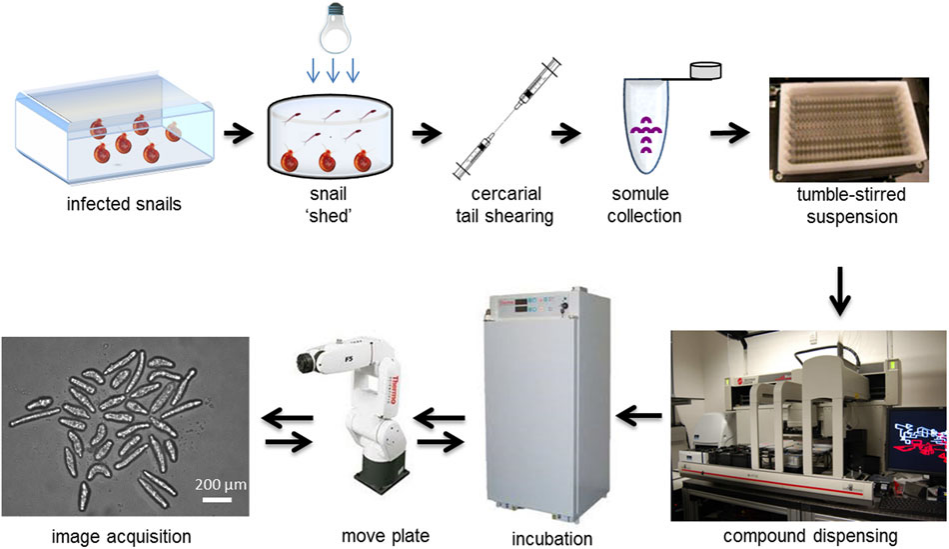
Assay workflow. Infected *B. glabrata* vector snails were stimulated under light to release (“shed”) 10^4^–10^5^ *S. mansoni* infective larvae (cercariae) per week. Cercariae were mechanically converted to post-infective larvae (schistosomula or somules) using a 20-gauge double-headed syringe needle. After washing to remove cercarial tails, somules were suspended in a paddle-stirred reservoir. Somules were then dispensed into 96-well u-bottom clear plates at 40 units/well/200 µL Basch medium. Compounds were added using a 96-channel pin tool. Plates were maintained at 5% CO_2_ and 37 °C. At specified time points, a six-axis robotic arm transferred the plates to the high-content imager.

We selected somules for primary assays as we can obtain 10^4^–10^5^ somules/week from freshwater vector snails. As somules (~300 × 150 µm) rapidly settle out of solution, we used a magnetic tumble stirrer containing eight stirring paddles to maintain worms in suspension (Fig. 1) and allow their transfer using 96- or 384- channel pipets from a reservoir containing 200 mL of media and 40,000 somules. Plate geometry and number of somules/well were optimized for accurate imaging and counting (Supplementary Fig. 1a). In particular, u-bottom 96-well plates concentrated the parasite as a monolayer into one central field of view, thus facilitating automated imaging. Forty somules per well allowed us to maximize the number of worms being tested without reducing our ability to count them due to overlapping (Supplementary Fig. 1a). Routine robotic protocols then dispensed compound and shuttled plates between an incubator (Cytomat 2C) and a high-content imager (GE IN Cell Analyzer 2000) for data collection.

### Imaging schistosomes by automated, bright-field image analysis

Bright-field, time-lapsed images were generated for control and drug-treated somules using a ×10 objective. Every 24 h for 3 days, images were collected at 1.66 Hz (the maximum frame rate for the IN Cell Analyzer 2000) to generate 20 s video recordings. We employed bright-field imaging as it is mechanism-agnostic, non-invasive, and fast, and because the schistosome is not yet routinely amenable to the transgenic incorporation of fluorescent proteins. However, in bright field, somules do not present a high-contrast edge relative to the background, thus limiting object segmentation (detection of the object’s outline). We, therefore, lowered the focal plane 40 µm below the bottom of the well, to artificially generate a dark edge that facilitated segmentation without a significant loss of interior density features (texture; Supplementary Fig. 1b).

**Fig. 2:**
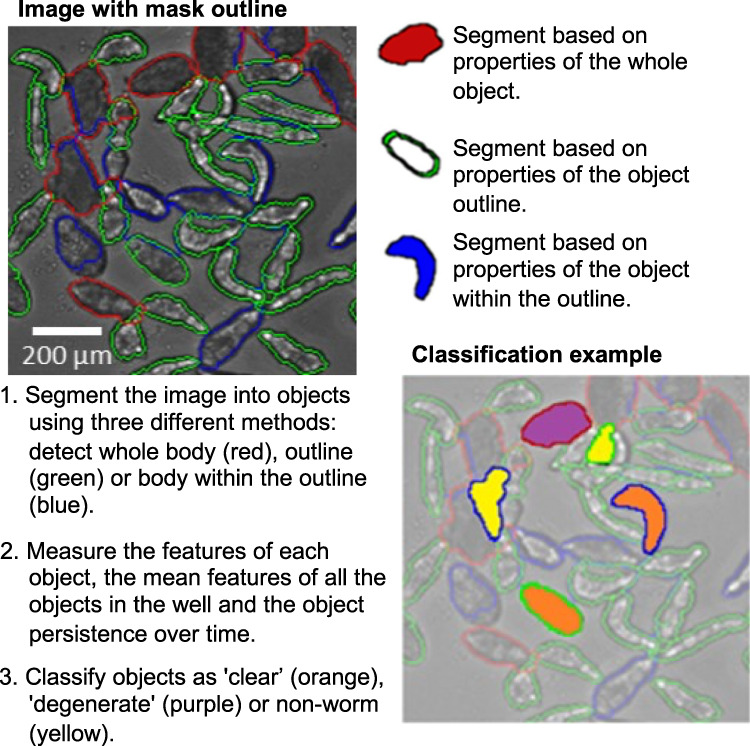
Summary of segmentation and data post-process workflow. Details provided in Supplementary Information. Acquired images (30 time-lapse images per well) were loaded into the IN Cell Developer software and segmented. “Clear” worms, “opaque” worms, and non-worm objects were classified based on features computed on a subset of the data containing 20,000 worm and non-worm objects.

We observed two strong distributions of somules during our studies. Initially, parasites had a translucent body with a discernable outline. However, under the influence of toxic compounds, worms could become progressively opaque, such that the worm outline was indistinguishable from the interior of the worm. This opacity was associated with degenerating/dying parasites, with the transition from “clear” to “opaque” being irreversible. To accurately identify both classes of worms, we segmented the somules using three customized protocols that were optimized to detect somules independently by considering (a) only the worm outline, (b) the worm’s interior, including the inner edge, or (c) only the worm’s interior, excluding the edge (Fig. 2). The most time-lapse-persistent segmented area obtained from these protocols was selected as a true somule. Each somule was then described using 15 features, including those that define size, shape, texture, and color (for terminology, see Supplementary Table 1).

We classified the segmented objects into groups as follows: “clear” (translucent) worms, “opaque” (degenerate/dying) worms, and “non-worm” objects. Compared to the clear worms, degenerate worms had a lower mean intensity level (1822 ± 49 vs. 2143 ± 42 levels), a lower SD of levels in the mask (227 ± 50 vs. 556 ± 49 levels), a larger area (6421 ± 599 vs. 3962 ± 447 µm^2^), and a larger form factor (0.66 ± 0.05 vs. 0.52 ± 0.06). The percentage of somules that were classified as degenerate yielded a “degeneracy” score.

Our image-segmentation protocols identified the somules with a precision of 88% and a recall of 95% for 58,456 worms in the seven-drug-set experiment described below. These results were confirmed by visual inspection (Supplementary Fig. 1C). Using the plating density of 40 worms/well, we observed very few overlapping worms (<0.47%; Supplementary Fig. 1D). The precision and recall values, without the need to remove touching worms, represent a significant improvement over the state-of-the-art^18^. Data collection, segmentation, and classification protocols are described in the Supplementary Information.

Once worms were classified as clear or degenerate, the 15 calculated features were evaluated across three modes—static, rate, and frequency. In the static mode, we considered feature measurements in each frame independently, e.g., worm length. Time-dependent changes in these features were measured using rate and frequency. Rate measured the magnitude of a change in a feature, such as worm length per unit time. Motion could also be characterized by how often the time-dependent measurement changes sign or direction, e.g., the worm becomes longer, then shorter. To capture this aspect, we defined the frequency of a feature to be the number of times the sign of the difference in consecutive feature values changed (i.e., change in sign/unit time). Due to slight offsets in the camera between frames, we set a motion threshold based on a non-moving reference (worms paralyzed by metrifonate). As somules showed little translational movement in the u-bottom wells, we did not record their displacement. When combined, the static, rate, and frequency modes for each of the 15 features yielded 45 measurements (which we define as descriptors) for each somule (see next section).

Two statistical approaches were used to evaluate the significance of changes in worm phenotypes in response to drug treatment. First, the mean and SD for each descriptor for all somules within a well were computed and normalized to determine the Glass Effect Size (ES)^19,20^:

1 $${\mathrm{{ES}}} = \frac{{\bar x - \mu }}{\sigma }$$

where $\bar x$ is the mean descriptor due to drug exposure, *μ* is the dimethylsulfoxide (DMSO) mean, and *σ* is the SD of the descriptor for parasites in DMSO. Being dimensionless, ES is useful in comparing effects across different features. In addition to evaluating individual descriptors, we compared parasites in this descriptor space using the Mahalanobis distance (*d*_M_)^21^, which measures the multi-dimensional scale invariant distance between a test well and a standard condition, e.g., DMSO-treated somules. It is calculated by $d_{\mathrm{{M}}} = \sqrt {\left( {X - Y} \right)^T \ast S^{ - 1} \ast \left( {X - Y} \right)}$ where *X* is the test well vector, *Y* the DMSO vector, *S* the covariance matrix, and *T* indicates that the vector should be transposed. The metrics, *d*_M_ and ES, are similar in that both measure a distance from the DMSO reference. However, ES measures the distance for just one feature, whereas *d*_M_ measures the distance for a group of variables. Moreover, *d*_M_ is not dependent on the measurement unit and can identify test wells that have one large difference or multiple small differences compared to DMSO-treated controls.

### SchistoView: query-visualization of phenotypic screening data

**Fig. 3:**
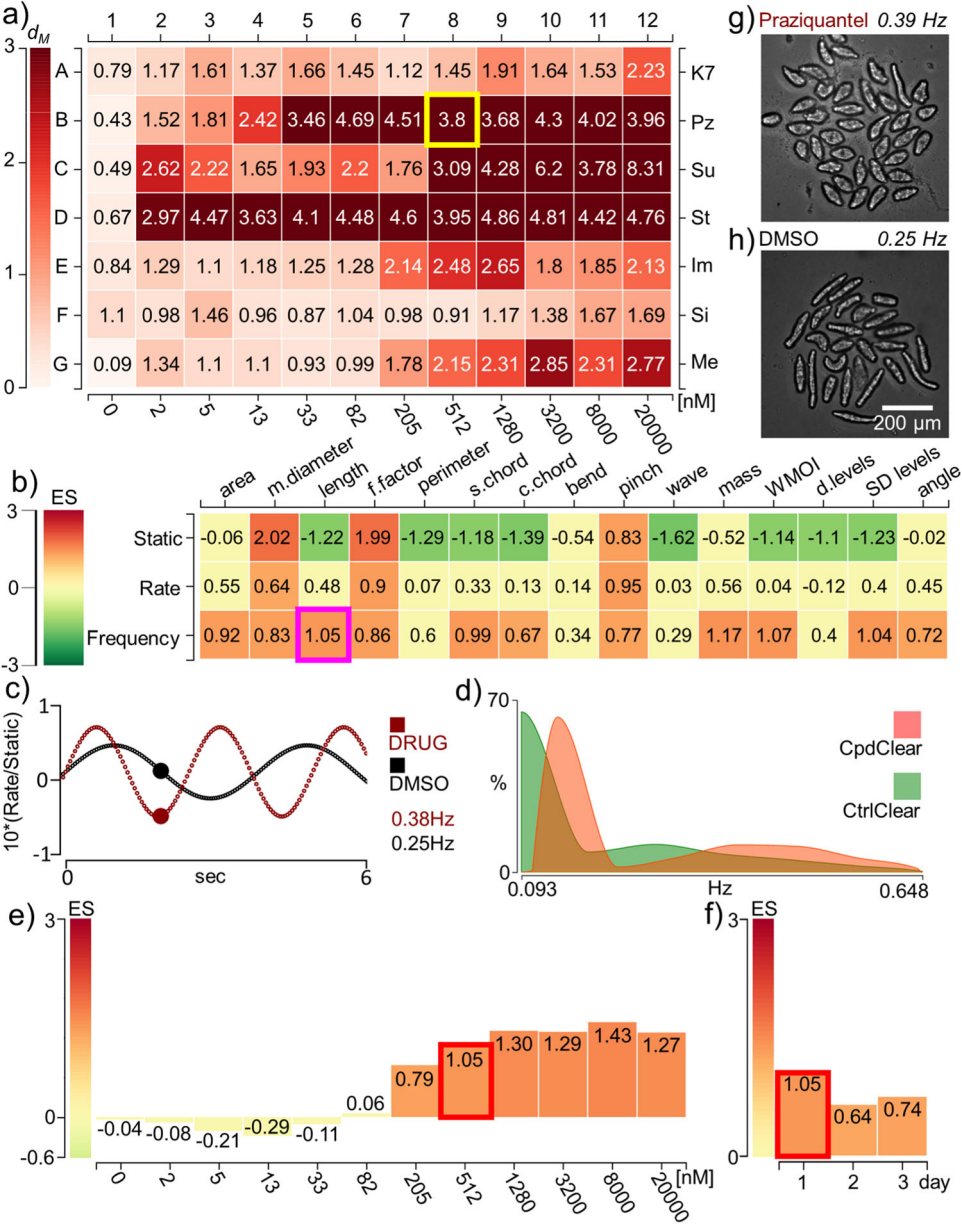
The SchistoView graphical user interface (http://haddock9.sfsu.edu/schistoview/home). Selected data at 2 h illustrate the hierarchical approach to visualization. **a** Heat map of Mahalanobis distances (*d*_M_) for seven test drugs over an 11-point, 2.5-fold dilution series (2 nM–20 μM). The test drugs are K11777 (K7), PZQ (PZ), sunitinib (Su), staurosporine (St), imipramine (Im), simvastatin (Si), and metrifonate (Me). DMSO controls are in column 1 and the average *d*_M_ for each DMSO control is shown. A *d*_M_ of 1.61 is 3 SD from the DMSO control. Clicking on well B8 of the heat map (512 nM PZQ—the yellow square) populates **b**–**g** in the GUI. **b** Heat map of the effect sizes (ES) for each of 15 features (columns) plotted vs. static, rate, or frequency modes (rows); a “descriptor” is the combination of a given feature and mode. Clicking on a descriptor, e.g., frequency of change-in-length (the magenta square) populates **c**–**f**. **c** Calculated waveforms defined by the range of length (amplitude) and the frequency of length contraction. DMSO control worms (black line) are slower moving than those treated with 512 nM PZQ at 2 h (red line). **d** Histogram displaying the distribution of frequency-of-length contraction for DMSO controls (green) and PZQ-treated worms (orange). **e** Dosage bar graph depicting the ES for the frequency-of-length contraction after 2 h with 11 PZQ concentrations. **f** Time bar graph depicting the ES for the frequency-of-length contraction after PZQ (512 nM) treatment across the 3 days indicated. **g** First image from time-lapsed movie of well B8 highlighted in **a**; in the live SchistoView, the 30-frame movie is shown. **h** First image from time-lapsed movie of a DMSO-treated well; in the live SchistoView, the 30-frame movie is shown.

We developed SchistoView (Fig. 3, http://haddock9.sfsu.edu/schistoview/home), which comprises a graphical user interface (GUI) supported by a MySQL database. SchistoView allows users to visualize and query concentration- and time-dependent somule response data from computed statistics for a given well to features for individual somules. Figure 3 shows frames from Schistoview and describes the features of the GUI. Further details are provided in the Supplementary Information.

### Exploring the parasite’s multivariate responses using known anti-schistosomal compounds

We tested the time-lapsed imaging platform with seven compounds that induced diverse changes in the parasite^14,17^. Somules were exposed to an 11-point, 2.5-fold dilution series (from 2 nM–20 μM) of compounds in quadruplicate and images were captured after 2, 24, and 48 h. Raw images, collected after 24 h of the first frame in each well, are shown in Supplementary Fig. 2. Images were segmented and data extracted as described above. The results highlight important features of the imaging methodology, the depth of analysis offered and the underlying biology of the schistosome parasite.

**Fig. 4:**
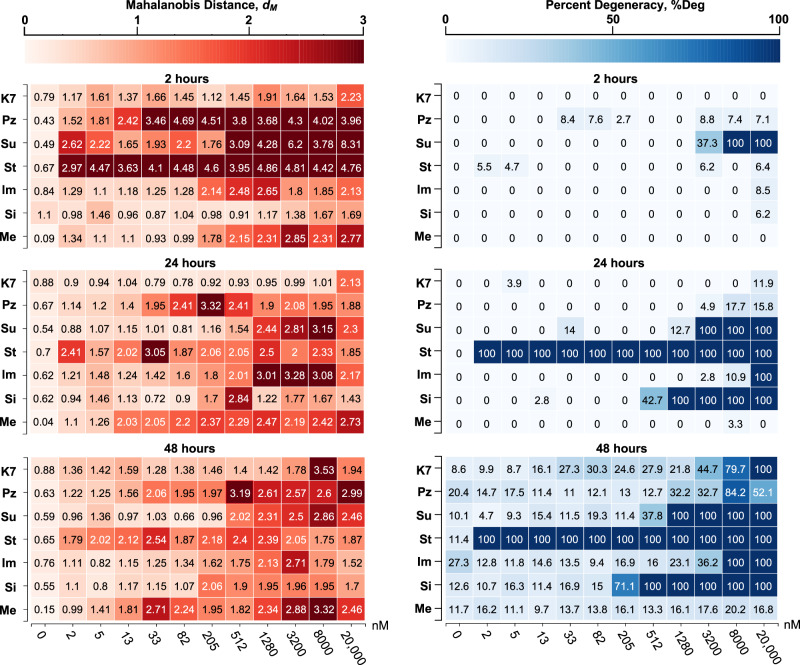
Heat map displaying *d*_M_ and degeneracy for seven test drugs arrayed over a 2.5-fold dilution series from 20 μM to 2 nM. *d*_M_ values were calculated relative to DMSO controls. *d*_M_ values of 1.61, 1.36, and 1.28 are 3 SD from the corresponding controls at 2, 24, and 48 h, respectively. Results presented are aggregated from four wells per treatment group (40 somules/well). For most compounds, phenotypes appeared quickly (within 2 or 24 h), relative to the appearance of degeneracy (pronounced at 48 h). Notably, the two approved anti-schistosomal drugs, PZQ and metrifonate, do not induce significant degeneracy under these conditions. It is also noteworthy that *d*_M_ values can be smaller at later time points. Values for *d*_M_ were calculated using the eight DMSO control wells collected closest in time (±20 min) to the target well. Due to well averaging, the DMSO control well *d*_M_ value shown in the figure is >0 (*d*_M_ cannot be negative). This small, positive value allows us to define “activity” as *d*_M_ > 3 SD from the mean value for the DMSO control wells. Test drug abbreviations: K11777 (K7), PZQ (PZ), sunitinib (Su), staurosporine (St), imipramine (Im), simvastatin (Si), and metrifonate (Me).

In Fig. 4 and Supplementary Fig. 3, the time- and concentration-dependent effects of drugs on worm behavior are visualized using heat maps extracted from SchistoView. It is noteworthy that the *d*_M_ values shown do not necessarily smoothly change with increasing concentration of a drug. This is due to individual features showing maximum changes at different concentrations. For some compounds (e.g., sunitinib, staurosporine and PZQ), it is also noteworthy that at later time points *d*_M_ values can be smaller yet still significant. The biological factors responsible may include compound metabolism and baseline physical changes as the schistosome adapts to its environment.

Using *d*_M_ as a summary of overall static and kinetic phenotypic changes, concentration-dependent effects of drug exposure were already significant by the first time point (2 h) for five of the seven drugs (i.e., *d*_M_ > 1.61, which is 3 SD from the *d*_M_ of the DMSO controls)^22^. These drugs included PZQ, the kinase inhibitors sunitinib and staurosporine, the anticholinergic imipramine, and the acetylcholine esterase inhibitor (and former anti-schistosomal drug) metrifonate. Notably, very little degeneracy was observed at the first time point, except for sunitinib at the two highest concentrations. After 24 h, *d*_M_ remained elevated relative to DMSO and degeneracy became apparent for lower concentrations of sunitinib, staurosporine, and the 3-hydroxy3-methyl-glutaryl-coenzyme A reductase inhibitor simvastatin. Finally, by 48 h, concentration-dependent degeneracy became apparent for five of the compounds with the notable exceptions of PZQ and metrifonate. The absence of cidal activity for these two drugs was consistent with their primary activity as paralytics (see below). Thus, the ability to capture phenotypic changes by *d*_M_ (i) afforded a rapid and deep assessment of anti-schistosomal activity that was independent of degeneracy/death and (ii) added essential value by identifying highly relevant anti-schistosomals that did not induce degeneracy, including the two clinically used drugs PZQ and metrifonate. Finally, combining static and dynamic descriptors into the *d*_M_ provided a more sensitive readout of phenotypic change than either modality alone (Supplementary Fig. 3).

**Fig. 5:**
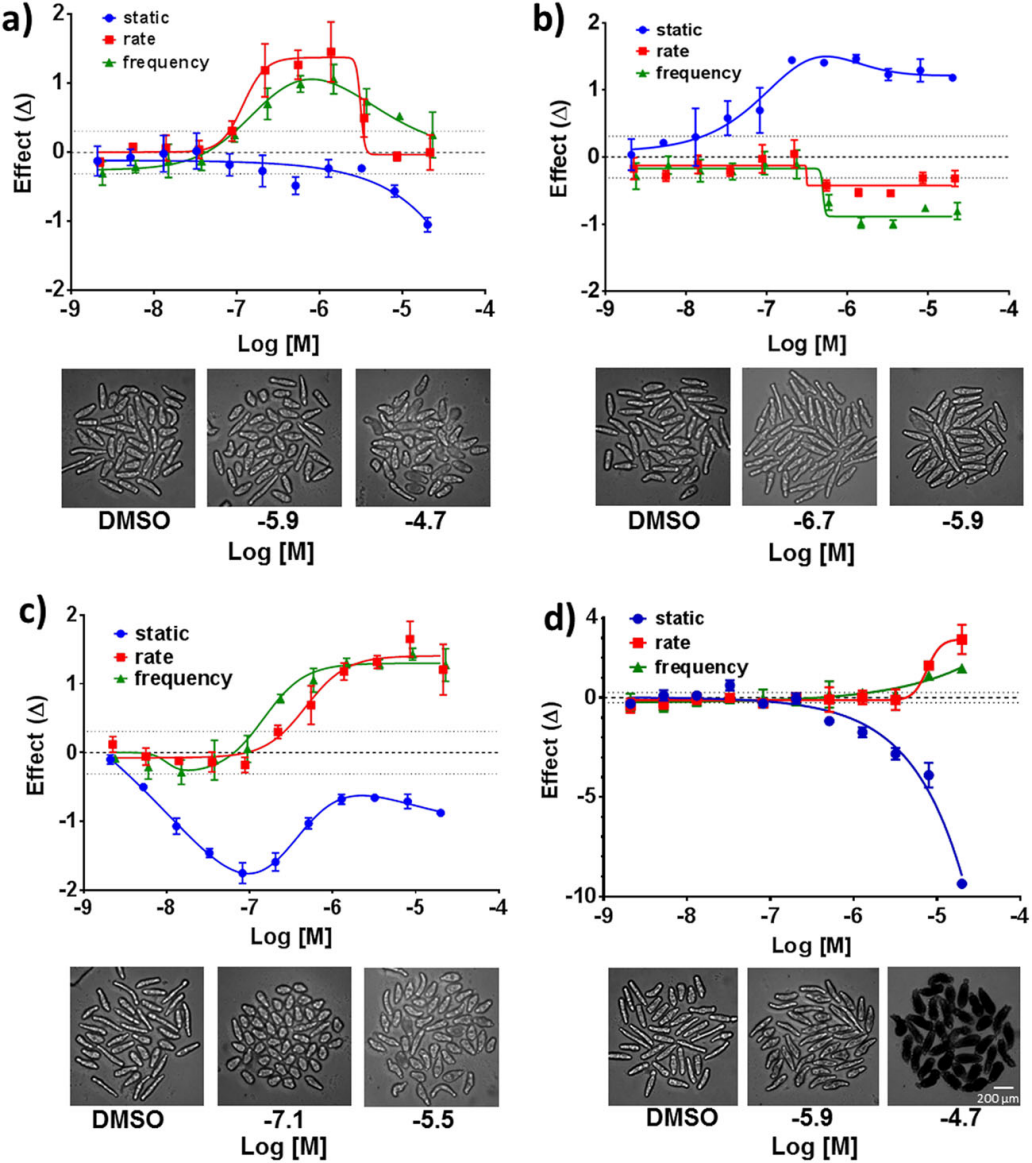
Concentration-dependent phenotypic responses to selected anti-schistosomal drugs from Fig. 4 after 2 h of treatment. Compound concentrations (2 nM–20 µM) are plotted vs. effect size (ES). The graphs display changes in mean length (blue), rate-of-change of length (red), and frequency of changes in length/contractions (green) for **a** l-imipramine, **b** metrifonate, and **c** PZQ. **d** The graph for sunitinib displays changes in mean density levels (blue), rate-of-change of density levels (red), and frequency of changes in density levels (green). Error bars represent the SD from four replicate wells at each concentration. Dotted lines above and below baseline represent 3 SD from the mean of the DMSO-treated wells. Images of the somules at selected drug concentrations are shown below each graph.

Our imaging platform quantified drug-induced increases and decreases in parasite motility. Previously, L-imipramine was visually assessed to induce hypermotility^14^. We confirmed this finding and, for the first time (to our knowledge), quantified the response. As shown in Fig. 5a, imipramine induced a concentration-dependent increase in movement after 2 h between 10 nM and 1 µM (EC_50_ = 100 nM) followed by decreased motility at higher concentrations. This hypermotility was measured as an increasing rate-of-change in length (ES = 1.35; 6 µm/s at ~1 µM vs. 2 µm/s for DMSO) and increasing frequency (ES = 1; 0.36 Hz at 1 µM vs. 0.21 Hz for DMSO). Importantly, the fitted median length never exceeded the minimum or maximum value of DMSO-treated somules at concentrations <8 µM (within ±0.31 ES); hence, had we only relied on static image-based analysis, imipramine might have been missed as an active compound. By contrast, metrifonate at 2 h (Fig. 5b) caused flacid paralysis (ES_frequency_ = −1; 0.09 Hz at 1.3 µM) that was mirrored by an increase in length (ES_static_ = = 1.5; 166 µm for 1.3 µM metrifonate vs. 137 µm for DMSO). This paralysis was consistent with the inhibition of acetylcholine esterase by metrifonate^23^. For worms treated with PZQ or metrifonate, *d*_M_ remained high at 24 and 48 h at concentrations where degeneracy remained low.

Despite the centrality of PZQ for the treatment of schistosomiasis, its mechanism of action is not completely elucidated and is likely complex, leading to Ca^++^ influx and spastic paralysis^8,24^. This complexity is reflected in our phenotypic analysis (Fig. 5c). After 2 h, PZQ exhibited a concentration-dependent increase in rate of movement and frequency, a phenotype consistent with PZQ’s known spastic paralytic effect^25^. These measurements peaked at 5 µM (EC_50_ = 389 nM and 196 nM, respectively). The shift between frequency and rate, which we attribute to the greater sensitivity of the frequency mode for these shorter somules, is noteworthy (see also Fig. 3). Also, PZQ showed a concentration-dependent shortening of the parasite that reached a minimum length of 89 ± 3.6 µm at 82 nM (compared to 141 µm for DMSO; ES = −1.7) after which a partial lengthening occurred. The shortening was observed at a fivefold lower concentration than that needed to increase rate and frequency (EC_50_ = 27 nM for first inflection of the PZQ static-length bell curve and EC_50_ = 147 nM for frequency length). The difference in concentrations may point to more than one molecular target/mechanism of action for PZQ.

The time dependence of the shortening effect by PZQ was also different from that of the spasticity. Although both were observed at 2 h (see above), the shortening effect disappeared by the 48 h time point (Supplementary Fig. 4f), whereas spasticity remained unchanged (Fig. 3f). These data highlight the ephemeral or transient nature of some phenotypic responses, a concept that has not yet been considered in anthelmintic screening. Overall, the imaging methodology, as interrogated through SchistoView, allows for the orthogonal identification and quantification of individual concentration- and time-dependent changes.

For the other four members of the seven-drug test set, phenotypic effects at 2 h preceeded degeneracy/death that were recorded at later time points (Fig. 4). For example, two known anti-schistosomal agents simvastatin^17^ and K11777^26^ induced gradual increases in degeneracy (simvastatin EC_50_ = 1 µM at 24 h; K11777 EC_50_ = 20 μM at 48 h). Degeneracy caused by staurosporine was apparent by 24 h and extended across the entire concentration range (Fig. 4), consistent with this inhibitor’s high affinity for multiple kinases. Other changes included increased area (65% larger than clear worms in DMSO and 13% larger than degenerate worms in DMSO) and increased median diameter (51% larger than clear worms in DMSO and 20% larger than degenerate worms in DMSO; Supplementary Fig. 2, row D). Interestingly, within 2 h, sunitinib produced a gray to jet-black phenotype that was much darker than degenerate worms in DMSO (Fig. 5d). This static phenotype (density levels) was significant at lower concentrations than the change in rate. As with PZQ, the complexity of these changes may reflect the time- and concentration-dependent engagement of different targets^27^.

### Using multi-dimensional features for primary screening

**Fig. 6:**
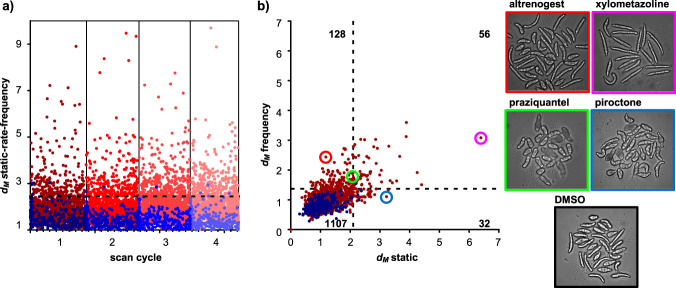
Scatter plot of data from a primary screen of 1323 approved drugs. **a** Twenty plates were imaged over four scan cycles, each representing approximate intervals of 24 h. DMSO controls are shown in blue and the tested drugs (10 µM) are in red. *d*_M_ values combine static, rate, and frequency modes. The horizontal dashed line represents the *d*_M_ value (2.47) that is 3 SD from the mean of the DMSO controls across the four scan cycles. This *d*_M_ value differed by <10% between individual scan cycles. The number of drugs with *d*_M_ ≥ 2.47 are 237, 263, 326, and 309 compounds for scan cycles 1 through 4, respectively. **b** Scatter plot showing contributions of *d*_M_ values based on static (*x*-axis) vs. frequency (*y*-axis) modes for the first scan cycle. The dashed lines represent the *d*_M_ values that are 3 SD from the DMSO mean (2.1 for *d*_M_ for static and 1.4 for *d*_M_ for frequency). The number of drugs in each quadrant is indicated in gray font: 1107 drugs were inactive; 32 drugs induced static phenotypes only, 128 induced only kinetic phenotypes, and 56 compounds induced significant changes in both modes. The frames of the images to the right are color-matched with the highlighted compounds in the plot: note the remarkable range of phenotypes presented by this parasite.

In addition to phenotyping based on inspection of individual descriptors, the screening platform and the SchistoView repository are applicable to high-throughput screens using *d*_M_. We prepared 20 × 96-well plates with 40 somules/well that were incubated with DMSO (0.1%) or 10 µM compound from an in-house collection of 1323 drugs approved for human use. Using the same sample preparation and imaging conditions as for the seven-drug set, plates were robotically handled without manual intervention. Screening proceeded at a maximum rate of one plate/37 min for four scan cycles. Images were automatically processed and analyzed, and data entered into the MySQL database. The screen generated 59,867,820 measurements for 553,492 segmented worms. The *d*_M_ values, calculated from the combined static, rate, and frequency data, were then extracted and plotted in Fig. 6a.

As *d*_M_ does not have an inherent maximum value, the typical screening metric, Z’, cannot be calculated. However, “hits” are usually picked based on SDs from the mean of the DMSO controls. For the data in Fig. 6a (combining static, rate, and frequency), a *d*_M_ value of 2.47 represents 3 SD from the mean of the DMSO-treated controls. Using this cutoff value, a total of 237, 263, 326, and 309 hits were identified for scan cycles 1 through 4, respectively. Notably, three drugs (PZQ, simvastatin, and sunitinib) from the seven-drug test were also present in this screening set and were identified as active, with *d*_M_ values > 2.47.

The individual contributions of the static and frequency measurements to the identification of hits were illustrated by calculating a *d*_M_ for each measurement in scan cycle 1 (Fig. 6b). Of the 216 total hits identified using these individual *d*_M_ values, 128 (59%) of the active compounds differed from DMSO-treated worms only in frequency, 32 (15%) compounds differed only by static measurements, and 56 (26%) showed a significant response by both static and frequency measures. Likewise, when considering rate vs. static *d*_M_, the results also point to the importance of motion-based descriptors. Together, 68% of active compounds differed from DMSO-treated worms based on changes in rate but were not significantly different from DMSO based on static features (Supplementary Fig. 5). Finally, 80 compounds had statistically significant changes in frequency but not rate and 116 compounds had statistically significant changes in rate but not frequency (Supplementary Fig. 6). In summary, quantifying changes in motion was critical for identifying the majority of the active compounds in the drug set.

Phenotypes among the 1323 drug set were remarkably varied. For example, somules exposed to the hormone analog altrenogest displayed static features similar to those of DMSO controls (Fig. 6b), but exhibited a 144% increase in frequency-of-movement by length and a 147% increase in frequency-of-movement by area. Hence, altrenogest was “active” based on the *d*_M_ for frequency. By contrast, the antifungal piroctone only yielded changes in static features with a decreased variation in internal texture (63%) and an increase in form factor (110%, i.e., a more rounded phenotype) relative to DMSO controls. Somules treated with the adrenergic agonist xylometazoline were altered in both static and dynamic descriptors, i.e., a greater mean length of 211 µm vs. 130 µm for controls and a 160% increase in frequency of movement by length, respectively. Consistent with the data in Fig. 3, PZQ-treated somules were shorter (82%) with an increased form factor (140%) compared to DMSO controls (Fig. 6b).

The data obtained for the 1323-member drug set were compared with results from another drug screen that employed an observation-based scoring system (Supplementary Data)^28^. Of the 235 compounds for which data were described^28^, 143 were also screened by us. To compare the screens, we considered the third time point (closest to the 72 h time point used in ref. ^28^) and defined “actives” as those for which *d*_M_ was >2.47 and/or degeneracy was >50%. Of the 143 compounds, 66 compounds were identified as active in both screens, 36 only by us and 11 only in ref. ^28^; 30 compounds were inactive in both screens. Overall, there was a 67% agreement between the screens, i.e., considering both actives and inactives. Interestingly, the two screens differed the most in the category of compounds that we identified by the *d*_M_ metric only: out of 30 actives in this category, only 12 were also identified in ref. ^28^ (40% concordance). Thus, the present screen extends our ability to identify compounds by quantifying live phenotypic changes in the parasite.

## Discussion

The screening platform described here comprises an integrated suite of solutions that solve the bottlenecks hampering drug discovery for global disease pathogens such the schistosome. Issues addressed include the following: (a) producing and dispensing 10^4^–10^5^ parasites/week to enable automated screening; (b) developing a robust image collection and segmentation protocol, and (c) designing a system—SchistoView—to store, visualize, query, and explore the multivariate data. Using the method, we captured the complexity of the schistosome’s response to seven drugs and identified previously unreported screening hits from a drug library. The data highlight the importance of quantifying changes in motion on the seconds timescale. To our knowledge, this scale and quantitative depth have not been achieved before for schistosomes or other parasitic helminths and the method is, in principle, adaptable to other organisms.

Somules are difficult to image due to their (variability in) movement and because they have a low contrast in bright field. We solved the image collection challenge using round-bottom wells to constrain worms into one visual field and thus limit translation. We then addressed their low contrast by focusing slightly below the worm to enhance its outline. From there, we observed two basic classes of worms—clear and opaque (degenerate)—and optimized segmentation protocols for each. The resulting segmentation accuracy (precision of 88% and a recall of 95%) is an improvement on a previous report that employed bright-field analysis for high-throughput screening (24.5 ± 7% segmentation accuracy) where touching somules could not be evaluated^18^. Also, our imaging approach economizes on the number of parasites needed by three- to fourfold and measures how worms move rather than a simple classification of whether movement has occurred^18^. Finally, our methodology provides a solution to the critical issue of segmentation of touching objects in the analysis of bright-field images generally^29–31^.

Our live-imaging platform can be incorporated into a drug discovery pipeline upstream of ex vivo phenotypic screens of adult schistosomes and rodent models of infection^14^. Recent advances in the image-based quantification of adult schistosome motility^32,33^ have demonstrated the ability to quantify motion and could mesh seamlessly with the workflow described here. The platform will also complement other advances relating to schistosome biology, including gene expression profiling^34^, metabolomics^35^, and CRISPR/Cas9^36^, which together will improve our ability to holistically quantify this globally important parasite’s responses to a range of drug-induced environmental and developmental phenotypes. To facilitate such discoveries, the database and SchistoView interface are available online.

## Methods

### Compounds

K11777 was synthesized via a contract research organization^26^; simvastatin (S1792) was purchased from Selleckchem. PZQ (racemic; P4668), metrifonate (45698), and l-imipramine (I0899, HCl) were purchased from Sigma Aldrich. Sunitinib (S-8803 as the malate salt) and staurosporine (S-9300 as the free base) were purchased from LC Laboratories. The library of approved drugs included 1129 compounds from the Microsource Discovery Systems Drug Collection and an additional 194 compounds donated by Iconix or purchased from commercial vendors. The set included drugs approved by the Food and Drug Administration (85%) and by the analogous European and Japanese agencies (15%).

### Ethics statement

Vertebrate animal maintenance and use were performed in accordance with University of California, San Francisco’s Institutional Animal Care and Use Committee protocol AN086607.

### Somule preparation and plating

The *S. mansoni* life cycle (Naval Medical Research Institute (NMRI) isolate) was maintained by intraperitoneal injections of up to 600 infective larvae (cercariae) into 4-to 6-week-old, male Golden Syrian hamsters. Eggs were harvested from hamster livers six weeks later to generate miracidia, which were then used to infect the *Biomphalaria glabrata* (NMRI strain) snail vector. The platform design (Fig. 1) involved the intensive propagation of snails to produce 10^4^–10^5^ infective larvae (cercariae) per week, which were sufficient for up to twenty-five 96-well assay plates. Cercariae were then mechanically converted^14^ into the post-infective schistosomula (somules, ~200 × 100 µm) that are relevant to infection in humans. These somules were used within 2 h of their transformation from cercariae.

Each well of a 96-well u-bottom polystyrene assay plate (Corning, Costar 3799) was pre-wetted with 200 µL ddH_2_0 to prevent the formation of bubbles at the well surface. After aspirating the ddH_2_0, each well received an average of 40 somules in 200 µL Basch medium^37^ supplemented with 4% heat-inactivated fetal bovine serum, 100 U/ml penicillin, and 100 mg/ml streptomycin. Somules (40,000) were suspended in 200 mL of medium using a magnetic tumble stirrer (V&P Scientific, VP 710C1-ALPFX) rotating at 45 r.p.m. and then dispensed with a 96-channel pipette head (Beckman Coulter Biomek FXp) loaded with sterile 165 µL wide bore filter tips (Axygen, FXF-165-WB-R-S). Eight 96-well plates could be prepared per tumbler volume of 200 mL (including a 40 mL dead volume). All assay plates were dispensed in <5 min to minimize tumbling damage to the parasite. Compound in neat DMSO was added to the well at the required final assay concentration in 0.1% DMSO using a 96-channel pin tool fitted with 200 nL slotted hydrophobic pins (V&P Scientific, AFIXFX96FP3). Compound was added while shaking the plate at 1000 r.p.m. in a 0.5 mm radius using a Teleshake 1536 (Variomag), which dispersed the compound from the pin into the surrounding media.

### Robotic handling, imaging, and analysis

The Momentum 2.0 automation scheduler moved each assay plate from the automated tissue culture incubator (ThermoFisher C2, 37 °C, 5% CO_2_) to the barcode reader, then to the automated microscope (GE IN Cell Analyzer 2000), and back to the tissue culture incubator. Each iteration took ~35 min.

A high-content imager (InCell Analyzer 2000; GE Healthcare) was used to collect 20 s of time-lapse images of parasites under one field of view with a ×10 objective. The 4 megapixel charge-coupled device sensor was binned 4 × 4 and the bright-field/4′,6-diamidino-2-phenylindole channel was set to a 3 ms exposure. The focal plane was offset 40 µm from the bottom of the well to thicken the edge (surface) of the worm. The “sit-and-stare” time-lapse schedule began with a 3.5 s delay to allow time for auto-focusing followed by 30 image acquisitions 0.66 s apart.

Images were then analyzed and segmented as described in Supplementary Information, Extended Methods. Features were extracted from the optimal mask chosen from multiple segmentation attempts for each worm and were stored in a custom MYSQL database for visualization in SchistoView.

### Statistics and reproducibility

Microsoft VBA was used to perform all statistical analysis. The method monitors each worm as a separate object. Glass ES^19,20^ and *d*_M_^21^ for the seven-drug set were based on the average values from 160 somules (4 experimental wells, each with ~40 worms). Error bars shown in Fig. 5 represent SDs in ES from 4 wells, each containing ~40 worm objects. Dotted lines above and below baseline represent three SDs from the mean of the DMSO-treated wells. Data shown are representative of at least three independent experiments. Glass ES and *d*_M_ for the 1323-compound drug set were calculated from ~40 worms/test compound in one well. Dotted lines in Fig. 6 represent three SDs from the mean of the DMSO-treated wells.

### Reporting summary

Further information on research design is available in the Nature Research Reporting Summary linked to this article.

## Supplementary information

Supplementary Information

Description of Additional Supplementary Files

Supplementary Data

Reporting Summary

## Data Availability

Supplementary materials include Supplementary Information (Supplementary Table 1, Supplementary Figures, and Extended Methods detailing data collection, segmentation, and database protocols) and Supplementary Data (1323 drug screen, hits, and non-hits). The data of the seven-drug dose–response screen and the 1323-compound drug screen are available through SchistoView (http://haddock9.sfsu.edu/schistoview/home). Any remaining data can be obtained from the corresponding authors upon reasonable request.
