# Supplementary material for: A multi-dimensional, time-lapse, high content screening platform applied to schistosomiasis drug discovery
Source: Commun Biol. 2020 Dec 21;3:747. doi: 10.1038/s42003-020-01402-5 (PMC7752906; doi:10.1038/s42003-020-01402-5)
Supplement: Supplementary file 1 — Supplementary Information [file 42003_2020_1402_MOESM1_ESM.pdf]

Supplementary Information

A multi-dimensional, time-lapse, high content screening platform applied to schistosomiasis drug discovery

Steven Chen[1], Brian M Suzuki[2,3], Jakob Dohrmann[4], Rahul Singh[4]*, Michelle R Arkin[1]* & Conor R Caffrey[2,3*]

[1]Department of Pharmaceutical Chemistry and Small Molecule Discovery Center, University of California, San Francisco, CA 94143; [2]Center for Discovery and Innovation in Parasitic Diseases, Department of Pathology, University of California, San Francisco, CA 94158; [3]Center for Discovery and Innovation in Parasitic Diseases, Skaggs School of Pharmacy and Pharmaceutical Sciences, University of California, San Diego, La Jolla, CA 92093; [4]Department of Computer Science, San Francisco State University, San Francisco, CA 94132

*correspondence to: rahul@sfsu.edu; michelle.arkin@ucsf.edu; ccaffrey@health.ucsd.edu.

Contents

Supplementary Table 1	page 3
Supplementary Figure 1	page 5
Supplementary Figure 2	page 7
Supplementary Figure 3	page 8
Supplementary Figure 4	page 9
Supplementary Figure 5	page 10
Supplementary Figure 6	page 11
Extended Methods	page 12

Supplementary Table 1. Glossary of Terms.

Term	Description	Units
**Area**	Area of a target	µm^2
**Median Diameter**	The median internal distance perpendicular to the maximum curved chord.	µm
**Length**	Maximum distance across a target. Boundaries may be crossed.	µm
**Form Factor**	Estimate of circularity, expressed as a value between 0 and 1 (1 equals a perfect circle).	value
**Perimeter**	Distance around a target.	µm
**Straight Chord**	The maximum straight-line distance across a target without crossing a boundary.	µm
**Curved Chord**	Maximum center line through target.	µm
**Bend**	Bend = (max curved chord / max straight chord).	ratio
**Pinch**	Pinch = (median diameter * length) / area) is the estimated area divided by the actual area.	ratio
**Wave**	Wave = Perimeter / (2 * Area^$^{0.5}$) is the actual perimeter divided by the estimated perimeter.	ratio
**Mass**	The sum of all pixel values in the shape.	sum
**WMOI**	(Weighted Moment of Inertia) Index of the homogeneity of density levels (gray levels) within a circular target. A value of 1 indicates the target is relatively homogeneous. If >1, the target has a higher proportion of bright pixels in its center. If <1, the target has a higher proportion of bright pixels around its perimeter.	index
**Density levels**	Gray level intensity, where black = 0 and white = 4095 (12-bit image).	Levels
**SD - Levels**	A standard deviation (SD) of pixel densities, which measures the pixel density variation within the target.	levels
**Angle**	Angle of the straight chord relative to the horizontal (horizontal = 0 degrees). Negative values are clockwise from horizontal; positive values are counter-clockwise.	degrees

**Rate**	The average amount of changes between sequential time-lapse images.	"units"/frame
**Frequency**	The number of times a feature changes direction.	Hz
**Effect Size (ES)**	ES = (x-μ)/σ where x is the drug mean, μ is the DMSO mean, and σ is the standard deviation of DMSO. No unit, but the magnitude of the measurement can be thought of as the number of standard deviations from the DMSO control group for the selected feature.	
**Mahalanobis Distance (d_M)**	d_M is a multi-dimensional effect size which measures the distance of a test point from a reference distribution. No unit, but the magnitude of the measurement can be thought of as the number of standard deviations from the DMSO control group.	

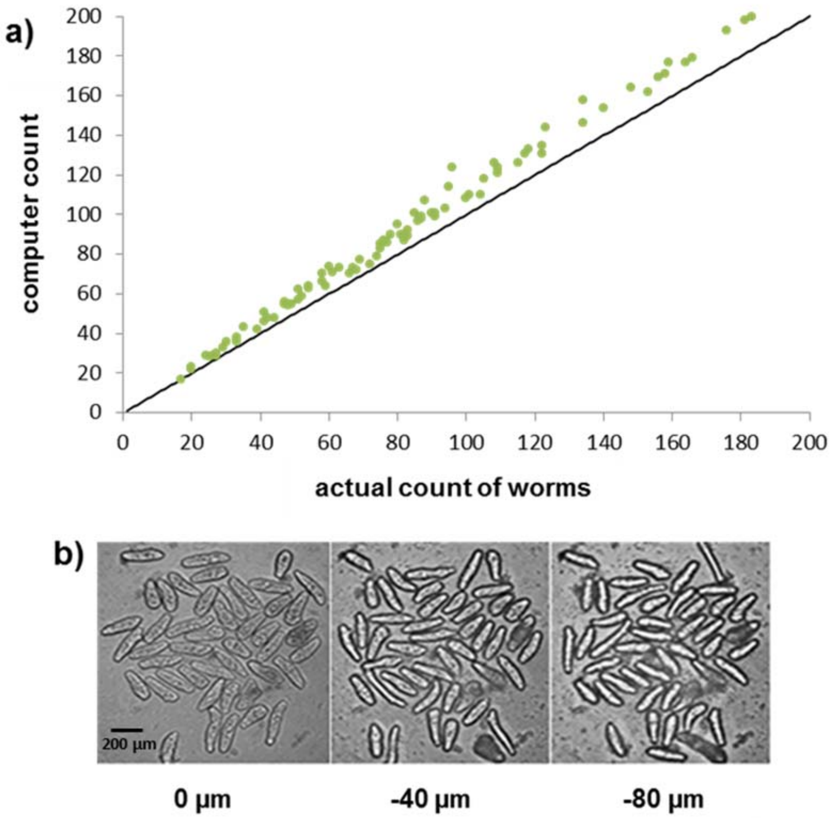

c)

Time (h)	Precision	Recall	DMSO/DRUG treated worms used
2	0.93	0.97	19,567
24	0.86	0.97	19,643
48	0.88	0.89	19,246
**Time Range (h)**	**Precision**	**Recall**	**Total Worms**
2 - 48	0.88	0.95	58,456 (40.6 worms/well over 1440 wells)

d)

Time (h)	%DMSO Treated Worms Overlapped	%DMSO Treated Worms Not Analyzed Due To Overlap	DMSO Treated Worms Used in Overlap Analysis
2	0.053	0	3,764
24	0.053	0.053	3,754
48	0.47	0.31	3,598

Supplementary Figure 1. Optimizing parasite handling and segmentation. **(a)** Comparison of actual somules (counted by visual inspection) and the number of somules identified by the object classifier algorithm 'computer count' (see **Extended Methods**, below). Computational inclusion of non-worm objects with worm-like features leads to a systematic 10% increase in object count. **(b)** Images from a single sample well imaged at three focal planes (0, -40, and -80 μm from the outside bottom of the well). Lowering the focal plane improves the contrast of the somule outline ('edge'); at -40 μm the appearance of the outline is improved while some of the internal texture detail is preserved. **(c)** Precision and recall were determined by manual inspection of the 58,456 somules that were screened in the seven-drug set. **(d)** Somule overlap frequency and data removal due to overlap events. The overlap increases from 0.05% to 0.3-0.47% between 24 and 48 h, potentially reflecting growth of the somules, increased degeneracy or increased motility.

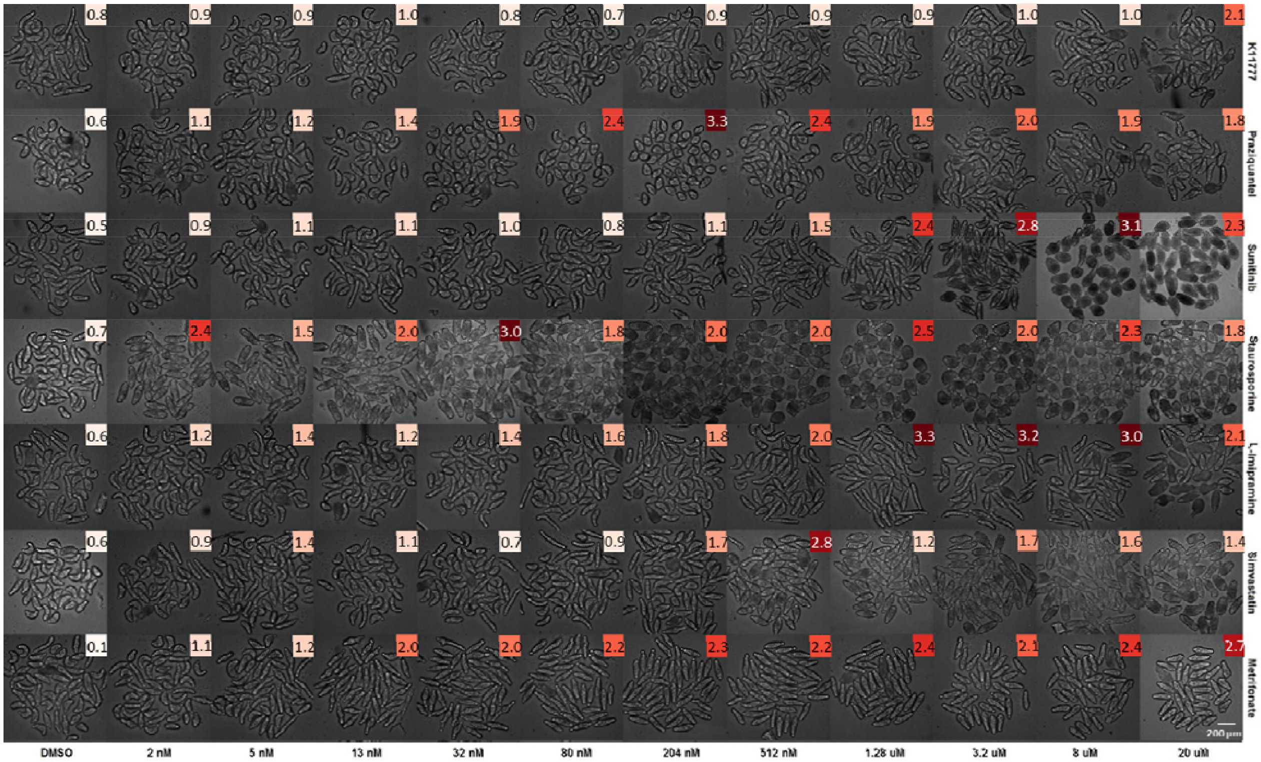

Supplementary Figure 2. Montage of somule images 24 h after treatment with seven test drugs. Drug names are to the right and concentrations are at the bottom. Each image in the montage is labeled with the corresponding d_M value and scaled color.

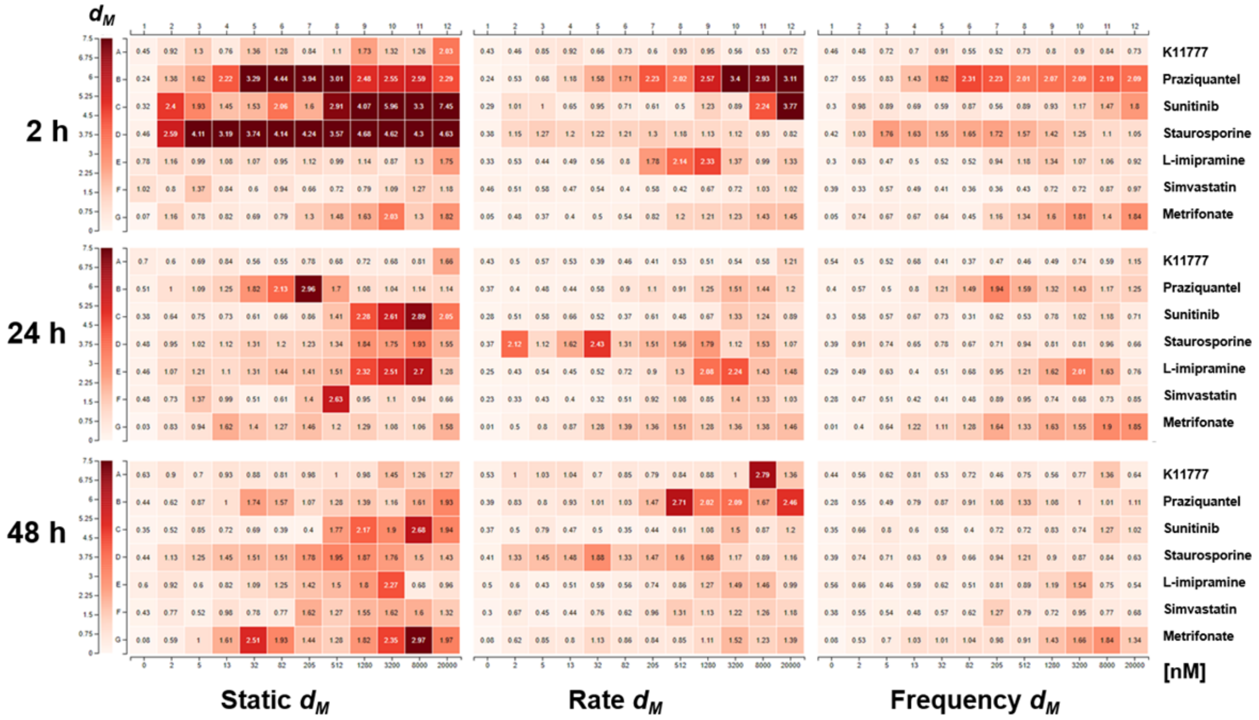

Supplementary Figure 3. Differing sensitivities of d_M measurements in the static, rate and frequency modes. d_M values at 2, 24 or 48 h after treatment were measured using only static features (left panels), rate (center panels) or frequency for seven test drugs. Drugs were arrayed over an 11-point 2.5-fold dilution range from 2 nM to 20 µM. Values were determined from the aggregation of four wells per treatment. Note that the d_M values shown do not necessarily smoothly change with increasing dose of drug. This complexity reflects the observation that multiple parameters show maximum changes at different concentrations. Each mode offers a differential sensitivity to measuring changes in the somule; *e.g.*, the static d_M for staurosporine across all concentrations after 2 h, the rate d_M for imipramine (205 – 1280 nM) after 2 h and the frequency d_M for metrifonate at 8 and 20 µM after 24 h.

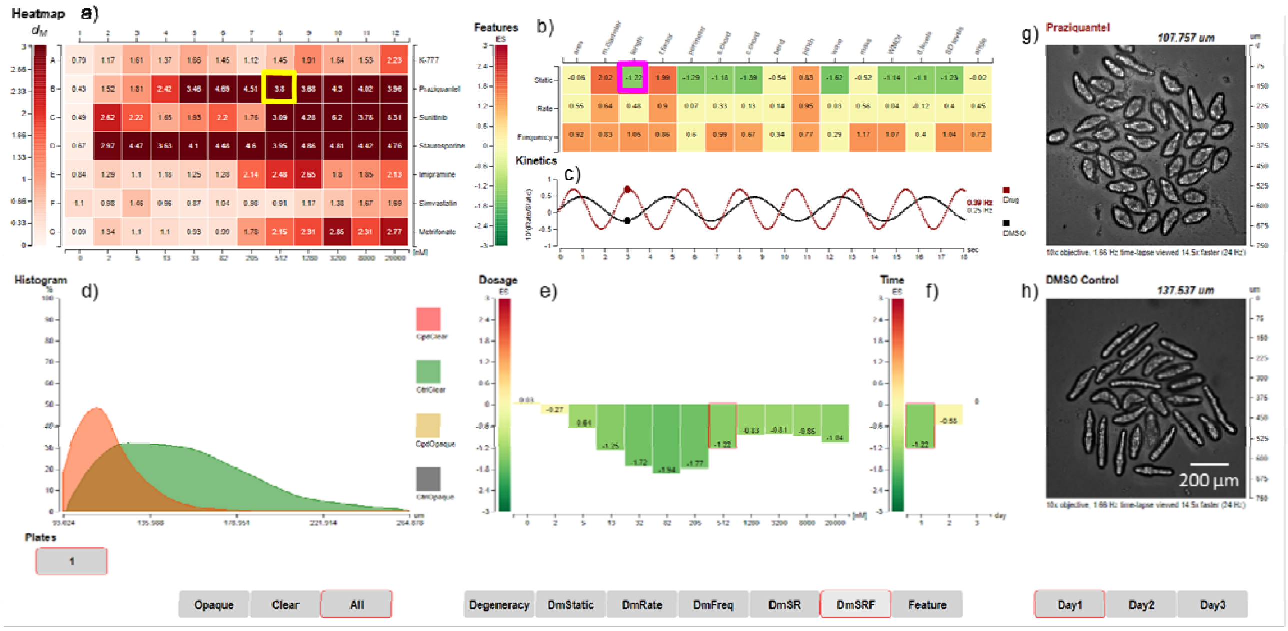

Supplementary Figure 4. Screenshot of the SchistoView graphical user interface. The figure is analogous to **Fig 3**, but highlights the length of PZQ-treated somules whereas **Fig 3** shows the frequency of changes in length. Selected data are shown to illustrate the hierarchical approach to visualization. **(a)** Heat map of Mahalanobis distances (d_M) for seven test drugs arrayed over an 11-point 2.5-fold dilution series from 2 nM in column 2 to 20 µM in column 12. Drugs, from top to bottom, are, K11777, PZQ, sunitinib, staurosporine, imipramine, simvastatin and metrifonate. DMSO controls are arrayed in column 1 and are shown as the average d_M (0.77) for all DMSO controls. A d_M of 1.61 is significantly different (3 SD) from control. Clicking on coordinate B8 (identified by the yellow square: 512 nM PZQ) populates panels (**b**) and (**g**) (see below). **(b)** Heat map showing the effect sizes (ES) for static, rate and frequency, after exposure to 512 nM PZQ for 2 h, *i.e.*, the selected well from (**a**). Three sets of 15 features are arrayed in rows and columns, respectively. Clicking on the intersection of the length feature and static mode (magenta box) in (**b**) populates panels (**c**) through (**f**) and the underlying data. (**c**) Calculated waveforms defined by the range of length (amplitude) and frequency of length contraction (frequency). DMSO control worms are slower moving (lower frequency) than those treated with 512 nM PZQ (red line). (**d**) Histogram displaying the distribution of static length for DMSO control worms (green) and PZQ-treated worms (orange). (**e**) Bar graph depicting the ES for static length after PZQ treatment across 11 concentrations (second row in (**a**)). (**f**) Bar graph depicting the ES for static length in the 512 nM PZQ treatment across the three days of measurement. **(g)** First image from time-lapsed movie of the well highlighted in (**a**); in the live SchistoView, the 30-frame movie is looped. (**h**) as for (**g**) except for the DMSO control.

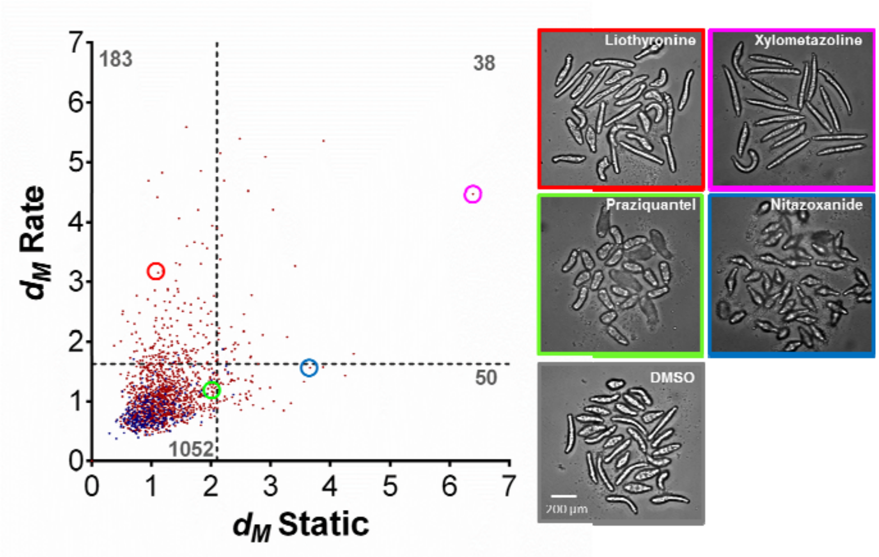

Supplementary Figure 5. Scatterplot of $d_{M(rate)}$ *vs*. $d_{M(static)}$ for a primary screen of 1,323 approved drugs. Like **Fig 6b** in the main text, which shows a scatterplot of $d_{M(frequency)}$ *vs*. $d_{M(static)}$, the screen was performed at 10 µM. The data shown are from the first scan cycle approximately 24 h after the addition of drug. The dashed lines represent the d_M values that are 3 SD from the DMSO mean (2.1 for $d_{M(static)}$ and 1.6 for $d_{M(rate)}$). The number of drugs in each quadrant is indicated in dark grey for both static and rate modes: 1,052 drugs were inactive, 50 drugs induced static phenotypes only, 183 induced only kinetic phenotypes and 38 compounds induced changes in both modes. The frames of the images to the right are color-matched with the highlighted compounds in the plot: note the remarkable range of phenotypes presented by this parasite.

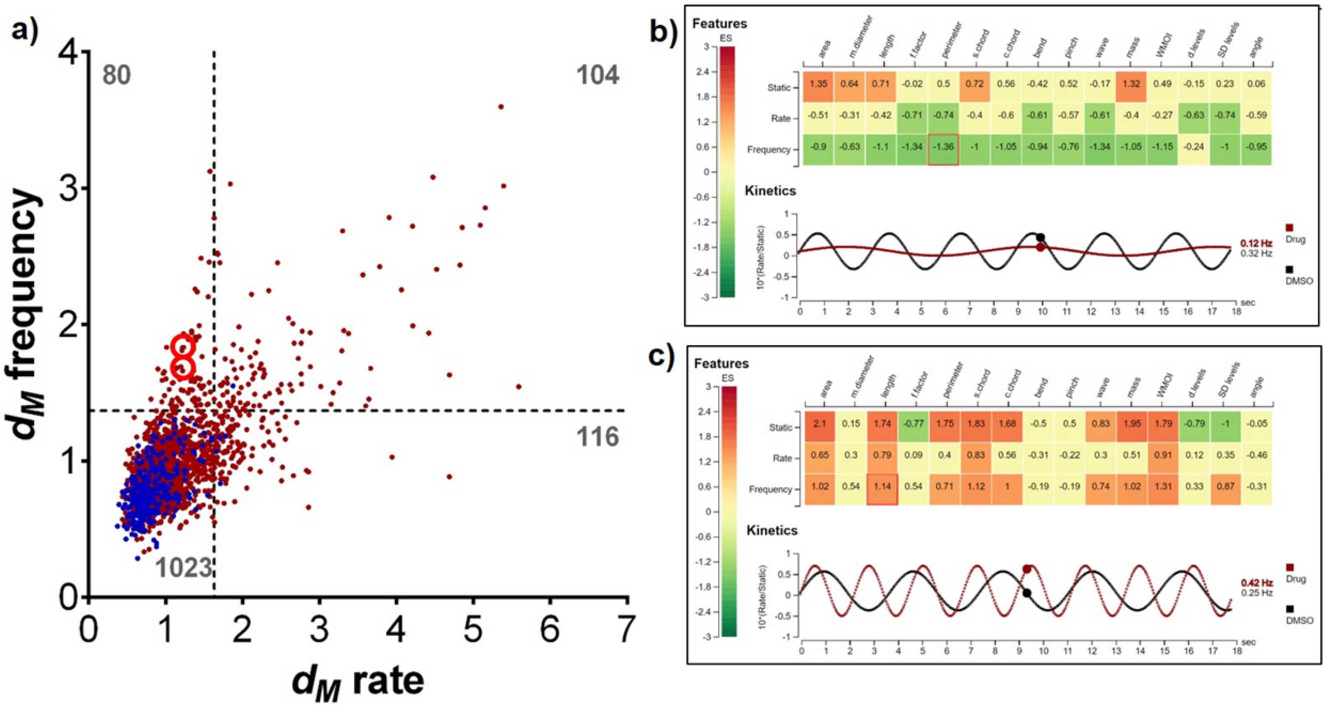

Supplementary Figure 6. (**a**) Scatterplot of $d_{M(frequency)}$ *vs*. $d_{M(rate)}$ for a primary screen of 1,323 approved drugs. Like **Fig. 6b** in the main text which shows a scatterplot of $d_{M(frequency)}$ *vs*. $d_{M(static)}$, the screen was performed at 10 µM. The data shown are from the first scan cycle approximately 24 h after the addition of drug. The dashed lines represent the d_M values that are 3 SD from the DMSO mean (1.6 for d_M for rate and 1.4 for d_M for frequency. The number of drugs in each quadrant is indicated in dark grey for both frequency and rate modes: 1,023 drugs were inactive, 116 drugs induced significant rate-based phenotypes only, 80 induced phenotypes associated with frequency only and 104 compounds induced changes in both modes. Two drugs, vilazodone and apomorphine, are marked with upper and lower red circles, respectively. (**b, c**) Examples of drugs that induce more changes in frequencies than changes in rates as shown by the table of Features (effect sizes) and Kinetics (red = drug; black = DMSO). Apomorphine (**b**) induces a hypomotile phenotype, whereas vilazodone (**c**) generates hypermotility. Images taken from SchistoView.

<u>Time-lapse Image Acquisition</u>

Open the automation scheduler (Momentum 2.0) with instructions to move each assay plate from the automated tissue culture incubator (Thermofisher C2, 37ºC, 5% CO_2) to the barcode reader, then to the automated microscope (GE IN Cell Analyzer 2000), and then back to the tissue culture incubator. One cycle takes approximately 35 minutes or the time it takes to scan one assay plate in the automated microscope.

1. Place the 96-well round bottom polystyrene assay plate with lid (Costar 3799) into the nest of a GE IN Cell Analyzer 2000 (software version 3.0.0.43).
2. Open the acquisition protocol with the following settings (from XDCE):
    a. Objective
        i. Focal length = 20.0
        ii. Id = 12111
        iii. Lineartype = 7
        iv. Magnification = 10
        v. Numerical Aperture = 0.45
        vi. Objective Name = 10X/0.45, Plan Apo, CFI/60
        vii. Pixel height = 2.96
        viii. Pixel width = 2.96
        ix. Refractive index = 1.0
        x. Unit = µm
    b. Polychroic
        i. QUAD1 (any polychroic will do)
    c. CCD Camera
        i. Size; height = 2048, width = 2048
        ii. Flat Field Correction = False
        iii. Binning value = 4 x 4
        iv. Bias value = 144.21
        v. Gain value = “”
    d. Wavelength
        i. Imaging mode = 2-D
        ii. Excitation Filter = TL-Brightfield, 473 nM
        iii. Emission Filter = DAPI, 455 nM
        iv. Exposure time = 3 ms
        v. HWAFOffset = 0 µm
        vi. FocusOffset = -50 µm
    e. Software Auto Focus = False
    f. Laser Auto Focus = True
    g. Plate dimensions as entered in software
        i. Columns = 12, Rows = 8
        ii. Plate height = 14.16 mm
        iii. Bottom thickness = 1310 µm

        iv. Bottom interface = plastic
        v. Bottom height = 2.15 mm
        vi. Well volume = 360 uL
        vii. Well parameters = Round, Size = 6.35 mm
        viii. Top Left Well Center Offset ; horizontal = 13.8 mm, vertical = 11.7 mm
        ix. Well Spacing; horizontal = 9.0 mm, vertical = 9.0 mm
    h. Plate heater use = false
    i. Plate map, acquisition mode
        i. Layout; columns = 1, rows = 1
        ii. Direction value = horizontal snake
    j. Time schedule = enabled
        i. Incubate between time points = false
        ii. Mode = spit and stare
        iii. Time points in ms = 0, 3740, 4340, 4940, 5540, 6140, 6740, 7340, 7940, 8540, 9140, 9740, 10340, 10940, 11540, 12140, 12740, 13340, 13940, 14540, 15140, 15740, 16340, 16940, 17540, 18140, 18740, 19340, 19940, 20540, 21140
            1. This first time point = 0 is the point at which the autofocus operation occurs. To allow time for this operation to complete, the next time point occurs at 3740 ms and continues at a rate of 600 ms between time lapse images. In total, there were 1 autofocus image and 30 time lapse images.

3. Write the image stack to an E-SATA connected storage device (preferably RAID) capable of storing 1.44 GB per assay plate per day.

The exposure time of 3 ms (step 2.d.iv) is the shortest exposure time the GE IN Cell Analyzer 2000 will accept. With the CCD camera binning value set to 4 x 4 (step 2.c.iii), some of the pixels in the image may be saturated (>= 4095). The binning value of 4 x 4 allowed for the fastest time lapse acquisition of 600 milliseconds since a smaller array is faster to readout. The possibility of saturated pixels was accepted in return for a faster frame rate.

Image Segmentation *(Protocol: Schisto94)*

Open IN Cell Developer Toolbox 1.9 which can be found in IN Cell Investigator 1.6, a suite of software that comes with the GE IN Cell Analyzer 2000 automated microscope. From the “analysis” tab, select “batch analysis manager…” and “add…”, select the “Schisto94_ba” protocol, and image folders for analysis. Then select “Run batch analysis…”

There are three main workflows: clear body outliner, tegument outliner, dark body outliner. These workflows are imaging preprocessing macros which transform the image prior to segmentation. Each workflow produces 4 target types: threshold 1, threshold 2, threshold 3, and a merge of the results from threshold 1 to 3. Each target set records 17 features: area, x, y, diameter, length, form factor, perimeter, straight chord, curved chord, bend (a user defined feature), pinch (a user defined feature), wave (a user defined feature), mass, weighted moment of inertia, density-levels, sd-levels, and angle. The data is recorded at the cell level, or in this case, per organism.

The purpose of this segmentation is to cast a large net around a large variety of objects where up to 50% could be artefactual (i.e. inter-organism objects, intra-organism objects). This inefficiency is

by design in order to lower the false negative rate in segmentation. The false positives (artifacts) are detected and removed in a subsequent data processing step external to IN Cell Developer 1.9

Before any of the workflows are run, the raw image is processed with a custom built flat field correction (FFC) image preprocessing macro. All subsequent processing and analysis will be based on the flat field corrected image. (The flat field correction is the first step in the Macro*: schisto94_n_master.*)

1. **Flat Field Correction** (Macro: *Schisto94_a_FFC*) (**Figure 1**). Divide the raw image by an estimate of the background of each image to produce a flat field correction. The steps below outline how an estimate was generated. (Code enumerated in roman numerals) (8 operations per image)
    a. Load raw image.
    b. Transform result from step “1.a” with transform filter = median, kernel size = 5
    c. Transform result from step “1.b” with transform filter = local arithmetic
        i. kernel = 99;
        ii. src = IAve;
    d. Apply a transform point operation = arithmetic (two src) where source1 = result from step “1.b” and source2 = result from step “1.c”.
        i. Sel(abs(src1-src2)/src2<0.025,src1,0);
    e. Transform result from step “1.d” with transform filter = max, kernel size = 33
    f. Transform result from step “1.e” with transform filter = local arithmetic
        i. kernel = 51;
        ii. src = IAve;
    g. Transform result from step “1.f” with transform filter = local arithmetic
        i. kernel = 67;
        ii. src = IAve;
    h. Apply a transform point operation = arithmetic (two src) where source1 = result from step “1.a” and source2 = result from step “1.g”.
        i. (src1/src2)*2048;

The round wells of the 96-well assay plate produce varying backgrounds due to the position of the well in the plate, the artefacts in the plastic, and changes in illumination due to the grayness of the sample. Therefore, an *in situ* background estimate was used. Flat field correction and centering of the pixel values in each image allows for the universal application of object detection thresholds based on fixed values.

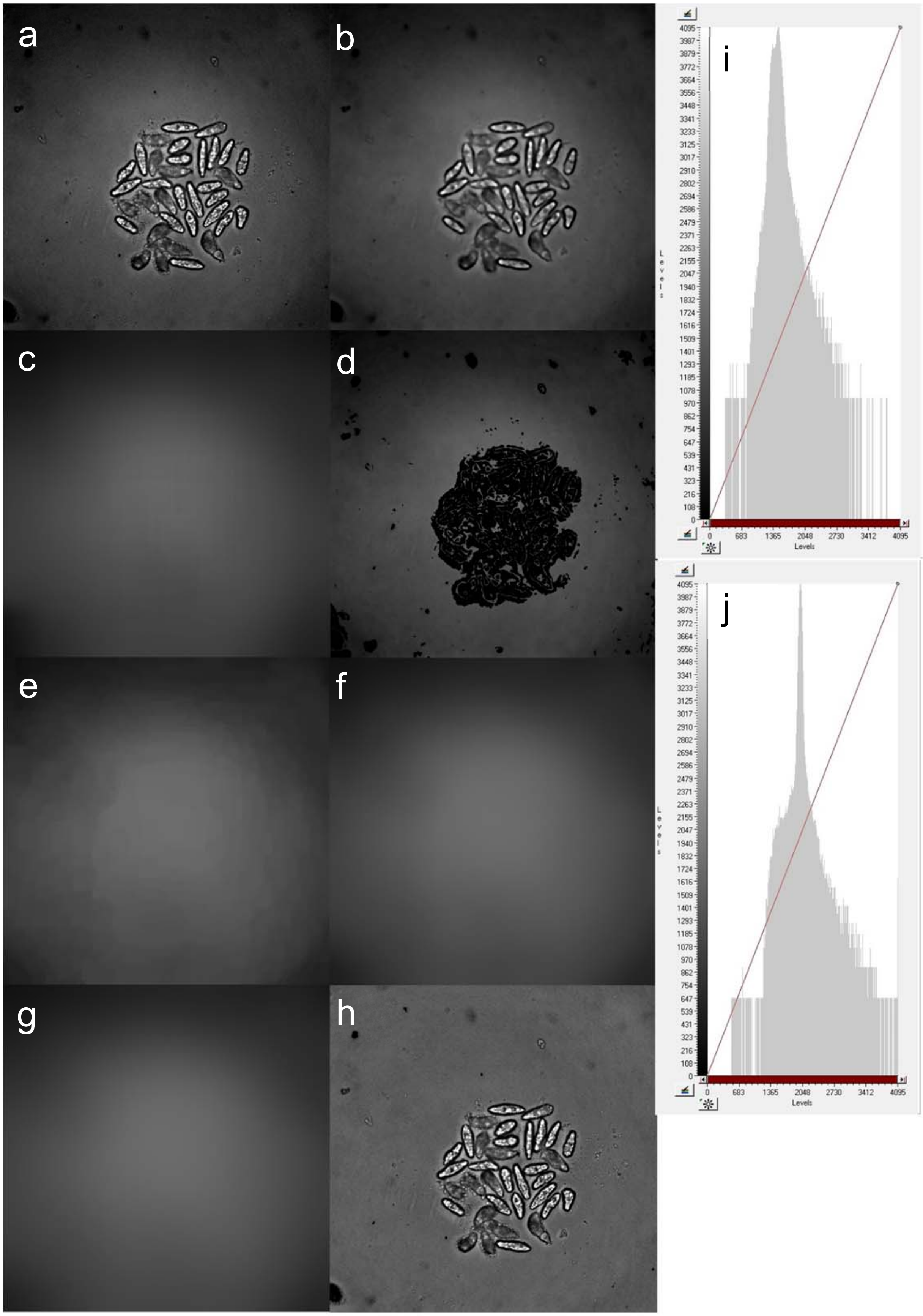
a
b
c
d
e
f
g
h
i
j
Levels
0
683
1365
2048
2730
3412
4095

Figure 1, Flat Field Correction using an *in situ* background estimate. a) raw image, b) median transform of raw image (light smoothing), c) local arithmetic transform of raw image (heavy smoothing), d) transform point operation which sets pixel in panel "b" to zero if pixel is > 2.5% different from the same pixel in panel "c", e) max transform promotes background pixels between and around zeroed pixels in panel "d", f) local arithmetic transform smooths result from panel "e" , g) local arithmetic transform smooths result from panel "f", h) transform point operation which divides the raw image by the background estimate in panel "g" and then multiplies the result by 2048 to center the image pixel values within the 12-bit range, i) histogram of the raw image, j) histogram of the flat field corrected image.

The next macro is called “clear body outliner” because the outlines produced are based on image thresholding which target the white translucent area of the organism.

2. **Clear Body Outliner Preprocessing** *(*Macro: *schisto94_n_master*) (Figure 2) (53 operations per image)
    a. Transform FFC image with transform filter = local arithmetic *(*Macro: *schisto94_n_1950)*:
        i. sel(src<1950,4095,0);
    b. Transform result from step “2.a” with transform filter = sieve (binary), retain objects > 500 µm^2
    c. Transform result from step “2.b” with transform filter = closing (binary), kernel = 3
    d. Transform result from step “2.c” with transform filter = thinning, passes = 3
    e. Transform result from step “2.d” with transform filter = pruning, passes = 3
    f. Transform result from step “2.e” with transform filter = inversion
    g. Transform result from step “2.f” with transform filter = sieve (binary), retain objects > 2600 µm^2
    h. Repeat steps “2.a” through “2.g” with following code for step “2.a”, (Macro: *schisto94_n_1800*):
        i. sel(src<1800,4095,0);
    i. Repeat steps “2.a” through “2.g” with following code for step “2.a”, (Macro: *schisto94_n_1700*):
        i. sel(src<1700,4095,0);
    j. Reset a channel with transform filter = local arithmetic
        i. src = 0;
    k. Transform result from “Macro: *schisto94_n_1950*” with transform filter = dilation (binary), kernel = 3
    l. Apply a transform point operation = arithmetic (two src) where source1 = result from threshold “Macro: *schisto94_n_1950*” and source2 = result from step “2.k”.
        i. src2-src1;
    m. Apply a transform point operation = arithmetic (two src) where source1 = result from step “2.j” and source2 = result from step “2.l”. The result is added to the channel used in step “j”.
        i. src2+src1;
    n. Repeat steps “2.k” through “2.m” with result from “Macro: *schisto94_n_1800*”.
    o. Repeat steps “2.k” through “2.m” with result from “Macro: *schisto94_n_1700*”.
    p. Transform merge result (channel in step “j”) with transform filter = inversion

q. Transform result from step “2.p” with transform filter = sieve (binary), retain objects > 100 μm^2

r. Transform result from step “2.q” with transform filter = inversion

s. Transform result from step “2.r” with transform filter = erosion, kernel = 3

t. Transform result from step “2.s” with transform filter = sieve (binary), retain objects > 100 μm^2

u. Transform result from step “2.t” with transform filter = pruning, passes = 3

v. Apply a transform point operation = arithmetic (two src) where source1 = result from step “2.q” and source2 = result from step “2.u”.

    i. src2+src1;

w. Apply a transform point operation = arithmetic (two src) where source1 = result from step “2.v” and source2 = result from step “1.h”.

    i. sel(src1==4095,src2,0);

Steps “2.a” through “2.i” generate the preliminary target data for threshold1 to threshold3. (**Figure 2A**) The refinement of this preliminary data and consolidation into a fourth target begins with step “2.j”. (**Figure 2B**) The fourth target is based on a merge of the data from the three thresholds. The resulting outline will have extra objects produced by the intersections. The extra objects are filtered to reduce complexity of the merged result and this process starts with step “2.p”. (**Figure 3B**) In addition, the merge result represents all possible objects all clear body target sets and these results are used to patch holes in the results from threshold 1 to 3. A complete set of overlapping objects is a requirement for target linking.

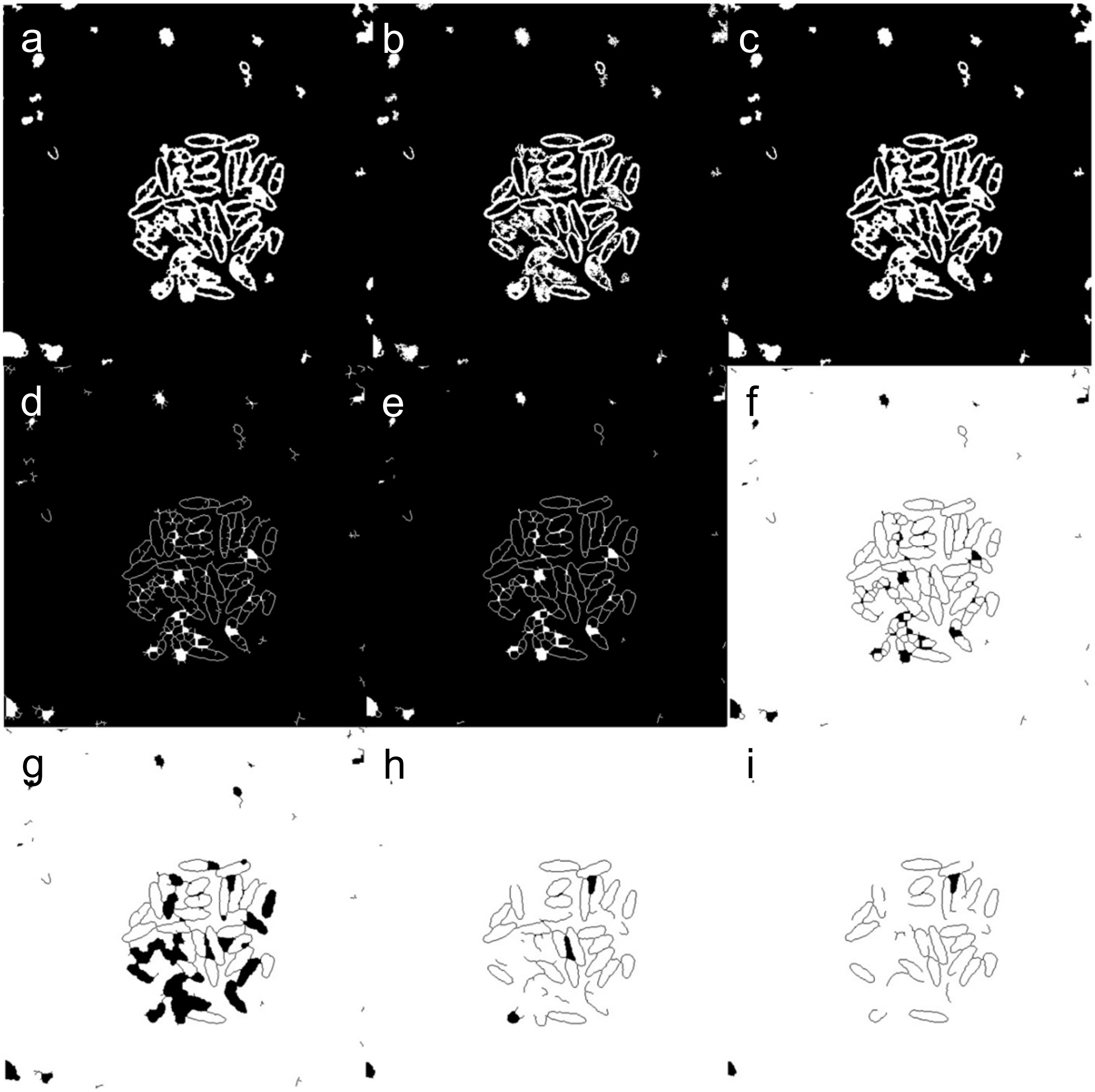

Figure 2A, Clear body workflow for thresholds 1 to 3. Panels "a" through "g" describe the clear body workflow for threshold1 ("Macro: *schisto94_n_1950*"). a) binarization of the flat field corrected image, b) sieve to remove small objects, c) closing to close gaps, d) thinning to reduce outline to a skeleton, e) pruning to prune back branches, f) inversion, g) sieve to retain large objects (this is the threshold1 "Macro: *schisto94_n_1950*" result), h) threshold2 "Macro: *schisto94_n_1800*" result, i) threshold3 "Macro: *schisto94_n_1700*" result.

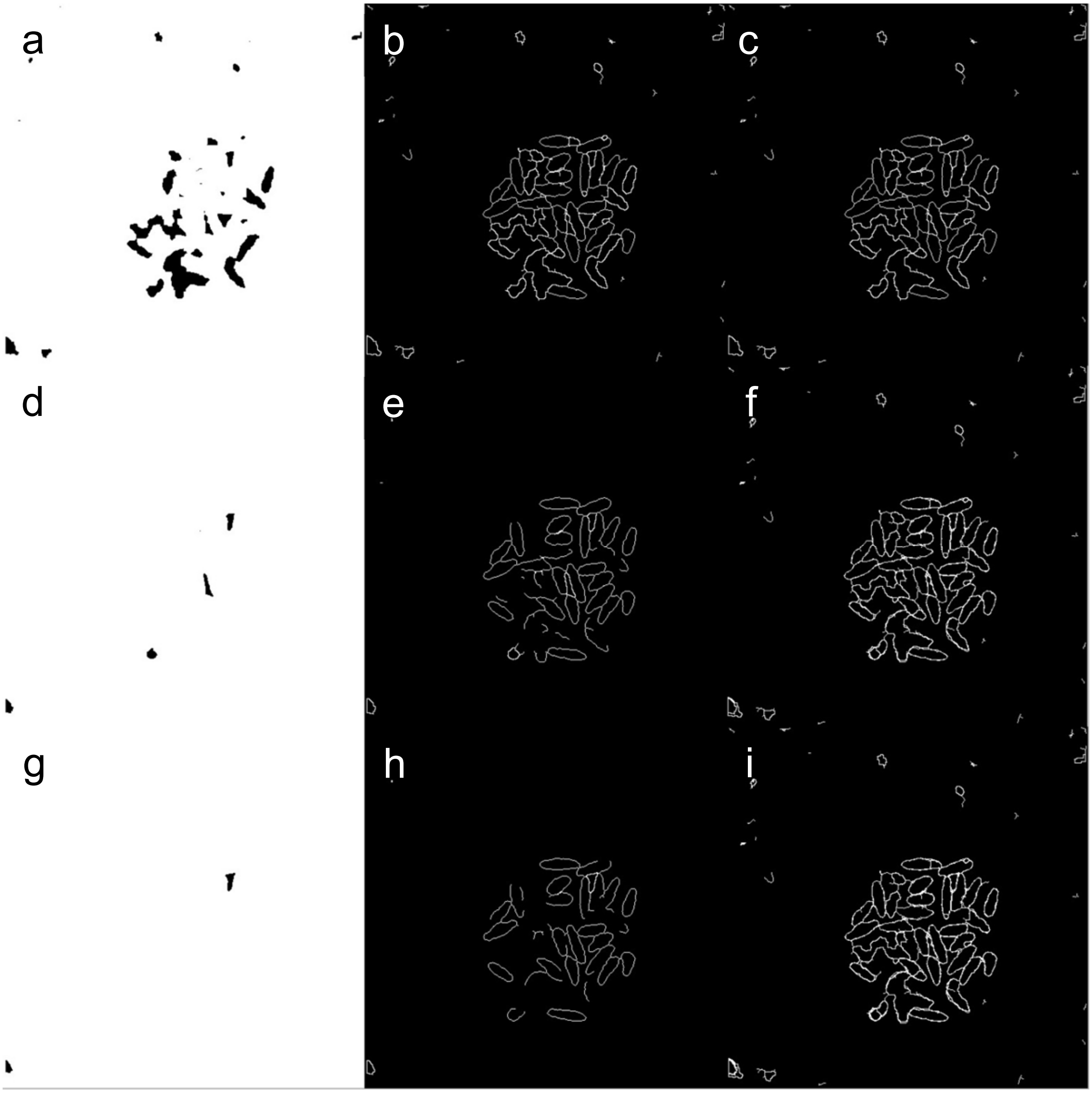

Figure 2B, Merging thresholds 1 to 3 into the fourth target set. Some targets, seen as black blobs in panel “a,d,g”, are discovered through the negative of the clear body workflow. Panel series “a,b,c”, “d,e,f”, and “g,h,i” (continues threshold 1,2,3 respectively) show three steps: 1) dilation of the result to expand the white area and erode the black blobs, 2) subtraction of the result from the dilation which adds black blobs as outlines to our set of outlines, 3) addition of the updated result to the merge channel. As we work through the second and third series, additional data is added to the merge channel until we have a rough merge result in panel “i”.

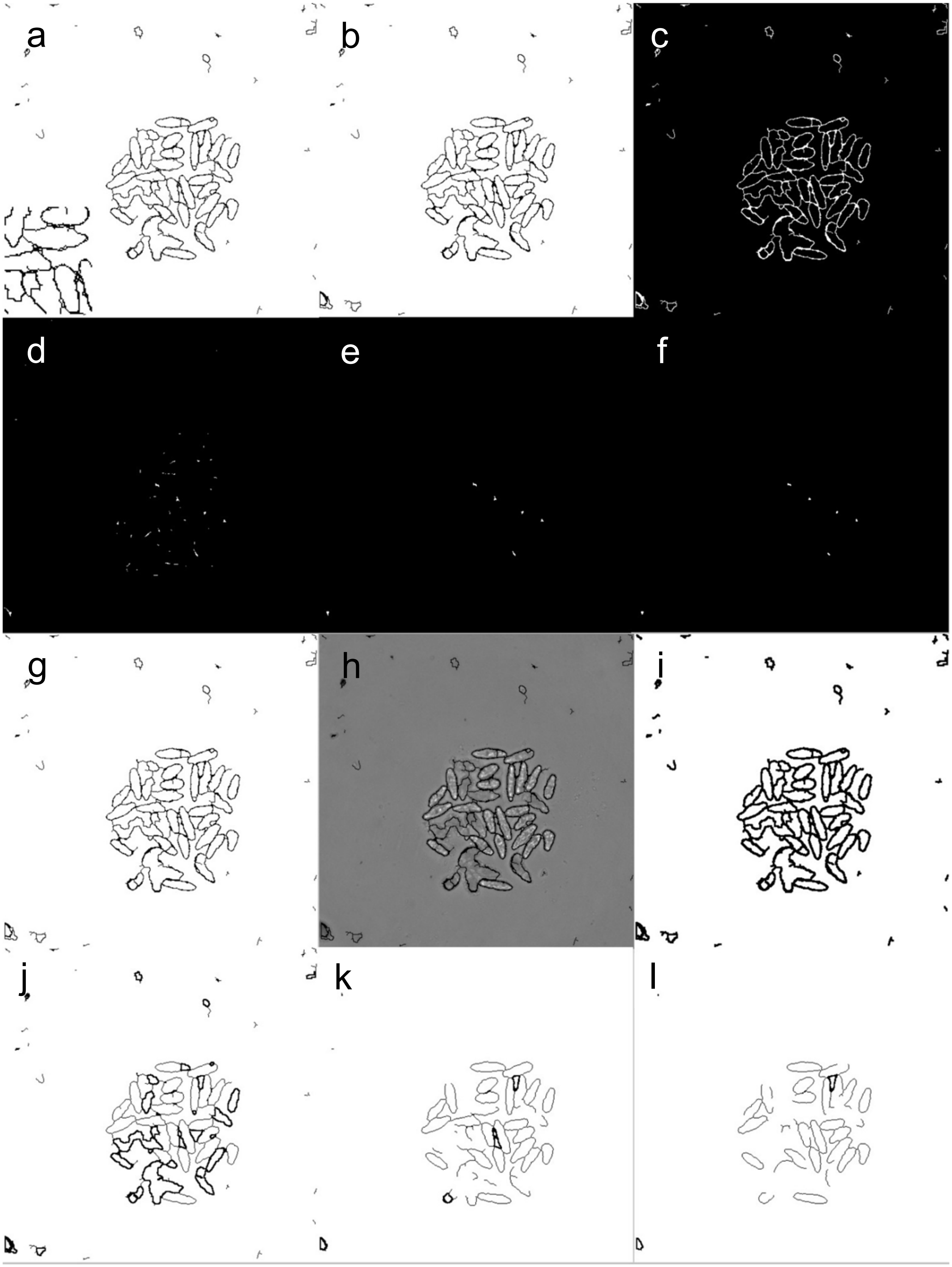
a
b
c
d
e
f
g
h
i
j
k
l

Figure 2C, Refining merge result and preparing for target linking. The merge result has tiny little objects around the perimeter of the large objects which are produced from the intersection of three sets of outlines from thresholds 1 to 3. These small objects are filtered and added back to the image if passed (panel “a” to “g”). The finished merge result in panel “g” is assigned pixel values from the flat-field corrected image (panel “h”). The merge result is eroded to provide objects that can patch the black spaces (a requirement for target linking) in the images for the results for threshold 1 to 3. These operations are shown in panels “i” to “l”. a) inversion of the merge result with a zoomed-in region shown in the left corner to show the tiny little objects, b) sieve to remove small objects, c) inversion, d) erosion e) sieve to remove small objects, f) prune branches, g) add the result from panel “f” to the result from panel “b”, h) assign the pixel values from the flat field corrected image to any non-zero pixels in panel “g”, i) erode the result from panel “g”, j) add the result from “j” to the result from “Fig2A.g”, k) add the result from “j” to the result from “Fig2A.h”, l) add the result from “j” to the result from “Fig2A.i”.

The merge result and results for threshold 1 to 3 are segmented using intensity segmentation with a minimum threshold of 1.00 and a maximum threshold of 4095.00. These settings select all non-zero pixels. The outlines of the objects produced by the preprocessing macro have pixel values of zero. The merge result segmentation has additional post processing steps which use sieve (binary) to retain objects between 1500 µm^2 and 50,000 µm^2 and border object removal to remove any objects within 5 pixels of the image border. Results from threshold 1 to 3 do not use additional post processing steps to remove objects as this could remove objects needed for target linking.

Target linking uses object overlap from different images to determine which objects should be analyzed as a group. Target linking removes objects that do not overlap between all images. Target linking simplifies reporting by putting all feature data for each object found across four images on one row. To make the linking schema easier to read, the four target sets (merge, threshold1, threshold2, threshold3) are named “a”, “b”, “c”, “d”, respectively. Set “a” is linked to set “b”, “c”, and “d” using a “one to one link” to form links “ab”, “ac”, and “ad”. An object in set “a” must be within 75% of the object it is linking to otherwise the link is for those two objects are rejected. Link “ab” and “ac” are joined into link “abc” using a “composed one to one link”. Link “ab” and “ad” are joined into link “abd” using a “composed one to one link”. Link “abc” and “abd” are joined into link “abcd” (which was renamed to “e”) using a “composed one to one link”. (**Figure 2D**)

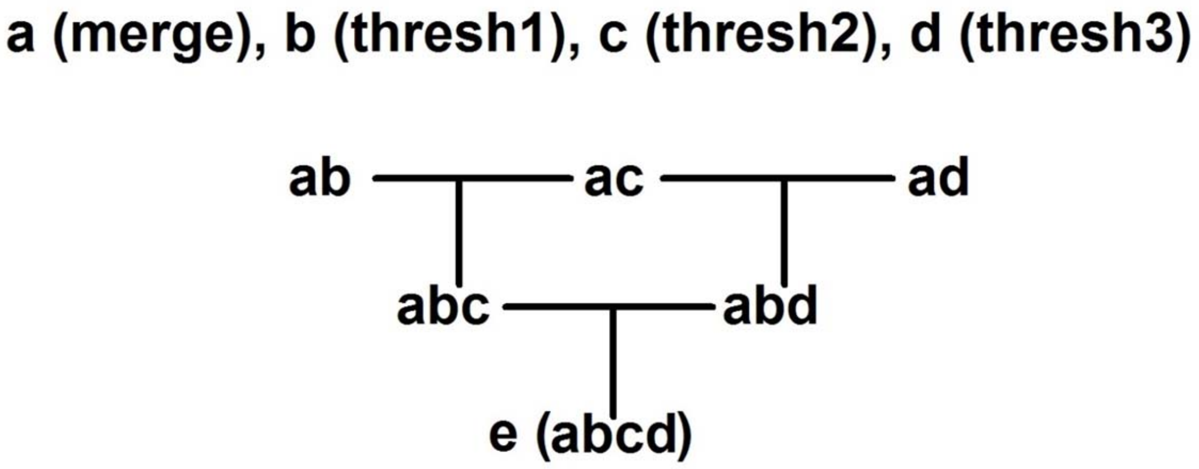

Figure 2D, Target linking schematic. The target sets are represented by letters “a”, “b”, “c”, and “d” with “a” representing the merge data. The merge data contains all possible objects and is the root data that links to “b”, “c”, and “d” forming “ab”, “ac”, and “ad” on the first row. “Composed one to one linking” is used to link “ab” and “ac” using “matching path” data from “a” to form “abc”. “Composed one to one linking” is used to link “ab” and “ad” using “matching path” data from “a” to form “abd”. “Composed one to one linking” is used to link “abc” and “abd” using “matching path” data from “ab” to form “abcd” which is renamed to “e”. Target set “e” contains all the linking data that relates objects found at similar positions across data sets “a”, “b”, “c”, and “d”.

The next macro is called “tegument outliner” because the outlines produced are based on image thresholding which target the tegument of the organism.

3. **Tegument Outliner Preprocessing** *(*Macro: *schisto94_g_master*) (**Figure 3**) (49 operations per image)
    a. Transform FFC image with transform filter = median, (Macro*: schisto94_g_med1_97),* kernel = (do not apply transform)
    b. Transform result from step “3.a” with local arithmetic (Macro*: schisto94_g_med1_97)*:
        i. kernel = 5;
        ii. sel(src<0.97*IAve,4095,0);
    c. Transform result from step “3.a” with transform filter = sieve (binary), retain objects > 1500 µm^2
    d. Transform result from step “3.b” with transform filter = inversion
    e. Transform result from step “3.c” with transform filter = sieve (binary), retain objects > 100 µm^2
    f. Transform result from step “3.b” with transform filter = inversion
    g. Transform result from step “3.c” with transform filter = thinning, passes = 3
    h. Transform result from step “3.d” with transform filter = pruning, passes = 3

i. Repeat steps “3.a” through “3.h” with following setting for step “3.a”, (Macro: *schisto94_g_med3_97*), kernel = 3 **AND** with the following code for step “3.b”, (Macro: *schisto94_g_med3_97*):
    i. kernel = 5;
    ii. sel(src<0.97*IAve,4095,0);

j. Repeat steps “3.a” through “3.h” with following setting for step “3.a”, (Macro: *schisto94_g_med3_99*), kernel = 3 **AND** with the following code for step “3.b”, (Macro: *schisto94_g_med3_99*):
    i. kernel = 5;
    ii. sel(src<0.99*IAve,4095,0);

k. Reset a channel with transform filter = local arithmetic
    i. src = 0;

l. Apply a transform point operation = arithmetic (two src) where source1 = result from “Macro: schisto94_g_med1_97” and source2 = result from step “3.k”. The result is added to the channel used in step “3.k”.
    i. src2+src1;

m. Repeat step “2.l” with source1 = result from “Macro: *schisto94_g_med3_97*”.

n. Repeat step “2.l” with source1 = result from “Macro: *schisto94_g_med3_99*”.

o. Transform merge result (channel in step “k”) with transform filter = inversion

p. Transform result from step “3.o” with transform filter = sieve (binary), retain objects > 100 μm^2

q. Transform result from step “3.p” with transform filter = inversion

r. Transform result from step “3.q” with transform filter = erosion, kernel = 3

s. Transform result from step “3.r” with transform filter = sieve (binary), retain objects > 100 μm^2

t. Transform result from step “3.s” with transform filter = pruning, passes = 3

u. Apply a transform point operation = arithmetic (two src) where source1 = result from step “3.p” and source2 = result from step “3.t”.
    i. src2+src1;

v. Apply a transform point operation = arithmetic (two src) where source1 = result from step “3.u” and source2 = result from step “1.h”.
    i. sel(src1==4095,src2,0);

w. Transform result from “Macro*: schisto94_g_med1_97”* with transform filter = inversion

x. Transform result from “Macro*: schisto94_g_med3_97”* with transform filter = inversion

y. Transform result from “Macro*: schisto94_g_med3_99”* with transform filter = inversion

Steps “3.a” through “3.j” generate the preliminary target data for threshold1 to threshold3. (**Figure 3A**) The refinement of this preliminary data and consolidation into a fourth target begins with step “3.k”. (**Figure 3B**) The fourth target is based on a merge of the data from the three thresholds. The resulting outline will have extra objects produced by the intersections. The extra objects are filtered to reduce complexity of the merged result and this process starts with step “3.o”. (**Figure 3B**) In addition, the merge result represents all possible objects for all tegument outliner target sets. Steps “3.w” through “3.y” invert the results in preparation for segmentation.

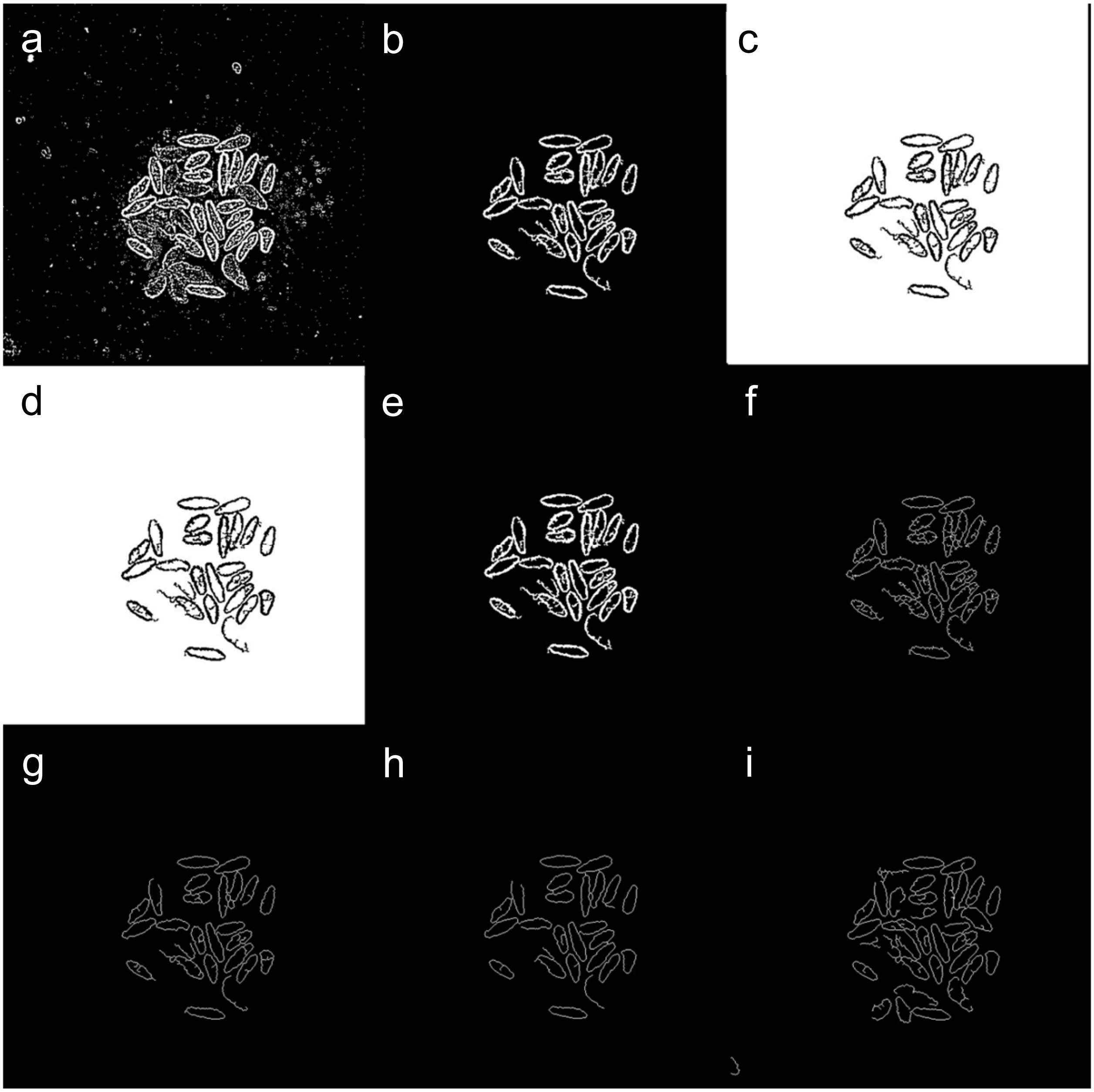

Figure 3A, Tegument outliner workflow for thresholds 1 to 3. Panels "a" through "g" describe the tegument outliner workflow for threshold1 ("Macro*: schisto94_g_med1_97")*. a) binarization of the flat field corrected image, b) sieve to remove small objects, c) inversion, d) sieve to remove small objects, e) inversion, f) thinning to reduce outline to a skeleton, g) pruning to prune back branches (this is the "Macro*: schisto94_g_med1_97"* result*)*, h) threshold2 "Macro*: schisto94_g_med3_97*" result, i) threshold3 "Macro*: schisto94_g_med3_99*" result

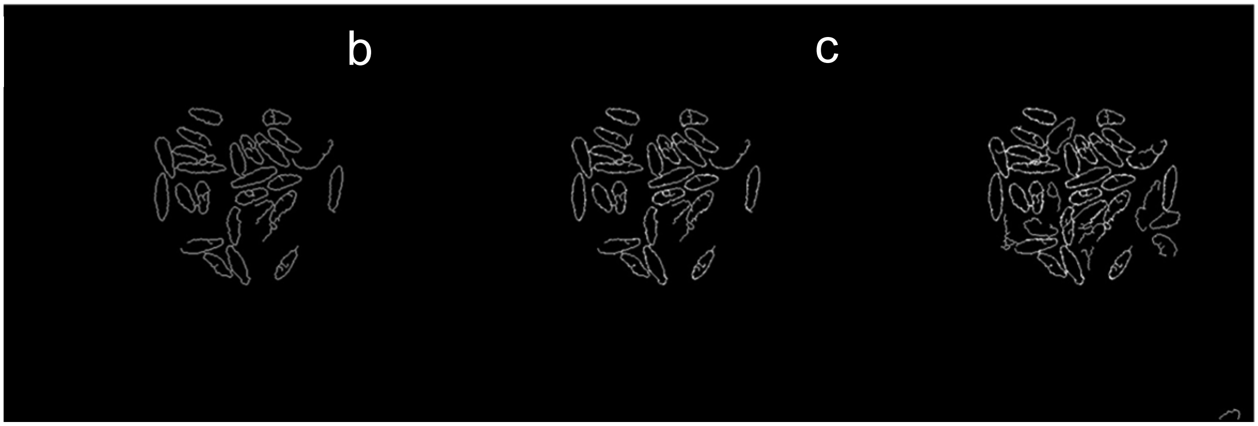

Figure 3B, Merging thresholds 1 to 3 into the fourth target set. The results from each threshold are progressively added together into a rough merge result. a) threshold 1 result, b) threshold 1 and 2 results added together, c) threshold results 1, 2, and 3 added together.

The merge result and results for threshold 1 to 3 are segmented using intensity segmentation with a minimum threshold of 1.00 and a maximum threshold of 4095.00. These settings select all non-zero pixels. The outlines of the objects produced by the preprocessing macro have pixel values of zero. The merge result segmentation has additional post processing steps which use sieve (binary) to retain objects between 1500 µm^2 and 50,000 µm^2 and border object removal to remove any objects within 5 pixels of the image border. Results from threshold 1 to 3 do not use additional post processing steps to remove objects as this could remove objects needed for target linking.

Target linking uses object overlap from different images to determine which objects should be analyzed as a group. Target linking removes objects that do not overlap between all images. Target linking simplifies reporting by putting all feature data for each object found across four images on one row. To make the linking schema easier to read, the four target sets (merge, threshold1, threshold2, threshold3) are named “f”, “g”, “h”, “i”, respectively. Set “f” is linked to set “g”, “h”, and “i” using a “one to one link” to form links “fg”, “fh”, and “fi”. An object in set “f” must be within 75% of the object it is linking to otherwise the link is for those two objects are rejected. Link “fg” and “fh” are joined into link “fgh” using a “composed one to one link”. Link “fg” and “fi” are joined into link “fgi” using a “composed one to one link”. Link “fgh” and “fgi” are joined into link “fghi” (which was renamed to “j”) using a “composed one to one link”. (**Figure 3D**)

479
480

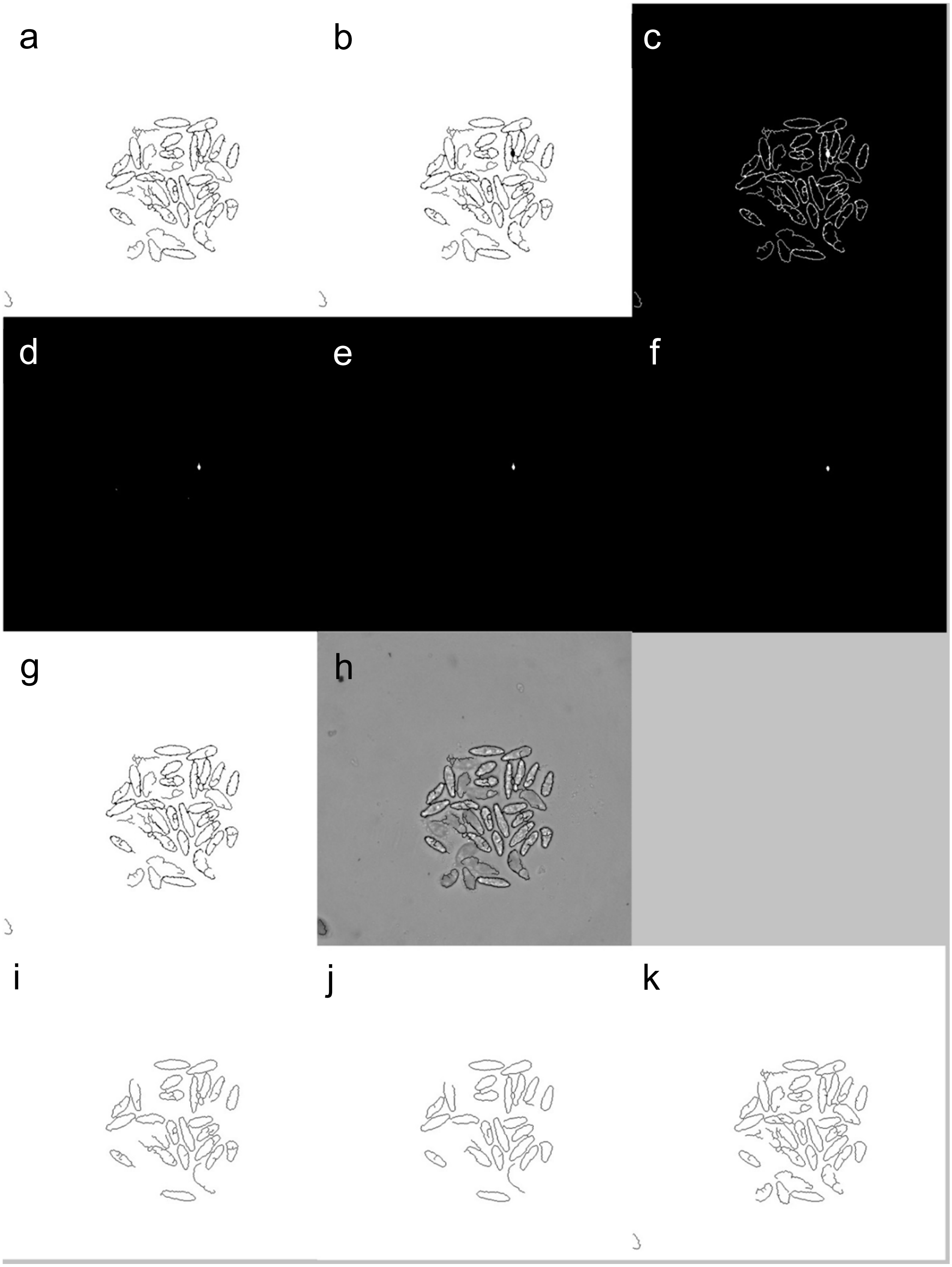

Figure 3C, Refining merge result and preparing for target linking. The merge result has tiny little objects around the perimeter of the large objects which are produced from the intersection of three sets of outlines from thresholds 1 to 3. These small objects are filtered and added back to the image if passed (panel “a” to “g”). The finished merge result in panel “g” is assigned pixel values from the flat-field corrected image (panel “h”). The final results for the three sets of outlines from thresholds 1 to 3 are shown in panels “i” to “k” respectively. a) inversion of the merge result, b) sieve to remove small objects, c) inversion, d) erosion e) sieve to remove small objects, f) prune branches, g) add the result from panel “f” to the result from panel “b”, h) assign the pixel values from the flat field corrected image to any non-zero pixels in panel “g”, i) inversion of “Fig2A.g”, j) inversion of “Fig2A.h”, k) inversion of “Fig2A.i”

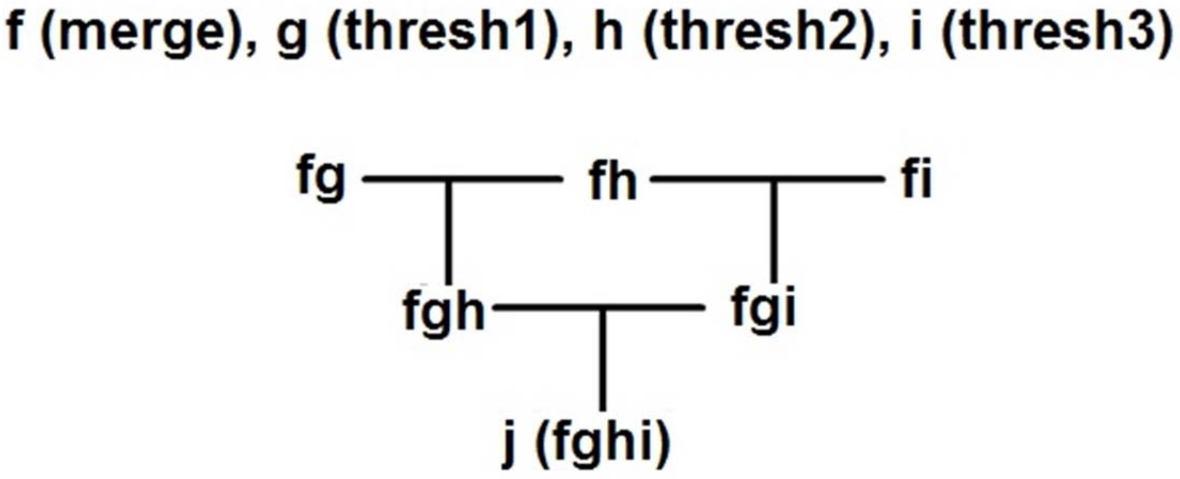

Figure 3D, Target linking schematic. The target sets are represented by letters “f”, “g”, “h”, and “i” with “f” representing the merge data. The merge data contains all possible objects and is the root data that links to “g”, “h”, and “i” forming “fg”, “fh”, and “fi” on the first row. “Composed one to one linking” is used to link “fg” and “fh” using “matching path” data from “f” to form “fgh”. “Composed one to one linking” is used to link “fg” and “fi” using “matching path” data from “f” to form “fgi”. “Composed one to one linking” is used to link “fgh” and “fgi” using “matching path” data from “fg” to form “fghi” which is renamed to “j”. Target set “j” contains all the linking data that relates objects found at similar positions across data sets “f”, “g”, “h”, and “i”.

4. **Dark Body Outliner Preprocessing** *(*Macro: *schisto94_d_master*) (**Figure 4**)
    a. Reset a channel with transform filter = local arithmetic
        i. src = 0; (ch15)
    b. (Macro: *schisto94_d_2050*), Transform FFC image with local arithmetic
        i. sel(src<2050,4095,0)
    c. Transform result from step “4.b” with transform filter = sieve (binary), retain objects > 250 μm^2
    d. Transform result from step “4.c” with transform filter = inversion
    e. Transform result from step “4.d” with local arithmetic

    i. kernel = 3
    ii. sel(src>2*IAve,0,src)

f. Transform result from step “4.e” with transform filter = sieve (binary), retain objects >500 µm^2
g. Transform result from step “4.f” with transform filter = inversion (ch1)
h. Apply a transform point operation = copy result “4.g” (ch13… Macro: *schisto94_d_2050 RESULT*)
i. (Macro: *schisto94_d_fill*), Transform result from step “4.h” with local arithmetic
    i. kernel =7
    ii. sel(src>0,sel(src>IAve,0,4095),0)
j. Transform result from step “4.i” with transform filter = sieve (binary), retain objects > 1500 µm^2
k. Transform result from step “4.j” with transform filter = dilation, kernel = 5 (ch14… Macro: *schisto94_d_fill RESULT*)
l. Transform FFC image with local arithmetic, (Macro*: schisto94_d_1950)*
    i. sel(src<1950,4095,0)
m. Transform result from step “4.l” with transform filter = sieve (binary), retain objects > 250 µm^2
n. Transform result from step “4.m” with transform filter = inversion
o. Transform result from step “4.n” with local arithmetic
    i. kernel = 3
    ii. sel(src>2*IAve,0,src)
p. Transform result from step “4.o” with transform filter = sieve (binary), retain objects > 500 µm^2
q. Transform result from step “4.p” with transform filter = inversion (ch1)
r. Transform result from step “4.q” with local arithmetic, (Macro*: schisto94_c17)*
    i. kernel = 17
    ii. sel(src>0,sel(src==IAve,4095,0),0)
s. Transform result from step “4.r” with transform filter = dilation, kernel = 3
t. Transform result from step “4.s” with transform filter = sieve (binary), retain objects < 2500 µm^2 (see step “4.r” for reason, kernel “seeds” will be small)
u. Apply a transform point operation = arithmetic (two src) where source1 = result from “Macro: *schisto94_c17*” and source2 = result from step “4.a”. The result is added to the channel used in step “4.a”. (ch15)
    i. src2+src1;
v. Repeat step “4.r” through “4.v” with kernel = 15 from “Macro: *schisto94_c15*”.
w. Repeat step “4.r” through “4.v” with kernel = 13 from “Macro: *schisto94_c13*”.
x. Repeat step “4.l” through “4.x” with the following code for step “4.l” (Macro: *schisto94_d_1450*).
    i. sel(src<1450,4095,0);
y. Repeat step “4.l” through “4.x” with the following code for step “4.l” (Macro: *schisto94_d_1150*). After all loops completed this is: (ch15… Merge Seed *RESULT*)
    i. sel(src<1150,4095,0);

z. Apply a transform point operation = arithmetic (two src) where source1 = result from step "3.u" and source2 = result from step "4.k".
    i. sel(src1<1,src1,src2); (ch16… Macro: *schisto94_d_fill RESULT combined with the Tegument Outliner Merge Result*)

aa. Transform result from step "4.aa" with transform filter = sieve (binary), retain objects > 1500 µm^2 (ch16)

bb. Apply a transform point operation = copy result "4.z" (ch1)

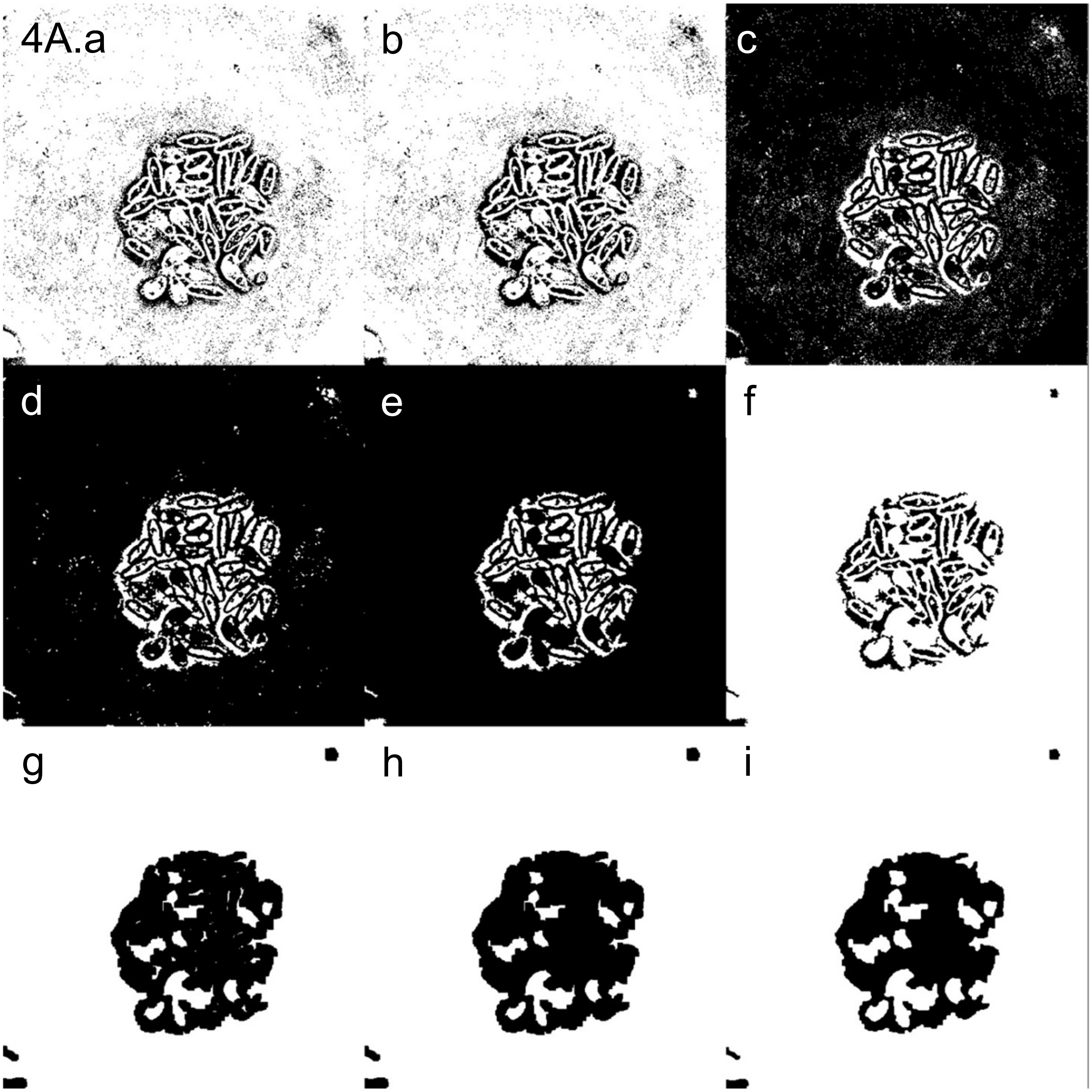

Figure 4A, Dark body outliner part 1. Steps "4.a" through "4.k". a) binarization, b) sieve to remove small objects, c) inversion, d) enhance dark body with local adaptive thresholding, e) sieve to remove small objects, f) inversion to get 1st target image (Macro: schisto94_d_2050), g) local arithmetic to

isolate dark bodies (white blobs), h) sieve to remove small objects, i) dilation to get 2nd target image (Macro: schisto94_d_fill)

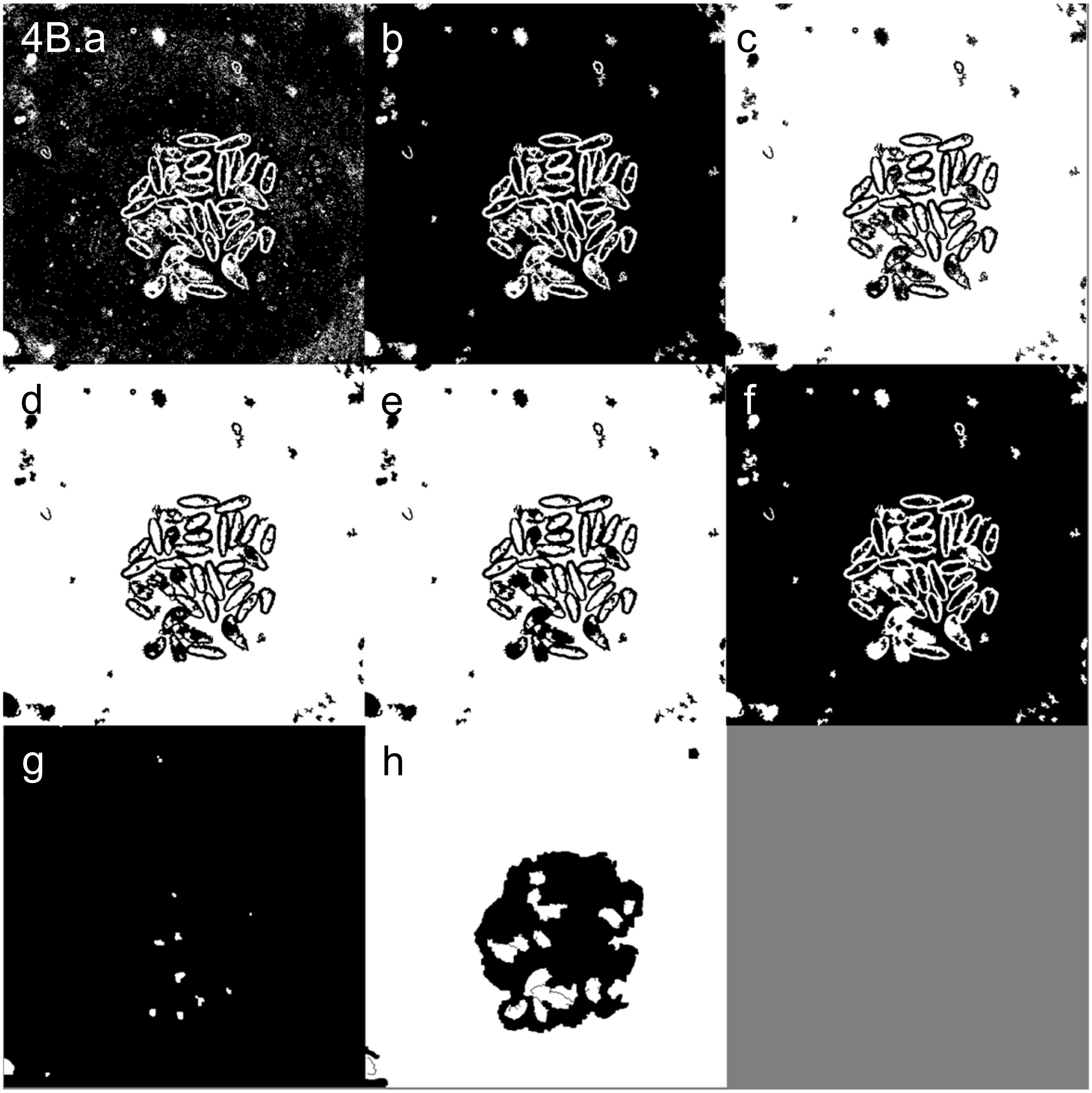

Figure 4B, Dark body outliner part 2. Steps “4.l” through “4.v”. (Macro: *schisto94_d_1950*). a) binarization, b) sieve to remove small objects, c) inversion, d) enhance dark body with local adaptive thresholding, e) sieve to remove small objects, f) inversion, g) dark body “seeds” generated after iterative processing to get 3rd target image (Macro: (see step 4.z)), h) Macro: *schisto94_d_fill* result “4.k” combined with the tegument outliner merge result “3.u” to get the 4th target set (4B.h).

To detect dark bodies a variety of approaches were used. The image was scanned with varying thresholds (density = 1950, 1450, 1150) and kernels (k = 13, 15, and 17) to generate “seeds” of possible dark bodies. The “seed” target set (4B.g) is similar to previously discussed merge results for clear body and tegument outliner in that available target across multiple treatments are represented by a single object. Dark bodies are found (4A.f) by simply taking the inverse of previously described clear body outlining methods. Rather than produce an outline, as with a clear body, dark bodies produce large filled objects with the complication that these objects are sometimes surrounded by other outlines. The surrounding outlines can be diminished to leave the larger dark bodies behind, but at a cost of some distortion to the dark body shape (4A.i). Finally, the tegument outliner merge result (3C.g) is combined with the result from Macro: schisto94_d_fill (4A.h) to separate touching objects (4B.h).

The four binarized dark body target images are transformed back to original FFC image values with the following transform. Apply a transform point operation = arithmetic (two src) where source1 = binarized target image and source2 = FFC image. Code = “sel(src1==4095,src2,0);”.

Dark body “seed” segmentation (K)

The “seed” objects (4B.g) are segmented with intensity segmentation where objects with a pixel value > 0 are masked. The masks are post-processed with a sieve which removes objects less than 250 μm^2.

Dark body “fill” segmentation part I (L)

The objects from the Macro: *schisto94_d_fill* result (4.k) are segmented with intensity segmentation where objects with a pixel value > 0 are masked. Objects within 5 pixels from the border are removed using “border object removal”. The remaining masks are further post-processed with clump breaking using masks segmented in the *dark body “seed” segmentation* as seeds. The masks are post-processed with a sieve which removes objects less greater than 50,000 μm^2.

Dark body “combo” segmentation (seed generation)

The target image generated from Macro: *schisto94_d_fill* result “4.k” combined with the tegument outliner merge result “3.u” are segmented with intensity segmentation where objects with a pixel value > 0 are masked. The masks are post-processed with an erosion (kernel = 3) and a sieve which removes objects less greater than 500 μm^2.

Dark body “fill” segmentation part II (N)

The objects from the Macro: *schisto94_d_fill* result (4.k) are segmented with intensity segmentation where objects with a pixel value > 0 are masked. Objects within 5 pixels from the border are removed using “border object removal”. Clump breaking is applied to the remaining masks with masks segmented in the *dark body “combo” segmentation* as seeds. The masks are further post-processed with watershed clump breaking and a sieve which removes objects less greater than 2,000 μm^2.

Dark body "fill" segmentation part III (O)

The objects from the Macro: *schisto94_d_fill* result (4.k) are segmented with intensity segmentation where objects with a pixel value > 0 are masked. Objects within 5 pixels from the border are removed using "border object removal". The masks are further post-processed with watershed clump breaking and a sieve which removes objects less greater than 2,000 μm^2.

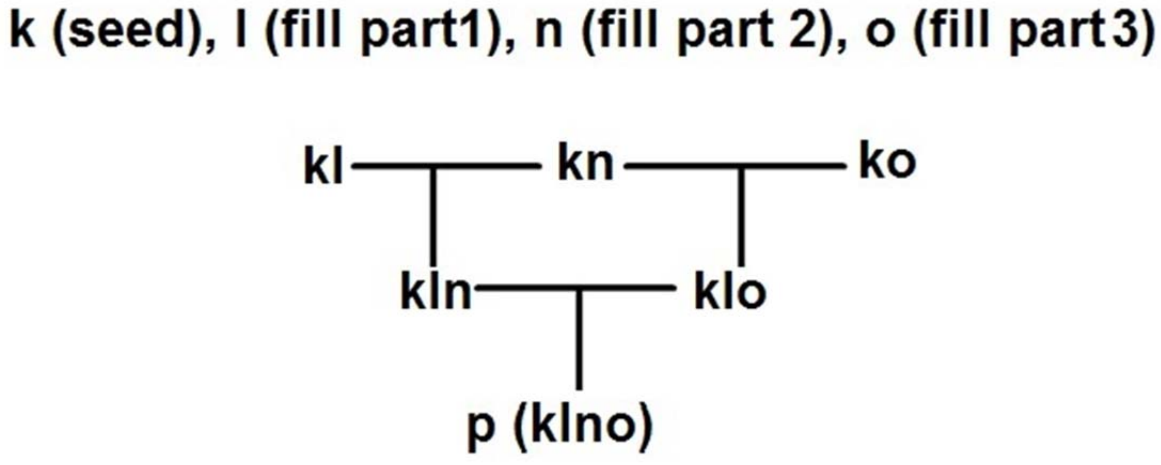

Figure 4D, Target linking schematic. The target sets are represented by letters "k", "l", "n", and "o" with "k" representing the seed data. The merge data contains all possible objects and is the root data that links to "l", "n", and "o" forming "kl", "kn", and "ko" on the first row. "Composed one to one linking" is used to link "kl" and "kn" using "matching path" data from "k" to form "kln". "Composed one to one linking" is used to link "kl" and "ko" using "matching path" data from "k" to form "klo". "Composed one to one linking" is used to link "kln" and "klo" using "matching path" data from "kl" to form "klno" which is renamed to "p". Target set "p" contains all the linking data that relates objects found at similar positions across data sets "k", "l", "n", and "o".

Figure 5A shows various masking results among the 12 target sets generated. It appears that "**Fig5A.a**" captured most of the objects using a clear body merge set. Missing, incomplete, joined, or broken masks have more or less complete counterparts found in other panels. In total, the 12 sets of masks form a more complete set of objects that any one set can provide alone.

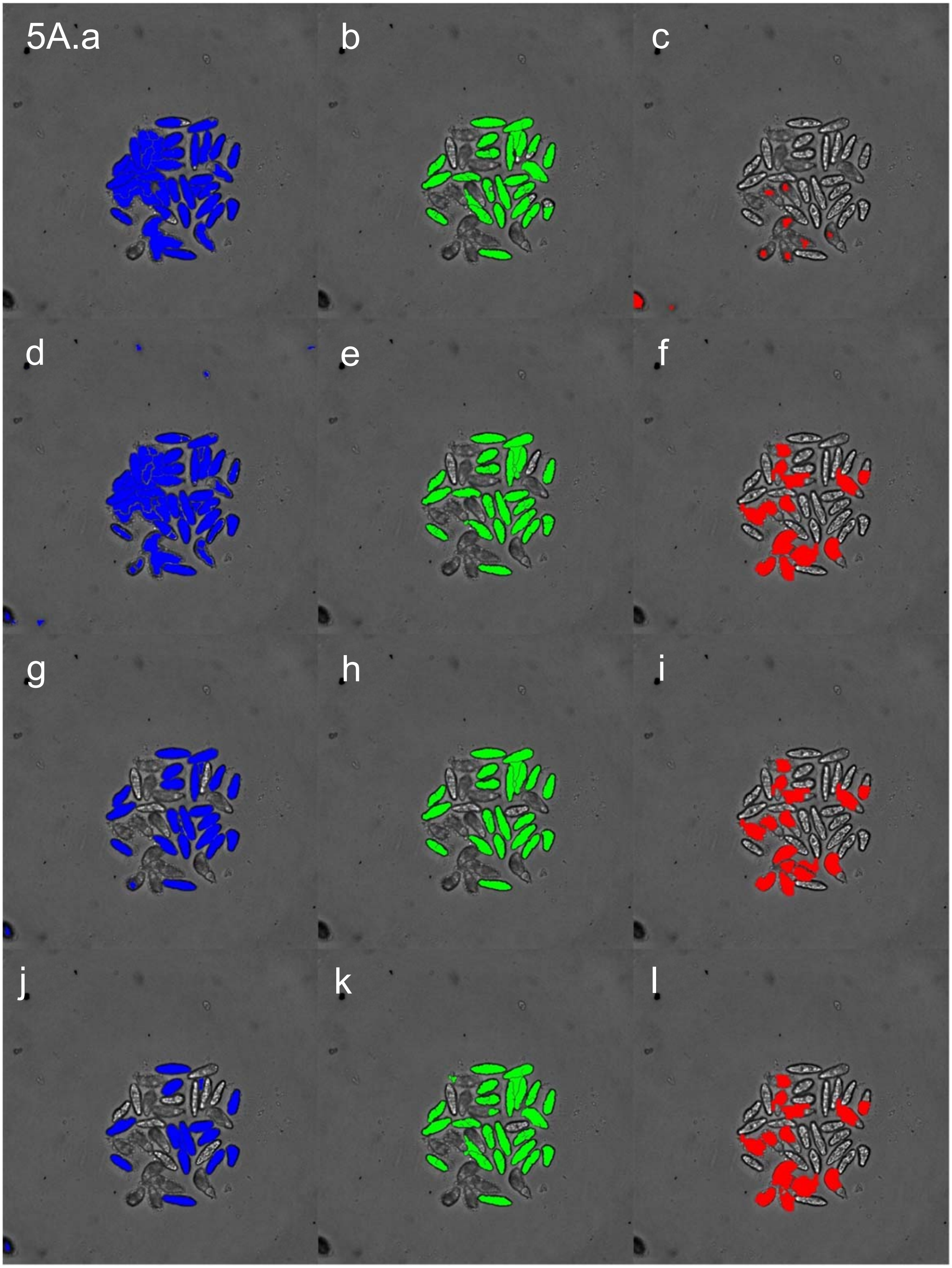

646

Figure 5A, Target set masks for a mixed clear and dark body example. Clear body targets are shown in blue, tegument outliner targets are shown in green and dark body targets in red. The top row of the figure show results from the merge data sets for clear body and tegument outliner with the "seeds" from dark body shown in red. In subsequent rows, the binarization threshold becomes darker for the blue clear body target set, increase in smoothing and decrease in stringency for outline detection in green tegument outliner, and different clump breaking approaches using different seeds shown in the red dark body target set. For the clear body set: a) merge set, d) threshold 1, g) threshold 2, j) threshold 3. For the tegument outline set: b) merge set, e) threshold 1, h) threshold 2, k) threshold 3. For the dark body target set: c) "seeds", f) fill part 1, i) fill part 2, l) fill part 3.

Figure 5B shows various masking results among the 12 target sets generated. It appears that the clear body workflow captured most of the objects using a clear body merge set but most of the masks are joined to other masks leading to a poor segmentation result. The dark body workflow performs better but still suffers from objects that are masked together. The tegument outliner performed the best in terms of finding individual objects with accurate masks. Missing, incomplete, joined, or broken masks have more or less complete counterparts found in other panels. In total, the 12 sets of masks form a more complete set of objects that any one set can provide alone.

Figure 5C, Target set masks for dark body. Clear body targets are shown in blue, tegument outliner targets are shown in green and dark body targets in red. The top row of the figure show results from the merge data sets for clear body and tegument outliner with the "seeds" from dark body shown in red. In subsequent rows, the binarization threshold becomes darker for the blue clear body target set, increase in smoothing and decrease in stringency for outline detection in green tegument outliner, and different clump breaking approaches using different seeds shown in the red dark body target set. For the clear body set: a) merge set, d) threshold 1, g) threshold 2, j) threshold 3. For the tegument outline set: b) merge set, e) threshold 1, h) threshold 2, k) threshold 3. For the dark body target set: c) "seeds", f) fill part 1, i) fill part 2, l) fill part 3.

Figure 5C shows various masking results among the 12 target sets generated. It appears that the clear body workflow captured most of the objects using a clear body merge set but most of the masks are joined to other masks leading to a poor segmentation result. The tegument outliner workflow performs better but still suffers from objects that are masked together or missing altogether. The dark body workflow performed the best in terms of finding individual objects with accurate masks. Missing, incomplete, joined, or broken masks have more or less complete counterparts found in other panels. In total, the 12 sets of masks form a more complete set of objects that any one set can provide alone.

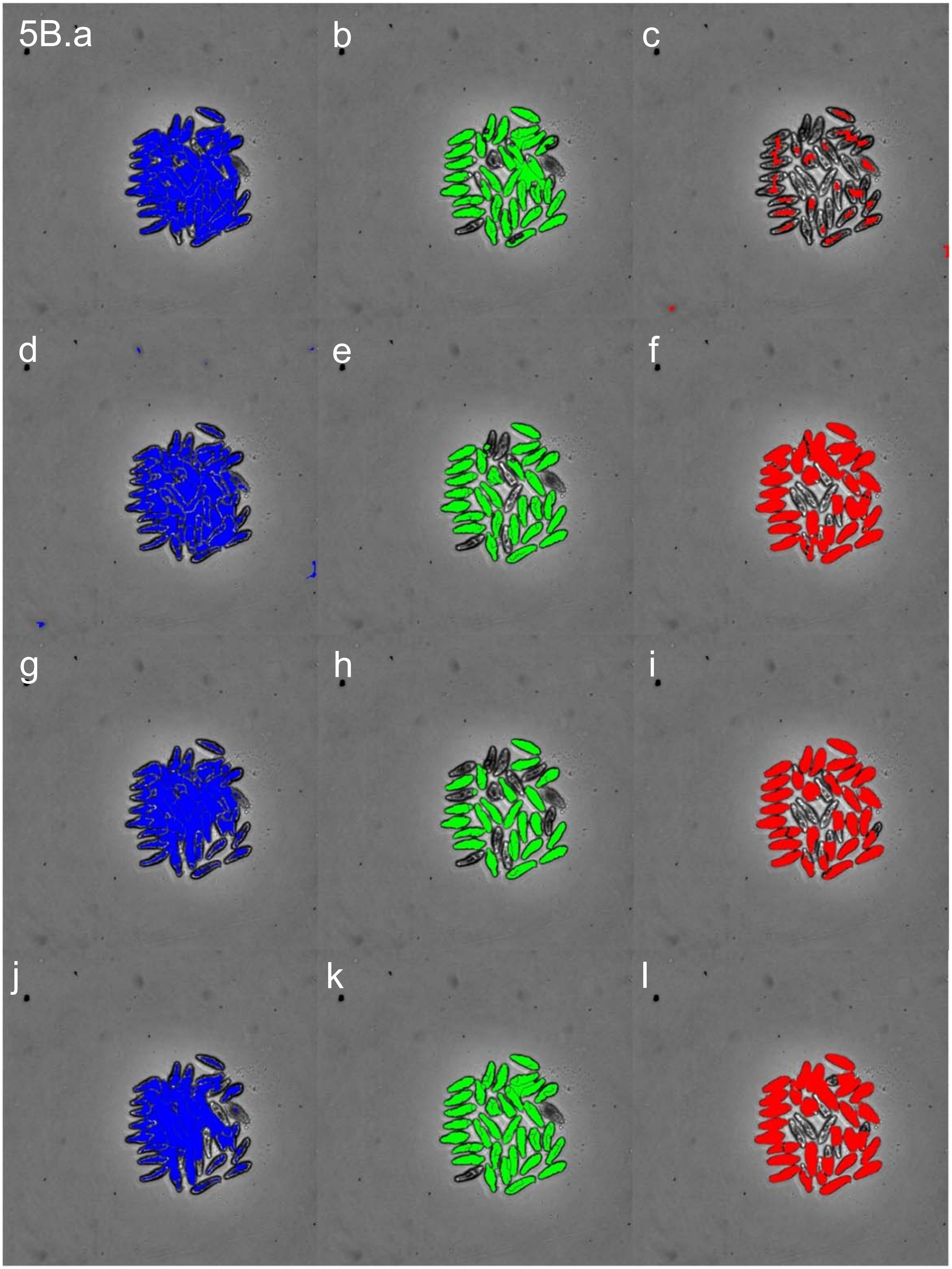

688

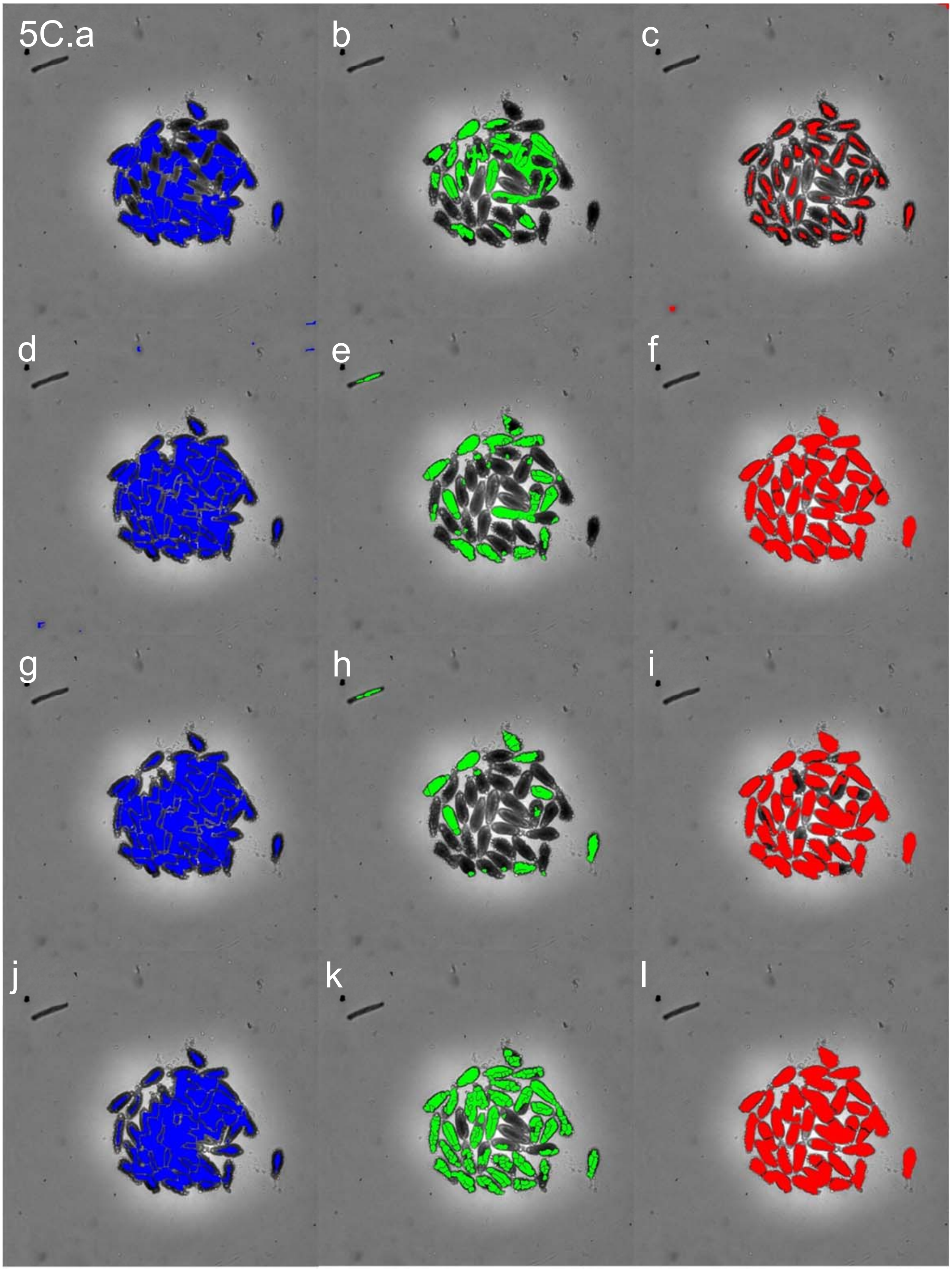
5C.a
b
c
d
e
f
g
h
i
j
k
l

Features (area, length, color, etc) are recorded for every object in all 12 target sets with an IN Cell Developer analysis time of about 5 hours per plate. The data is exported to a comma separated file (CSV) typically weighing in at approximately100 megabytes and containing more than 40 million data points to describe 200,000 objects. Macro parameters can be easily modified to extend the range or resolution of this approach, or even to adapt segmentation to different organisms.

Data Pre-Processing

Before the data from IN Cell Developer can be analyzed and turned into descriptors for database entry, the large data file requires treatment for the following:

1. Correct for segmentation feature offset (bias introduced from differences in segmentation)
2. Choose the best mask out of the twelve possible masks to represent the object. There may be no best object if all fail to be within certain size limits.
3. Use organism-level and well-level data in both appearance and motion-based descriptors to classify objects as either “clear body”, “dark body”, or non-organism objects. Non-organism objects are removed from further analysis.

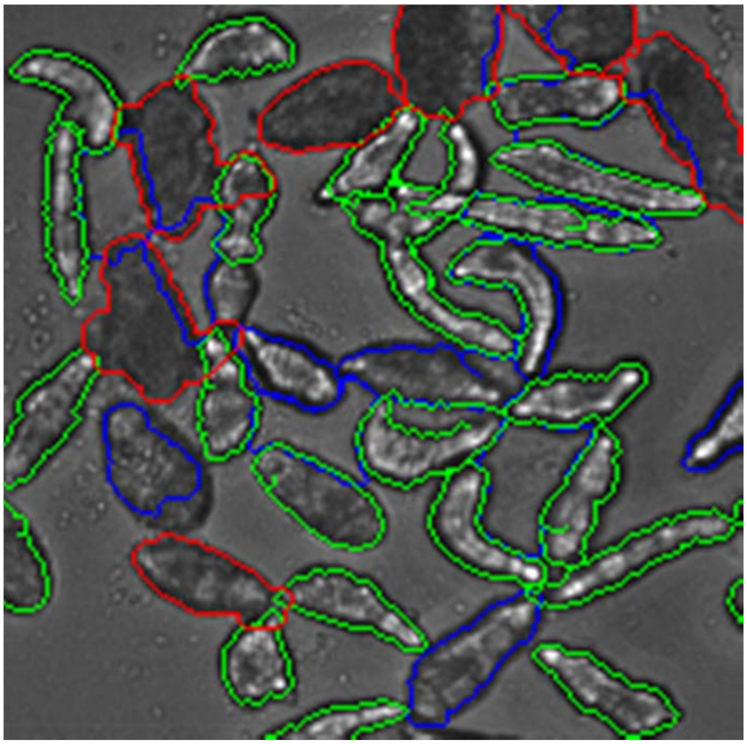

Figure 6 Best mask to represent the object. Image shows the best mask to represent the object from the set of 12 possible masks. Blue outlines are derived from the clear body workflow, green outlines are from the tegument outliner, and red outlines are from the dark body workflow.

Choosing the Best Mask

In the process of choosing the best mask, the objects are first filtered and reorganized. Objects that are too small or too large are removed from analysis. The next step is to link objects within each workflow by x and y position over time. Objects are linked to the coordinates of the last time-point or to an average of all the previous time-points when there are gaps. The workflow that generated masks with the most persistence over time was selected as the best series of masks to move forward with analysis. In the case where workflows generated series with the same amount of persistence, the series with the lower x,y variability over time was selected.

Object Classification

The object classifier uses organism-level data and well-level data to classify objects as clear body, dark body, intra-body artifact, or inter-body artifact. First, the objects were linked through the time-lapse images by x,y position. The time-linked objects were then used to generate feature descriptors (such as mean, standard deviation) at both levels. Descriptors were then entered into Model_1 to classify an object as a clear object or a dark object. Each additional level of classification refines a part of the previous result. Model_2_Clear then classifies a clear object as a clear organism or a clear non-organism (e.g. an object formed between organisms). Model_3_Clear then classifies a clear organism as a complete clear organism or a partial clear organism (e.g. a partial masking of the organism due to an internal boundary). Model_2_Dark and Model_3_Dark perform the same operations but for the dark objects from Model_1.

1. Set objects in the first available time point as reference positions.
2. Set objects in the next available time points at test positions.
3. If a test position is less than 35 μm^2 from a reference position than record a time linkage.
4. Set test positions as reference positions.
5. Repeat steps 2 through 4 until all time points have been processed.
6. Calculate mean and standard deviation at the organism level and at the well level.
7. Calculate the persistence at the organism level and at the well level. Persistence is the count of time-linkages divided by the total number of time-lapse points for a given object. Objects with < 5% persistence are removed from analysis.
8. Classify an object as a clear object or a dark object (model_1: clear < 0.62)
    a. Classify a clear object as a clear organism or a clear non-organism object (model_2_clear: 0.75 < non-organism < 2.5)
        i. Classify a clear organism as a complete clear organism or a partial clear organism (model_3_clear: 0.48 < partial < 2.5)
    b. Classify a dark object as a dark organism or a dark non-organism object (model_2_dark: 0.8 < non-organism < 2.5)
        i. Classify a dark organism as a complete dark organism or a partial dark organism (model_3_dark: 0.54 < partial < 2.5)
9. Write data for clear and dark complete organisms to a new CSV file.

Model_1

n(0) = percent persistence (organism level); n(1) = density – levels mean (organism level); n(2) = SD – levels mean (organism level); n(3) = form factor mean (well level); n(4) = density – levels mean (well level)

R1 = 18.25203487215 + 1.161068183069 * n(0) + 0.75542095675251 * n(1) + -0.68305180301445 * n(2) + -1781.6761055724 * n(3) + -0.045006141550507 * n(4) + 3.2365931882399E-04 * n(0) * n(1) + -4.1740004117079E-04 * n(0) * n(2) + -4.6310458346469 * n(0) * n(3) + -5.7890475949427E-04 * n(0) * n(4) + -9.8420513221299E-05 * n(1) * n(2) + -2.1884015714641 * n(1) * n(3) + -2.7848992087403E-04 * n(1) * n(4)

R2 = 1.3369398769791 * n(2) * n(3) + 8.9582262126079E-04 * n(2) * n(4) + 3.3329037457293 * n(3) * n(4) + -1.8232199977951E-03 * n(0) ^ 2 + -9.4790505473488E-05 * n(1) ^ 2 + -2.2142492356085E-04 * n(2) ^ 2 + 2134.9935522591 * n(3) ^ 2 + -4.7102296313054E-04 * n(4) ^ 2 + 1.2266056885224E-06 * n(0) * n(1) * n(2) + -9.2354913779362E-04 * n(0) * n(1) * n(3) + -1.6491057509021E-07 * n(0) * n(1) * n(4) + 1.5522216710331E-04 * n(0) * n(2) * n(3) + -8.2092941369132E-07 * n(0) * n(2) * n(4)

R3 = 2.9992856931552E-03 * n(0) * n(3) * n(4) + -3.5217569712729E-04 * n(1) * n(2) * n(3) + 6.5107500960996E-08 * n(1) * n(2) * n(4) + 2.6791800633611E-04 * n(1) * n(3) * n(4) + -9.0380771936169E-04 * n(2) * n(3) * n(4) + 3.1247731735274E-06 * n(0) ^ 2 * n(2) + 5.6240376267275E-03 * n(0) ^ 2 * n(3) + -1.4549471230916E-06 * n(0) ^ 2 * n(4) + -7.2417177105256E-08 * n(0) * n(1) ^ 2 + -6.1772369143247E-07 * n(0) * n(2) ^ 2 + 3.781429940739 * n(0) * n(3) ^ 2 + 1.0418245071802E-07 * n(0) * n(4) ^ 2 + 1.0219248176663E-07 * n(1) ^ 2 * n(2)

R4 = 4.5564375868527E-04 * n(1) ^ 2 * n(3) + -3.2106682445134E-08 * n(1) ^ 2 * n(4) + -1.8650850919366E-07 * n(1) * n(2) ^ 2 + 1.9206238869076 * n(1) * n(3) ^ 2 + 1.1738445506832E-07 * n(1) * n(4) ^ 2 + 3.7442327867001E-05 * n(2) ^ 2 * n(3) + 3.1503030609951E-07 * n(2) ^ 2 * n(4) + -0.24033752947827 * n(2) * n(3) ^ 2 + -4.3786761514222E-07 * n(2) * n(4) ^ 2 + -3.4671132225482 * n(3) ^ 2 * n(4) + -6.8418530427196E-04 * n(3) * n(4) ^ 2 + 7.6446830499591E-06 * n(0) ^ 3 + -1.0108280682996E-08 * n(1) ^ 3

R5 = 1.2530435647321E-07 * n(2) ^ 3 + -682.50789300831 * n(3) ^ 3 + 2.3776592427786E-07 * n(4) ^ 3 + -8.3535696931061E-07 * n(0) * n(1) * n(2) * n(3) + -1.5257479853204E-10 * n(0) * n(1) * n(2) * n(4) + 4.0724584996769E-07 * n(0) * n(1) * n(3) * n(4) + 4.604799340958E-07 * n(0) * n(2) * n(3) * n(4) + -8.7746281304306E-10 * n(0) ^ 2 * n(2) ^ 2 + -3.3302009301847E-06 * n(0) ^ 2 * n(2) * n(3) + -4.756929644064E-10 * n(0) ^ 2 * n(2) * n(4) + -3.4228762882309E-03 * n(0) ^ 2 * n(3) ^ 2 + 6.5899847343375E-10 * n(0) ^ 2 * n(4) ^ 2 + -1.1829905701319E-10 * n(0) * n(1) ^ 2 * n(2)

R6 = -6.5159315821518E-08 * n(0) * n(1) ^ 2 * n(3) + 5.2720741951882E-04 * n(0) * n(1) * n(3) ^ 2 + 4.9534714604476E-07 * n(0) * n(2) ^ 2 * n(3) + 4.1269226932606E-10 * n(0) * n(2) ^ 2 * n(4) + 6.3407631003489E-04 * n(0) * n(2) * n(3) ^ 2 + 1.494970625121E-10 * n(0) * n(2) * n(4) ^ 2 + -1.6907505821672E-03 * n(0) * n(3) ^ 2 * n(4) + -5.1365605396076E-07 * n(0) * n(3) * n(4) ^ 2 + -3.6155421452363E-11 * n(1) ^ 2 * n(2) ^ 2 + -4.8126433937627E-08 * n(1) ^ 2 * n(2) * n(3) + -1.7625953726279E-11 * n(1) ^ 2 * n(2) * n(4) + -3.2351132584444E-04 * n(1) ^ 2 * n(3) ^ 2 + -7.9530874313422E-08 * n(1) ^ 2 * n(3) * n(4)

R7 = -2.669952144001E-11 * n(1) ^ 2 * n(4) ^ 2 + 1.4503329430718E-07 * n(1) * n(2) ^ 2 * n(3) + 1.4365500750249E-10 * n(1) * n(2) ^ 2 * n(4) + 4.2874540494959E-04 * n(1) * n(2) * n(3) ^ 2 + -3.6294914163709E-11 * n(1) * n(2) * n(4) ^ 2 + -3.3805927443037E-04 * n(2) ^ 2 * n(3) ^ 2 + -1.4210910495358E-10 * n(2) ^ 2 * n(4) ^ 2 + 2.3003285709267E-07 * n(2) * n(3) * n(4) ^ 2 + 4.9777029628825E-04 * n(3) ^ 2 * n(4) ^ 2 + 2.97760253571E-09 * n(0) ^ 3 * n(2) + -4.3123259790407E-09 * n(0) ^ 3 * n(4) + 2.8293207850737E-11 * n(0) * n(1) ^ 3 + -2.6346389605596E-10 * n(0) * n(2) ^ 3

R8 = -0.67260141510363 * n(0) * n(3) ^ 3 + 1.8752589917427E-08 * n(1) ^ 3 * n(3) + 3.5136792540332E-11 * n(1) ^ 3 * n(4) + -1.7714456049114E-11 * n(1) * n(2) ^ 3 + -0.46479901154684 * n(1) * n(3) ^ 3 + -3.4875673740552E-08 * n(2) ^ 3 * n(3) + -2.5841449832186E-11 * n(2) ^ 3 * n(4) + -0.22134015982785 * n(2) * n(3) ^ 3 + 8.7853677440858E-11 * n(2) * n(4) ^ 3 + 0.86618600900044 * n(3) ^ 3 * n(4) + -9.5374757135764E-12 * n(1) ^ 4 + -3.6583886794751E-11 * n(4) ^ 4 + 0

Model_1 = R1 + R2 + R3 + R4 + R5 + R6 + R7 + R8

Model_2_Clear

n(0) = percent persistence (organism level); n(1) = density – levels mean (organism level); n(2) = SD – levels mean (well level); n(3) = density – levels mean (well level) ; n(4) = pinch mean (organism level) ; n(5) = model_1 (organism level) ; n(6) = area mean (organism level)

R1 = 3050.53971 + -15.56494 * n(0) + -0.83465 * n(1) + -4.70861 * n(2) + -1.83172 * n(3) + -1087.22384 * n(4) + -1798.96904 * n(5) + 0.00388693 * n(6) + 0.00701913 * n(0) * n(1) + 0.00621377 * n(0) * n(2) + 0.012155 * n(0) * n(3) + 1.18447 * n(0) * n(4) + 3.5422 * n(0) * n(5) + 0.000836635 * n(0) * n(6)

R2 = 0.00167653 * n(1) * n(2) + 0.000382736 * n(1) * n(3) + 0.82585 * n(1) * n(4) + 0.48752 * n(1) * n(5) + -0.0000204636 * n(1) * n(6) + 0.00409758 * n(2) * n(3) + 0.22639 * n(2) * n(4) + 0.50064 * n(2) * n(5) + 0.00000930964 * n(2) * n(6) + -0.2123 * n(3) * n(4) + 1.34517 * n(3) * n(5) + -0.000049354 * n(3) * n(6) + 467.08521 * n(4) * n(5) + 0.065277 * n(4) * n(6)

R3 = 0.022834 * n(5) * n(6) + -0.032424 * n(0) ^ 2 + -0.00016082 * n(1) ^ 2 + 0.000283988 * n(2) ^ 2 + 0.0000706305 * n(3) ^ 2 + 665.10179 * n(4) ^ 2 + 297.72877 * n(5) ^ 2 + -0.00000288682 * n(6) ^ 2 + 0.000000576592 * n(0) * n(1) * n(2) + -0.00000550441 * n(0) * n(1) * n(3) + -0.00344384 * n(0) * n(1) * n(4) + -0.000418887 * n(0) * n(1) * n(5) + -0.00000000266297 * n(0) * n(1) * n(6) + -0.00000537496 * n(0) * n(2) * n(3)

R4 = 0.00123966 * n(0) * n(2) * n(4) + -0.000539946 * n(0) * n(2) * n(5) + -0.000000269514 * n(0) * n(2) * n(6) + 0.000685879 * n(0) * n(3) * n(4) + -0.00295768 * n(0) * n(3) * n(5) + -0.000000600777 * n(0) * n(3) * n(6) + 0.45772 * n(0) * n(4) * n(5) + -0.0000773276 * n(0) * n(4) * n(6) + -0.0000149499 * n(0) * n(5) * n(6) + -0.00000121635 * n(1) * n(2) * n(3) + 0.0000187735 * n(1) * n(2) * n(4) + -0.000393188 * n(1) * n(2) * n(5) + -0.00000000836334 * n(1) * n(2) * n(6) + 0.000237552 * n(1) * n(3) * n(4)

R5 = -0.000170248 * n(1) * n(3) * n(5) + 0.0000000424461 * n(1) * n(3) * n(6) + -0.13473 * n(1) * n(4) * n(5) + -0.0000398496 * n(1) * n(4) * n(6) + -0.00000609553 * n(1) * n(5) * n(6) + -0.000155192 * n(2) *

n(3) * n(4) + -0.000135105 * n(2) * n(3) * n(5) + -0.00000000770149 * n(2) * n(3) * n(6) + 0.12141 * n(2) * n(4) * n(5) + 0.00000677018 * n(2) * n(4) * n(6) + -0.0000221139 * n(2) * n(5) * n(6) + -0.17437 * n(3) * n(4) * n(5) + -0.0000342233 * n(3) * n(4) * n(6) + 0.00000269916 * n(3) * n(5) * n(6)

R6 = -0.014 * n(4) * n(5) * n(6) + -0.00000661836 * n(0) ^ 2 * n(1) + -0.00000267154 * n(0) ^ 2 * n(2) + 0.0000291939 * n(0) ^ 2 * n(3) + 0.00558697 * n(0) ^ 2 * n(4) + 0.00128265 * n(0) ^ 2 * n(5) + 0.00000138321 * n(0) ^ 2 * n(6) + 0.00000042153 * n(0) * n(1) ^ 2 + -0.000001289 * n(0) * n(2) ^ 2 + -0.00000234632 * n(0) * n(3) ^ 2 + 1.26238 * n(0) * n(4) ^ 2 + -0.17282 * n(0) * n(5) ^ 2 + -0.0000000408076 * n(0) * n(6) ^ 2 + -0.000000130828 * n(1) ^ 2 * n(2)

R7 = 0.000000125061 * n(1) ^ 2 * n(3) + -0.000289823 * n(1) ^ 2 * n(4) + -0.000023126 * n(1) ^ 2 * n(5) + -0.0000001232 * n(1) * n(2) ^ 2 + -0.000000121779 * n(1) * n(3) ^ 2 + -0.23081 * n(1) * n(4) ^ 2 + -0.16611 * n(1) * n(5) ^ 2 + 0.000000158732 * n(2) ^ 2 * n(3) + -0.0000495972 * n(2) ^ 2 * n(4) + 0.000141303 * n(2) ^ 2 * n(5) + 0.0000000097208 * n(2) ^ 2 * n(6) + -0.00000110647 * n(2) * n(3) ^ 2 + -0.10298 * n(2) * n(4) ^ 2 + 0.026119 * n(2) * n(5) ^ 2

R8 = 0.00000000227912 * n(2) * n(6) ^ 2 + 0.000183837 * n(3) ^ 2 * n(4) + -0.000396325 * n(3) ^ 2 * n(5) + 0.0000000245561 * n(3) ^ 2 * n(6) + -0.31387 * n(3) * n(4) ^ 2 + -0.033724 * n(3) * n(5) ^ 2 + 0.000000000433223 * n(3) * n(6) ^ 2 + -126.01624 * n(4) ^ 2 * n(5) + -0.00723282 * n(4) ^ 2 * n(6) + -88.82786 * n(4) * n(5) ^ 2 + 0.00000545089 * n(4) * n(6) ^ 2 + -0.012727 * n(5) ^ 2 * n(6) + -0.000000923552 * n(5) * n(6) ^ 2 + 0.000027338 * n(0) ^ 3

R9 = 0.0000000615183 * n(1) ^ 3 + -0.00000021971 * n(2) ^ 3 + 0.000000176908 * n(3) ^ 3 + -60.56525 * n(4) ^ 3 + -13.42882 * n(5) ^ 3 + 0.000000000125105 * n(6) ^ 3 + 8.83587E-11 * n(0) * n(1) * n(2) * n(6) + 0.00000163142 * n(0) * n(1) * n(3) * n(4) + 0.000000287771 * n(0) * n(1) * n(3) * n(5) + -0.000188377 * n(0) * n(1) * n(4) * n(5) + -0.000000867302 * n(0) * n(2) * n(3) * n(4) + 0.000000000132956 * n(0) * n(2) * n(3) * n(6) + 0.000514007 * n(0) * n(2) * n(4) * n(5) + -0.0000000938365 * n(0) * n(2) * n(4) * n(6)

R10 = -0.000256585 * n(0) * n(3) * n(4) * n(5) + 0.000000144641 * n(0) * n(3) * n(4) * n(6) + 0.00000017782 * n(1) * n(2) * n(3) * n(5) + -0.000033291 * n(1) * n(2) * n(4) * n(5) + 0.0000000921168 * n(1) * n(2) * n(5) * n(6) + -0.0000684562 * n(1) * n(3) * n(4) * n(5) + 0.0000000189874 * n(1) * n(3) * n(4) * n(6) + -0.00013601 * n(2) * n(3) * n(4) * n(5) + -0.00000000626452 * n(2) * n(3) * n(5) * n(6) + 0.0000154375 * n(2) * n(4) * n(5) * n(6) + 0.0000000022209 * n(0) ^ 2 * n(1) ^ 2 + -0.00000000030416 * n(0) ^ 2 * n(1) * n(6) + -0.00000000197509 * n(0) ^ 2 * n(2) ^ 2 + 0.00000301416 * n(0) ^ 2 * n(2) * n(4)

R11 = -0.00000000426287 * n(0) ^ 2 * n(3) ^ 2 + -0.00000500198 * n(0) ^ 2 * n(3) * n(4) + -0.00000142532 * n(0) ^ 2 * n(3) * n(5) + -0.000000000528475 * n(0) ^ 2 * n(3) * n(6) + 0.00075716 * n(0) ^ 2 * n(4) ^ 2 + 0.00120644 * n(0) ^ 2 * n(4) * n(5) + 0.000000207233 * n(0) ^ 2 * n(4) * n(6) + -0.000000000324208 * n(0) * n(1) ^ 2 * n(2) + -0.000000000244258 * n(0) * n(1) ^ 2 * n(3) + 0.000000000461462 * n(0) * n(1) * n(2) ^ 2 + 0.00000000112886 * n(0) * n(1) * n(3) ^ 2 + 0.000000635517 * n(0) * n(2) ^ 2 * n(4) + -0.000000000036482 * n(0) * n(2) ^ 2 * n(6) + 0.000000001308 * n(0) * n(2) * n(3) ^ 2

R12 = -0.000206579 * n(0) * n(2) * n(4) ^ 2 + -1.17651E-11 * n(0) * n(2) * n(6) ^ 2 + -0.000000833799 * n(0) * n(3) ^ 2 * n(4) + 0.000000636733 * n(0) * n(3) ^ 2 * n(5) + 0.000000000061738 * n(0) * n(3) ^ 2 * n(6) + 0.0000662669 * n(0) * n(3) * n(5) ^ 2 + 2.40122E-11 * n(0) * n(3) * n(6) ^ 2 + -0.0000818538 * n(0) * n(4) ^ 2 * n(6) + 0.043108 * n(0) * n(4) * n(5) ^ 2 + -0.0000000647759 * n(0) * n(4) * n(6) ^ 2 + 0.00000000165957 * n(0) * n(5) * n(6) ^ 2 + -0.00000000014381 * n(1) ^ 2 * n(2) ^ 2 + -0.000000000318973 * n(1) ^ 2 * n(2) * n(3) + 0.0000000115171 * n(1) ^ 2 * n(2) * n(4)

R13 = 0.000000119935 * n(1) ^ 2 * n(3) * n(4) + -0.000000100809 * n(1) ^ 2 * n(3) * n(5) + 0.0000705524 * n(1) ^ 2 * n(4) * n(5) + 0.00000619885 * n(1) ^ 2 * n(5) ^ 2 + 0.000000000267632 * n(1) * n(2) ^ 2 * n(3) + -0.000000051141 * n(1) * n(2) ^ 2 * n(4) + 0.000000000524427 * n(1) * n(2) * n(3) ^ 2 + -0.000000268552 * n(1) * n(3) ^ 2 * n(4) + 0.000000121555 * n(1) * n(3) ^ 2 * n(5) + -1.48346E-11 * n(1) * n(3) ^ 2 * n(6) + 0.000110143 * n(1) * n(3) * n(4) ^ 2 + 0.0000487937 * n(1) * n(3) * n(5) ^ 2 + 0.021921 * n(1) * n(4) * n(5) ^ 2 + 0.00000346283 * n(1) * n(5) ^ 2 * n(6)

R14 = -0.000000000177114 * n(2) ^ 2 * n(3) ^ 2 + 0.0000000210209 * n(2) ^ 2 * n(3) * n(4) + -0.0000000818784 * n(2) ^ 2 * n(3) * n(5) + 0.0000376644 * n(2) ^ 2 * n(4) * n(5) + -9.31503E-13 * n(2) ^ 2 * n(6) ^ 2 + 0.000106985 * n(2) * n(3) * n(4) ^ 2 + 0.055031 * n(2) * n(4) ^ 2 * n(5) + -0.035459 * n(2) * n(4) * n(5) ^ 2 + 0.0000755324 * n(3) ^ 2 * n(4) * n(5) + -0.0000189566 * n(3) ^ 2 * n(5) ^ 2 + 0.040084 * n(3) * n(4) ^ 2 * n(5) + -0.00000000234695 * n(3) * n(4) * n(6) ^ 2 + 17.20136 * n(4) ^ 2 * n(5) ^ 2 + 0.004504 * n(4) * n(5) ^ 2 * n(6)

R15 = 0.000000561702 * n(4) * n(5) * n(6) ^ 2 + 0.0000000479781 * n(5) ^ 2 * n(6) ^ 2 + -0.00000000944116 * n(0) ^ 3 * n(1) + 0.0000000115184 * n(0) ^ 3 * n(2) + -0.0000000158032 * n(0) ^ 3 * n(3) + 0.00000536878 * n(0) ^ 3 * n(4) + 0.00000362103 * n(0) ^ 3 * n(5) + 0.00000000106752 * n(0) ^ 3 * n(6) + -0.31887 * n(0) * n(4) ^ 3 + -0.00358755 * n(0) * n(5) ^ 3 + 0.000000000155012 * n(1) ^ 3 * n(2) + 0.000000025693 * n(1) ^ 3 * n(5) + 8.47252E-11 * n(1) * n(2) ^ 3 + 0.0000000269769 * n(2) ^ 3 * n(4)

R16 = -0.040793 * n(2) * n(4) ^ 3 + 0.013965 * n(2) * n(5) ^ 3 + 0.0000000679358 * n(3) ^ 3 * n(4) + 0.00494237 * n(4) ^ 3 * n(6) + 0.000439059 * n(5) ^ 3 * n(6) + 3.26886E-11 * n(5) * n(6) ^ 3 + 0.0000000400428 * n(0) ^ 4 + -1.89276E-11 * n(1) ^ 4 + -3.18337E-11 * n(3) ^ 4 + 25.12615 * n(4) ^ 4 + 2.44964 * n(5) ^ 4 + -7.13436E-15 * n(6) ^ 4 + 0 + 0

Model_2_Clear = R1 + R2 + R3 + R4 + R5 + R6 + R7 + R8 + R9 + R10 + R11 + R12 + R13 + R14 + R15 + R16

Model_3_Clear

n(0) = percent persistence (organism level); n(1) = form factor mean (organism level); n(2) = density – levels mean (well level) ; n(3) = model_1 (organism level) ; n(4) = model_2_clear (organism level)

R1 = 32.52402 + -0.54831 * n(0) + 55.81306 * n(1) + -0.029513 * n(2) + 13.34291 * n(3) + 13.05986 * n(4)

R2 = 0.16126 * n(0) * n(1) + 0.000562305 * n(0) * n(2) + 0.11982 * n(0) * n(3) + -0.08123 * n(0) * n(4) + -0.062883 * n(1) * n(2) + 5.31126 * n(1) * n(3)

R3 = -4.81 * n(1) * n(4) + -0.021003 * n(2) * n(3) + -0.00540019 * n(2) * n(4) + -0.70453 * n(3) * n(4) + -0.00140765 * n(0) ^ 2 + 9.04074 * n(1) ^ 2

R4 = 0.00000723861 * n(2) ^ 2 + 0.11459 * n(3) ^ 2 + -1.78504 * n(4) ^ 2 + -0.0000819342 * n(0) * n(1) * n(2) + -0.021891 * n(0) * n(1) * n(3) + 0.013961 * n(0) * n(1) * n(4)

R5 = -0.0000529987 * n(0) * n(2) * n(3) + 0.0000474044 * n(0) * n(2) * n(4) + 0.000387712 * n(0) ^ 2 * n(1) + 0.000000947006 * n(0) ^ 2 * n(2) + 0.0000959058 * n(0) ^ 2 * n(3) + -0.000237815 * n(0) ^ 2 * n(4)

R6 = -0.04276 * n(0) * n(1) ^ 2 + -0.000000154351 * n(0) * n(2) ^ 2 + 0.00112266 * n(0) * n(4) ^ 2 + -2.45185 * n(1) ^ 2 * n(3) + 2.50057 * n(1) ^ 2 * n(4) + 0.0000161108 * n(1) * n(2) ^ 2

R7 = 0.40533 * n(1) * n(4) ^ 2 + 0.00000632871 * n(2) ^ 2 * n(3) + 0.00083054 * n(2) * n(4) ^ 2 + -0.45553 * n(3) ^ 2 * n(4) + 0.87222 * n(3) * n(4) ^ 2 + -0.0000031075 * n(0) ^ 3

R8 = -2.84358 * n(1) ^ 3 + 0.14201 * n(3) ^ 3 + -0.38692 * n(4) ^ 3 + 0 + 0 + 0

Model_3_Clear = R1 + R2 + R3 + R4 + R5 + R6 + R7 + R8

Model_2_Dark

n(0) = density – levels mean (organism level); n(1) = SD – levels mean (organism level); n(2) = percent persistence (well level); n(3) = SD – levels mean (well level); n(4) = pinch mean (organism level); n(5) = model_1 (organism level)

R1 = -361.12162 + 0.56347 * n(0) + 1.91376 * n(1) + -4.00323 * n(2) + -0.013169 * n(3) + -139.20978 * n(4) + 218.65312 * n(5) + -0.00153984 * n(0) * n(1) + 0.0052126 * n(0) * n(2) + -0.000454827 * n(0) * n(3) + -0.050456 * n(0) * n(4) + -0.18053 * n(0) * n(5) + -0.024777 * n(1) * n(2) + -0.00141908 * n(1) * n(3) + -0.12855 * n(1) * n(4) + -0.15031 * n(1) * n(5) + 0.011171 * n(2) * n(3) + 2.32431 * n(2) * n(4) + -2.09687 * n(2) * n(5) + 0.53271 * n(3) * n(4) + -0.027043 * n(3) * n(5)

R2 = 12.99119 * n(4) * n(5) + -0.000309284 * n(0) ^ 2 + -0.000849861 * n(1) ^ 2 + 0.043616 * n(2) ^ 2 + -0.000056373 * n(3) ^ 2 + 124.14887 * n(4) ^ 2 + -56.25437 * n(5) ^ 2 + 0.0000128757 * n(0) * n(1) * n(2) + 0.00000098784 * n(0) * n(1) * n(3) + 0.000182684 * n(0) * n(1) * n(4) + 0.00002946 * n(0) * n(1) * n(5) + -0.00000513663 * n(0) * n(2) * n(3) + -0.000177285 * n(0) * n(2) * n(4) + 0.0000739509 * n(0) * n(2) * n(5) + -0.000231917 * n(0) * n(3) * n(4) + 0.0000143359 * n(0) * n(3) * n(5) + 0.051313 * n(0) * n(4) * n(5) + 0.00000505626 * n(1) * n(2) * n(3) + -0.000114908 * n(1) * n(2) * n(4) + 0.0036413 * n(1) * n(2) * n(5) + -0.000463003 * n(1) * n(3) * n(4)

R3 = 0.000195403 * n(1) * n(3) * n(5) + 0.027341 * n(1) * n(4) * n(5) + -0.0057082 * n(2) * n(3) * n(4) + -0.00151504 * n(2) * n(3) * n(5) + -0.37303 * n(2) * n(4) * n(5) + -0.032101 * n(3) * n(4) * n(5) + 0.000000363092 * n(0) ^ 2 * n(1) + -0.00000126177 * n(0) ^ 2 * n(2) + 0.000000335253 * n(0) ^ 2 * n(3) + 0.00000930206 * n(0) ^ 2 * n(4) + 0.0000630895 * n(0) ^ 2 * n(5) + 0.000000625327 * n(0) * n(1) ^ 2 + -0.0000534651 * n(0) * n(2) ^ 2 + -0.0000000239318 * n(0) * n(3) ^ 2 + -0.00396581 * n(0) * n(4) ^ 2 + 0.023233 * n(0) * n(5) ^ 2 + 0.00000989143 * n(1) ^ 2 * n(2) + 0.00000105728 * n(1) ^ 2 * n(3) + -0.000160825 * n(1) ^ 2 * n(4) + 0.0000106839 * n(1) ^ 2 * n(5) + 0.0000984379 * n(1) * n(2) ^ 2

R4 = 0.000000294665 * n(1) * n(3) ^ 2 + 0.026272 * n(1) * n(4) ^ 2 + -0.021385 * n(1) * n(5) ^ 2 + -0.00000220276 * n(2) ^ 2 * n(3) + 0.00809653 * n(2) ^ 2 * n(4) + 0.018287 * n(2) ^ 2 * n(5) + -0.00000561739 * n(2) * n(3) ^ 2 + -1.65978 * n(2) * n(4) ^ 2 + 0.50979 * n(2) * n(5) ^ 2 + 0.000142765 * n(3) ^ 2 * n(4) + 0.0000247584 * n(3) ^ 2 * n(5) + -0.10679 * n(3) * n(4) ^ 2 + 0.030073 * n(3) * n(5) ^ 2 + -54.71951 * n(4) ^ 2 * n(5) + 5.80519 * n(4) * n(5) ^ 2 + 0.000000067095 * n(0) ^ 3 + -0.000000310388 * n(1) ^ 3 + -0.000236629 * n(2) ^ 3 + 0.000000169864 * n(3) ^ 3 + -6.52428 * n(4) ^ 3 + 5.28688 * n(5) ^ 3

R5 = 0.000000128977 * n(0) * n(1) * n(3) * n(4) + -0.0000000405581 * n(0) * n(1) * n(3) * n(5) + -0.0000552203 * n(0) * n(1) * n(4) * n(5) + 0.000000890618 * n(0) * n(2) * n(3) * n(4) + 0.000000325049 * n(0) * n(2) * n(3) * n(5) + 0.00000169698 * n(1) * n(2) * n(3) * n(4) + -0.000000758555 * n(1) * n(2) * n(3) * n(5) + 0.00057653 * n(2) * n(3) * n(4) * n(5) + -6.57659E-11 * n(0) ^ 2 * n(1) ^ 2 + -0.00000000245433 * n(0) ^ 2 * n(1) * n(2) + -0.00000000022752 * n(0) ^ 2 * n(1) * n(3) + 0.0000000214646 * n(0) ^ 2 * n(1) * n(4) + 0.0000000125412 * n(0) ^ 2 * n(2) ^ 2 + 0.000000000904972 * n(0) ^ 2 * n(2) * n(3) + 4.11355E-11 * n(0) ^ 2 * n(3) ^ 2 + 0.0000000265477 * n(0) ^ 2 * n(3) * n(4) + -0.0000104834 * n(0) ^ 2 * n(4) * n(5) + -0.00000366468 * n(0) ^ 2 * n(5) ^ 2 + -0.00000000218385 * n(0) * n(1) ^ 2 * n(2) + -0.000000000175384 * n(0) * n(1) ^ 2 * n(3) + -0.0000000678748 * n(0) * n(1) ^ 2 * n(4)

R6 = -0.0000000209727 * n(0) * n(1) * n(2) ^ 2 + -0.000000000109807 * n(0) * n(1) * n(3) ^ 2 + -0.0000800513 * n(0) * n(1) * n(4) ^ 2 + 0.0000145427 * n(0) * n(1) * n(5) ^ 2 + -0.00000000641016 * n(0) * n(2) ^ 2 * n(3) + -0.00000476213 * n(0) * n(2) ^ 2 * n(4) + 0.00000000101544 * n(0) * n(2) * n(3) ^ 2 + 0.000304876 * n(0) * n(2) * n(4) ^ 2 + -0.0000663645 * n(0) * n(2) * n(5) ^ 2 + -0.0000000404017 * n(0) * n(3) ^ 2 * n(4) + 0.0000394658 * n(0) * n(3) * n(4) ^ 2 + -0.00000946448 * n(0) * n(3) * n(5) ^ 2 + 0.013504 * n(0) * n(4) ^ 2 * n(5) + -0.00662779 * n(0) * n(4) * n(5) ^ 2 + -0.000000035197 * n(1) ^ 2 * n(2) ^ 2 + -0.0000000101636 * n(1) ^ 2 * n(2) * n(3) + 0.000000190077 * n(1) ^ 2 * n(3) * n(4) + 0.0000734302 * n(1) ^ 2 * n(4) ^ 2 + 0.0000483842 * n(1) ^ 2 * n(4) * n(5) + -0.0000000182885 * n(1) * n(2) ^ 2 * n(3) + -0.0000227993 * n(1) * n(2) ^ 2 * n(5)

R7 = 0.00000000324656 * n(1) * n(2) * n(3) ^ 2 + -0.0000941191 * n(1) * n(2) * n(5) ^ 2 + -0.0000000755622 * n(1) * n(3) ^ 2 * n(5) + 0.010924 * n(1) * n(4) * n(5) ^ 2 + 0.0000000126476 * n(2) ^ 2 * n(3) ^ 2 + 0.0000114672 * n(2) ^ 2 * n(3) * n(4) + 0.00286912 * n(2) ^ 2 * n(4) ^ 2 + -0.00348402 * n(2) ^ 2 * n(4) * n(5) + -0.00275424 * n(2) ^ 2 * n(5) ^ 2 + 0.000000519982 * n(2) * n(3) ^ 2 * n(5) + 0.000450946 * n(2) * n(3) * n(4) ^ 2 + 0.31246 * n(2) * n(4) ^ 2 * n(5) + 3.02457 * n(4) ^ 2 * n(5) ^ 2 + -2.00237E-11 * n(0) ^ 3 * n(1) + -0.000000000070125 * n(0) ^ 3 * n(3) + -0.00000000793741 * n(0) ^ 3 * n(5) + -0.000000000158606 * n(0) * n(1) ^ 3 + 0.0000000785055 * n(0) * n(2) ^ 3 + -9.21427E-11 * n(0) * n(3) ^ 3 + -0.011845 * n(0) * n(4) ^ 3 + 0.00000000297869 * n(1) ^ 3 * n(2)

R8 = -0.000000000243674 * n(1) ^ 3 * n(3) + -0.0000000759836 * n(1) ^ 3 * n(5) + -0.00000000015798 * n(1) * n(3) ^ 3 + 0.031905 * n(1) * n(4) ^ 3 + -0.00308156 * n(1) * n(5) ^ 3 + -0.0000000397407 * n(3) ^ 3 * n(4) + -0.0000000277537 * n(3) ^ 3 * n(5) + -0.0022742 * n(3) * n(5) ^ 3 + -0.72285 * n(4) * n(5) ^ 3 + -3.17195E-12 * n(0) ^ 4 + 0.000000000456645 * n(1) ^ 4 + 0.000000336111 * n(2) ^ 4 + 7.93096E-11 * n(3) ^ 4 + 6.09015 * n(4) ^ 4 + -0.31393 * n(5) ^ 4 + 0 + 0 + 0 + 0 + 0 + 0

Model_2_Dark = R1 + R2 + R3 + R4 + R5 + R6 + R7 + R8

Model_3_Dark

n(0) = percent persistence (organism level); n(1) = x-y position stdev (organism level); n(2) = area mean (organism level); n(3) = area stdev (organism level); n(4) = SD – levels stdev (organism level); n(5) model_1 (organism level); n(6) model_2 (organism level);

R1 = -4.61017 + 0.092707 * n(0) + 0.35653 * n(1) + 0.000750986 * n(2) + -0.00452111 * n(3) + 0.011807 * n(4) + 5.71269 * n(5) + -1.49563 * n(6) + -0.00976856 * n(0) * n(1) + -0.0000263884 * n(0) * n(2) + 0.000135389 * n(0) * n(3) + 0.000208516 * n(0) * n(4) + -0.08961 * n(0) * n(5) + 0.013908 * n(0) * n(6)

R2 = 0.000111107 * n(1) * n(2) + -0.000521866 * n(1) * n(3) + -0.02206 * n(1) * n(4) + -0.39836 * n(1) * n(5) + -0.32854 * n(1) * n(6) + -0.0000000992238 * n(2) * n(3) + 0.0000185838 * n(2) * n(4) + -0.000983153 * n(2) * n(5) + 0.000152417 * n(2) * n(6) + 0.0000626892 * n(3) * n(4) + 0.000877242 * n(3) * n(5) + 0.00418134 * n(3) * n(6) + 0.00787532 * n(4) * n(5) + 0.045654 * n(4) * n(6)

R3 = 0.55274 * n(5) * n(6) + -0.00027944 * n(0) ^ 2 + -0.013183 * n(1) ^ 2 + 0.0000000794713 * n(2) ^ 2 + 0.00000436954 * n(3) ^ 2 + -0.000737969 * n(4) ^ 2 + -1.60087 * n(5) ^ 2 + 1.28554 * n(6) ^ 2 + 0.00000121589 * n(0) * n(1) * n(2) + 0.000000680117 * n(0) * n(1) * n(3) + 0.000063997 * n(0) * n(1) * n(4) + 0.00737386 * n(0) * n(1) * n(5) + 0.00629009 * n(0) * n(1) * n(6) + -0.0000000203623 * n(0) * n(2) * n(3)

R4 = 0.000000283108 * n(0) * n(2) * n(4) + 0.0000214193 * n(0) * n(2) * n(5) + -0.00000950404 * n(0) * n(2) * n(6) + 0.000000257292 * n(0) * n(3) * n(4) + -0.0000845602 * n(0) * n(3) * n(5) + -0.0000444008 * n(0) * n(3) * n(6) + 0.00015789 * n(0) * n(4) * n(5) + -0.000189701 * n(0) * n(4) * n(6) + 0.0000000668764 * n(1) * n(2) * n(3) + -0.00000226437 * n(1) * n(2) * n(4) + -0.0000495035 * n(1) * n(2) * n(5) + -0.0000570377 * n(1) * n(2) * n(6) + -0.0000110973 * n(1) * n(3) * n(4) + -0.0000984269 * n(1) * n(3) * n(5)

R5 = -0.000547909 * n(1) * n(3) * n(6) + 0.000826802 * n(1) * n(4) * n(5) + -0.00174602 * n(1) * n(4) * n(6) + -0.034571 * n(1) * n(5) * n(6) + -0.0000000183872 * n(2) * n(3) * n(4) + 0.00000080816 * n(2) * n(3) * n(5) + 0.0000000300624 * n(2) * n(3) * n(6) + -0.0000272219 * n(2) * n(4) * n(5) + -0.0000091387 * n(2) * n(4) * n(6) + -0.000119087 * n(2) * n(5) * n(6) + 0.0000690982 * n(3) * n(4) * n(5) + 0.0000172096 * n(3) * n(4) * n(6) + -0.000273843 * n(3) * n(5) * n(6) + 0.00861831 * n(4) * n(5) * n(6)

R6 = 0.0000303932 * n(0) ^ 2 * n(1) + 0.000000156693 * n(0) ^ 2 * n(2) + -0.000000170271 * n(0) ^ 2 * n(3) + -0.0000168954 * n(0) ^ 2 * n(4) + 0.000243014 * n(0) ^ 2 * n(5) + -0.0000186158 * n(0) ^ 2 * n(6) + -0.000473015 * n(0) * n(1) ^ 2 + -0.000000000917335 * n(0) * n(2) ^ 2 + 0.0000000098126 * n(0) * n(3) ^ 2 + 0.00000251944 * n(0) * n(4) ^ 2 + 0.017146 * n(0) * n(5) ^ 2 + -0.000841143 * n(0) * n(6) ^ 2 + 0.0000110572 * n(1) ^ 2 * n(2) + 0.0000145286 * n(1) ^ 2 * n(3)

R7 = 0.00205469 * n(1) ^ 2 * n(4) + 0.040184 * n(1) ^ 2 * n(5) + 0.060707 * n(1) ^ 2 * n(6) + -0.0000000343855 * n(1) * n(2) ^ 2 + 0.000000215682 * n(1) * n(3) ^ 2 + 0.000447475 * n(1) * n(4) ^ 2 + 0.241 * n(1) * n(5) ^ 2 + 0.28481 * n(1) * n(6) ^ 2 + 0.000000000152796 * n(2) ^ 2 * n(3) + -0.000000000242684 * n(2) ^ 2 * n(4) + 0.00000000363359 * n(2) ^ 2 * n(5) + 0.00000012185 * n(2) ^ 2 * n(6) + -0.000000000959079 * n(2) * n(3) ^ 2 + 0.00000018851 * n(2) * n(4) ^ 2

R8 = 0.000173728 * n(2) * n(5) ^ 2 + -0.000284603 * n(2) * n(6) ^ 2 + 0.0000000102328 * n(3) ^ 2 * n(4) + -0.0000022087 * n(3) ^ 2 * n(5) + -0.0000000934567 * n(3) ^ 2 * n(6) + -0.00000105153 * n(3) * n(4) ^ 2 + -0.0000934181 * n(3) * n(5) ^ 2 + -0.00295776 * n(3) * n(6) ^ 2 + 0.000423315 * n(4) ^ 2 * n(5) + -0.0000799979 * n(4) ^ 2 * n(6) + -0.00450556 * n(4) * n(5) ^ 2 + -0.02169 * n(4) * n(6) ^ 2 + 0.26604 * n(5) * n(6) ^ 2 + -0.00000126558 * n(0) ^ 3

R9 = -0.0039262 * n(1) ^ 3 + -4.15924E-12 * n(2) ^ 3 + -5.47209E-11 * n(3) ^ 3 + -0.0000191162 * n(4) ^ 3 + 0.059443 * n(5) ^ 3 + -0.49754 * n(6) ^ 3 + -0.000000000383815 * n(0) * n(1) * n(2) * n(3) + -0.000000750809 * n(0) * n(1) * n(2) * n(5) + -0.00000073033 * n(0) * n(1) * n(2) * n(6) + 0.00000000066314 * n(0) * n(1) * n(3) * n(4) + 0.00000157802 * n(0) * n(1) * n(3) * n(5) + 0.00000032997 * n(0) * n(1) * n(3) * n(6) + 0.0000444528 * n(0) * n(1) * n(4) * n(5) + 0.0000138956 * n(0) * n(1) * n(4) * n(6)

R10 = 7.76253E-11 * n(0) * n(2) * n(3) * n(4) + 0.00000000959345 * n(0) * n(2) * n(3) * n(5) + 0.00000000542978 * n(0) * n(2) * n(3) * n(6) + -0.000000138988 * n(0) * n(2) * n(4) * n(5) + -0.000000255355 * n(0) * n(3) * n(4) * n(5) + 0.000000000366051 * n(1) * n(2) * n(3) * n(4) + -0.0000000198672 * n(1) * n(2) * n(3) * n(5) + 0.0000000271726 * n(1) * n(2) * n(3) * n(6) + 0.000000965152 * n(1) * n(2) * n(4) * n(5) + 0.00000090072 * n(1) * n(2) * n(4) * n(6) + 0.00000812757 * n(1) * n(3) * n(4) * n(5) + 0.00000797685 * n(1) * n(3) * n(4) * n(6) + 0.000103226 * n(1) * n(3) * n(5) * n(6) + -0.0000207396 * n(3) * n(4) * n(5) * n(6)

R11 = -0.00000195856 * n(0) ^ 2 * n(1) ^ 2 + 0.0000000269254 * n(0) ^ 2 * n(1) * n(3) + -0.0000508801 * n(0) ^ 2 * n(1) * n(5) + 2.49496E-11 * n(0) ^ 2 * n(2) * n(3) + -0.0000000742936 * n(0) ^ 2 * n(2) * n(5) + 0.0000000306992 * n(0) ^ 2 * n(2) * n(6) + -0.000000000135004 * n(0) ^ 2 * n(3) ^ 2 + -0.00000000292662 * n(0) ^ 2 * n(3) * n(4) + 0.000000278337 * n(0) ^ 2 * n(3) * n(5) + 0.00000997994 * n(0) ^ 2 * n(4) * n(5) + -0.0000639369 * n(0) ^ 2 * n(6) ^ 2 + 0.0000000558002 * n(0) * n(1) ^ 2 * n(2) + -0.000000143373 * n(0) * n(1) ^ 2 * n(3) + -0.00000414178 * n(0) * n(1) ^ 2 * n(4)

R12 = -0.000000000741061 * n(0) * n(1) * n(3) ^ 2 + -0.00000126359 * n(0) * n(1) * n(4) ^ 2 + -0.00203358 * n(0) * n(1) * n(6) ^ 2 + -2.37659E-13 * n(0) * n(2) ^ 2 * n(3) + 0.00000000033799 * n(0) * n(2) ^ 2 * n(6) + 1.08851E-12 * n(0) * n(2) * n(3) ^ 2 + -0.00000000142941 * n(0) * n(2) * n(4) ^ 2 + -0.00000293071 * n(0) * n(2) * n(5) ^ 2 + 0.00000237668 * n(0) * n(2) * n(6) ^ 2 + -8.93754E-11 * n(0) * n(3) ^ 2 * n(4) + 0.00000000419135 * n(0) * n(3) * n(4) ^ 2 + 0.00000995675 * n(0) * n(3) * n(6) ^ 2 + -0.000271624 * n(0) * n(4) * n(5) ^ 2 + -0.0000000015755 * n(1) ^ 2 * n(2) * n(3)

R13 = -0.0000076574 * n(1) ^ 2 * n(2) * n(5) + -0.00000993414 * n(1) ^ 2 * n(2) * n(6) + -0.00000000914334 * n(1) ^ 2 * n(3) ^ 2 + 0.00000238695 * n(1) ^ 2 * n(3) * n(5) + -0.000688278 * n(1) ^ 2 * n(4) * n(5) + -0.000619047 * n(1) ^ 2 * n(4) * n(6) + -0.018618 * n(1) ^ 2 * n(6) ^ 2 + 0.0000000176265 * n(1) * n(2) ^ 2 * n(5) + 0.0000000156328 * n(1) * n(2) ^ 2 * n(6) + 0.000019277 * n(1) * n(2) * n(6) ^ 2 + -0.00000000110944 * n(1) * n(3) ^ 2 * n(4) + -0.0000000105332 * n(1) * n(3) ^ 2 * n(5) + -0.0000000252387 * n(1) * n(3) ^ 2 * n(6) + -0.0000000702263 * n(1) * n(3) * n(4) ^ 2

R14 = 0.00012233 * n(1) * n(3) * n(6) ^ 2 + -0.000128546 * n(1) * n(4) ^ 2 * n(5) + -0.000106207 * n(1) * n(4) ^ 2 * n(6) + 0.0030741 * n(1) * n(4) * n(6) ^ 2 + -0.087995 * n(1) * n(5) * n(6) ^ 2 + 7.37175E-13 * n(2) ^ 2 * n(3) * n(4) + -9.59323E-11 * n(2) ^ 2 * n(3) * n(5) + -6.02571E-11 * n(2) ^ 2 * n(3) * n(6) + 2.50502E-12 * n(2) * n(3) ^ 2 * n(4) + 0.000000000442336 * n(2) * n(3) ^ 2 * n(5) + 9.93414E-11 * n(2) *

n(3) ^ 2 * n(6) + -6.40403E-11 * n(2) * n(3) * n(4) ^ 2 + -0.000000292517 * n(2) * n(3) * n(5) ^ 2 + 0.00000955825 * n(2) * n(4) * n(5) ^ 2

R15 = 0.000000000350581 * n(3) ^ 2 * n(4) ^ 2 + -0.0000000168097 * n(3) ^ 2 * n(4) * n(5) + -0.0000000161471 * n(3) ^ 2 * n(4) * n(6) + -0.0000000692311 * n(3) ^ 2 * n(5) * n(6) + -0.000020757 * n(3) * n(4) * n(5) ^ 2 + 0.000617916 * n(3) * n(5) * n(6) ^ 2 + 0.00021377 * n(4) ^ 2 * n(5) * n(6) + 0.000000339072 * n(0) ^ 3 * n(1) + -0.00000000207837 * n(0) ^ 3 * n(3) + 0.000019645 * n(0) * n(1) ^ 3 + 4.45966E-14 * n(0) * n(2) ^ 3 + 9.04952E-13 * n(0) * n(3) ^ 3 + 0.0000000763313 * n(0) * n(4) ^ 3 + 0.00283387 * n(0) * n(6) ^ 3

R16 = 0.000000219559 * n(1) ^ 3 * n(2) + 0.000000953974 * n(1) ^ 3 * n(3) + -0.0000133042 * n(1) ^ 3 * n(4) + 0.000882434 * n(1) ^ 3 * n(6) + -0.067623 * n(1) * n(5) ^ 3 + 2.58842E-15 * n(2) ^ 3 * n(3) + -9.82343E-12 * n(2) ^ 3 * n(6) + 0.0000000054835 * n(3) * n(4) ^ 3 + 0.000463001 * n(3) * n(5) ^ 3 + 0.000345934 * n(3) * n(6) ^ 3 + 0.00000320697 * n(4) ^ 3 * n(5) + 0.00000317484 * n(4) ^ 3 * n(6) + -0.2124 * n(5) * n(6) ^ 3 + -7.87648E-17 * n(3) ^ 4

R17 = 0.12069 * n(6) ^ 4

Model_3_Dark = R1 + R2 + R3 + R4 + R5 + R6 + R7 + R8 + R9 + R10 + R11 + R12 + R13 + R14 + R15 + R16 + R17

<u>MySQL Database</u>

The experiment is defined in the database (**Figure 6**) by updating the “project”, “assay”, “version”, “plate”, “well”, “compound”, “lot”, and “session” tables. In the database, “version” is the version of the assay, “lot” is the version of the compound, and “session” stores the location of data files (data CSV, image acquisition XDCE), the date of the start of the iteration, and the status of the data processing. Next, the timestamp of every image is parsed and loaded into the “time” table. Then the file from data pre-processing is imported into the “raw” table and linked to the “time” table. The “time” table becomes the route to travel to different points in time within the same well whereas “session” is useful in separating the campaign into experimental iterations. The “time” table also stores flags indicating a well should not be considered for analysis. From the “raw” table, the data is processed and loaded into “frag”, “worm”, “result”, and “effect”.

Data in the “raw” table were reorganized into “fragments”. Organisms in each time frame were linked to organisms in subsequent time frames using the method described in “Object Classification” steps 1 through 5. There is a chance that a link cannot be found or a link will be found a later time point. These gaps in the linking operation produce fragments or varying size. If the fragments are too small (< 4 time points) the fragments are not used for analysis. Four or more time points per fragment ensures 3 or more data points to estimate rate which is the mean amount of change per time frame.

Long enough fragments in the “fragment” table were reorganized into “worms”. The “static” mean and standard deviation of each feature for all worm time points were calculated. The “rate” mean and standard deviation of each feature’s absolute change between time points were calculated. The “frequency” of each feature is determined by measuring the number of directional changes per time. Changes in value that are below the system noise were carried over if the change continues in the same direction. (**Table 2**).

The worms in the “worm” table are analyzed at the well level to provide results to the “results” table. The mean and standard deviation for “static”, “rate”, and frequency modes were calculated.

Results in the “statistic” table are used to calculate effect sizes and Mahalanobis Distance in the “effects” table and “Mahalanobis” table. The Glass effect size is used to calculate effect sizes. The Mahalanobis Distance is calculated for the “static”, “rate” and “frequency” categories separately, the combination of “static” and “rate” categories, and the full combination of “static”, “rate”, and “frequency” categories. Degeneracy data is located in the “degeneracy” table. Degeneracy is the number of dark worms divided by the total worms per well.

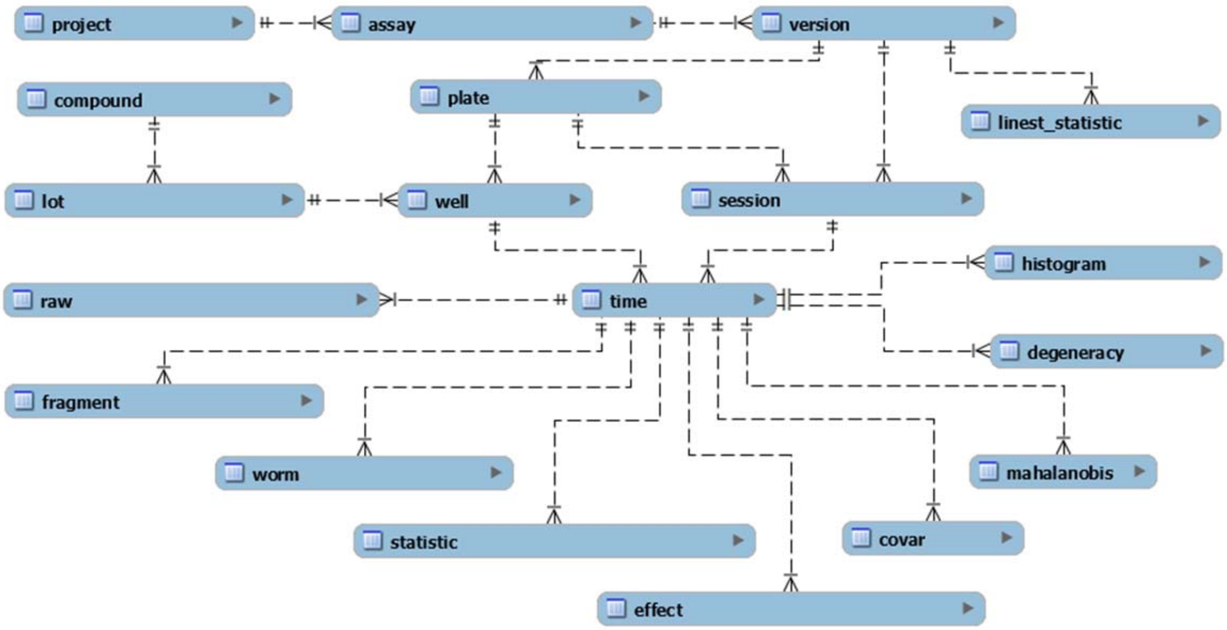

Figure 6 MySQL database. A model of the database is shown with tables represented by blue boxes. Lines connecting boxes describe the relationship of the tables to each other.

List of Features	Cutoff for "Clear"	Cutoff for "Opaque"
area	52.75	152
weighted_position_x	1.6	2.2
weighted_position_y	1.2	2.2
median_diameter	1.456	2.11
length	1.686	3.058
form_factor	0.0125	0.0155
perimeter	3.4485	5.569
straight_chord	1.659	2.8585
curved_chord	2.8105	4.372
custom_bend	0.0205	0.024
custom_pinch	0.025	0.0315
custom_wave	0.0225	0.0225
mass	7475.5	26395.5
weighted_moment_of_inertia	1.654	3.49
density_levels	4.445	2.3275
standard_deviation_of_density_levels	3.4245	1.8145
angle	2.059	3.232

Table 2 A measure of system noise per feature. The table shows values for system noise per feature. The system noise values are used to detect changes in motion that can be used to calculate frequency.

Graphical User Interface (GUI) of SchistoView

The GUI of SchistoView (**Figure 7**) allows hierarchical navigation of the experimental results. A Mahalanobis Distance or percent degeneracy heatmap of the assay plate provides a high-level summary. Selecting on a well in the heatmap updates the effect size heatmap which provides insight into which features contribute to the well result. An effect size for a given feature may then be selected to update the histogram of the well, the effect size plot over time, and the effect size dose response plot for that feature. Frequency is visualized with an estimate of the waveform and displays the wavelength and amplitude versus negative control DMSO. An image of the well at the indicated time point is displayed.

The user can toggle the campaigns, iterations within the campaign, data grouping, type of Mahalanobis Distance (or percent degeneracy), effect category, and feature.

SchistoView was created using Excel VBA forms and a MySQL connector to query the database in real-time.

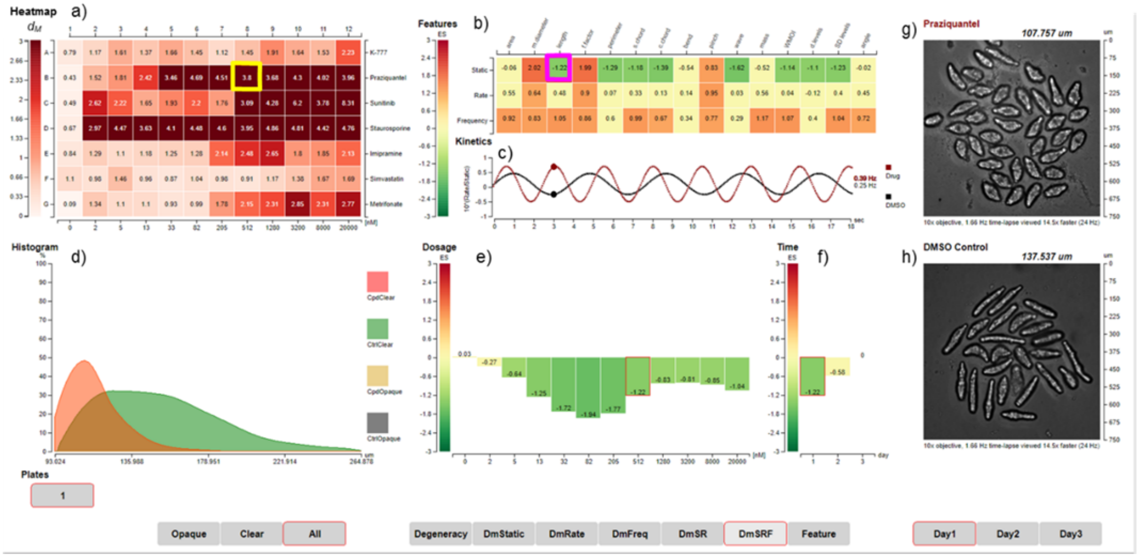

Figure 7. Screenshot of the SchistoView graphical user interface. Selected data are shown to illustrate the hierarchical approach to visualization. **(a)** Heat map of Mahalanobis distances (d_M) for seven test drugs arrayed over an 11-point 2.5-fold dilution series from 2 nM in column 2 to 20 µM in column 12. Drugs, from top to bottom, are, K11777, PZQ, sunitinib, staurosporine, imipramine, simvastatin and metrifonate. DMSO controls are arrayed in column 1 and are shown as the average d_M (0.77) for all DMSO controls. A d_M of 1.61 is significantly different (3 SD) from control. Clicking on coordinate B8 (identified by the yellow square: 512 nM PZQ) populates panels (**b**) and (**g**) (see below). **(b)** Heat map showing the effect sizes (ES) for static, rate and frequency, after exposure to 512 nM PZQ for 2 h, *i.e.*, the selected well from (**a**). Three sets of 15 features are arrayed in rows and columns, respectively. Clicking on the intersection of the length feature and static mode (magenta box) in (**b**) populates panels

(**c**) through (**f**) and the underlying data. (**c**) Calculated waveforms defined by the range of length (amplitude) and frequency of length contraction (frequency). DMSO control worms are slower moving (lower frequency) than those treated with 512 nM PZQ (red line). (**d**) Histogram displaying the distribution of static length for DMSO control worms (green) and PZQ-treated worms (orange). (**e**) Bar graph depicting the ES for static length after PZQ treatment across 11 concentrations (second row in (**a**)). (**f**) Bar graph depicting the ES for static length in the 512 nM PZQ treatment across the three days of measurement. **(g)** First image from time-lapsed movie of the well highlighted in (**a**); in the live SchistoView, the 30-frame movie is looped. (**h**) as for (**g**) except for the DMSO control.
