# Supplementary material for: A multi-dimensional, time-lapse, high content screening platform applied to schistosomiasis drug discovery
Source: Commun Biol. 2020 Dec 21;3:747. doi: 10.1038/s42003-020-01402-5 (PMC7752906; doi:10.1038/s42003-020-01402-5)
Supplement: Supplementary file 2 — Description of Additional Supplementary Files [file 42003_2020_1402_MOESM2_ESM.pdf]

Descriptions of Additional Supplementary File

Supplementary data: Hit and non-hits arising from a screen of a 1,323-member drug set were compared with data from another drug screen that employed an observation-based scoring system (reference 29 in the main text).
