# Supplementary material for: A multi-dimensional, time-lapse, high content screening platform applied to schistosomiasis drug discovery
Source: Commun Biol. 2020 Dec 21;3:747. doi: 10.1038/s42003-020-01402-5 (PMC7752906; doi:10.1038/s42003-020-01402-5)
Supplement: Supplementary file 4 — Reporting Summary [file 42003_2020_1402_MOESM4_ESM.pdf]

nature research

Corresponding author(s): Michelle Arkin (communicating), Conor Caffrey, Rahul Singh

Last updated by author(s): Jul 30, 2020

Reporting Summary

Nature Research wishes to improve the reproducibility of the work that we publish. This form provides structure for consistency and transparency in reporting. For further information on Nature Research policies, see Authors & Referees and the Editorial Policy Checklist.

Statistics

For all statistical analyses, confirm that the following items are present in the figure legend, table legend, main text, or Methods section.

n/a	Confirmed	
☐	☒	The exact sample size (*n*) for each experimental group/condition, given as a discrete number and unit of measurement
☐	☒	A statement on whether measurements were taken from distinct samples or whether the same sample was measured repeatedly
☐	☒	The statistical test(s) used AND whether they are one- or two-sided<br>*Only common tests should be described solely by name; describe more complex techniques in the Methods section.*
☐	☒	A description of all covariates tested
☒	☐	A description of any assumptions or corrections, such as tests of normality and adjustment for multiple comparisons
☐	☒	A full description of the statistical parameters including central tendency (e.g. means) or other basic estimates (e.g. regression coefficient) AND variation (e.g. standard deviation) or associated estimates of uncertainty (e.g. confidence intervals)
☐	☒	For null hypothesis testing, the test statistic (e.g. *F*, *t*, *r*) with confidence intervals, effect sizes, degrees of freedom and *P* value noted<br>*Give P values as exact values whenever suitable.*
☒	☐	For Bayesian analysis, information on the choice of priors and Markov chain Monte Carlo settings
☒	☐	For hierarchical and complex designs, identification of the appropriate level for tests and full reporting of outcomes
☐	☒	Estimates of effect sizes (e.g. Cohen's *d*, Pearson's *r*), indicating how they were calculated

Our web collection on statistics for biologists contains articles on many of the points above.

Software and code

Policy information about availability of computer code

Data collection	Data collection, image analysis, and data analysis are described in detail in a supplementary document entitled 'Supplementary Data 2." Data collection utilized an INCell Analyzer 2000.
Data analysis	Image analysis and data analysis used INCell Developer and custom scripts in Visual Basic for Applications. MySQL, and HTML were used for data storage and visualization, respectively.

For manuscripts utilizing custom algorithms or software that are central to the research but not yet described in published literature, software must be made available to editors/reviewers. We strongly encourage code deposition in a community repository (e.g. GitHub). See the Nature Research guidelines for submitting code & software for further information.

Data

Policy information about availability of data

All manuscripts must include a data availability statement. This statement should provide the following information, where applicable:

- Accession codes, unique identifiers, or web links for publicly available datasets
- A list of figures that have associated raw data
- A description of any restrictions on data availability

Images and associated analyzed data are provided in SchistoView. Multiple places in the text, note that results of the screen are available through Schistoview, accompanied with a link to the website (http://haddock9.sfsu.edu/schistoview/home).

Field-specific reporting

Please select the one below that is the best fit for your research. If you are not sure, read the appropriate sections before making your selection.

☒ Life sciences ☐ Behavioural & social sciences ☐ Ecological, evolutionary & environmental sciences

For a reference copy of the document with all sections, see nature.com/documents/nr-reporting-summary-flat.pdf

Life sciences study design

All studies must disclose on these points even when the disclosure is negative.

Sample size	We utilized 40 somules/well in a round-bottom, 96-well plate. As described in the text, this number provided the maximum density that could reproducibly be segmented.
Data exclusions	No data were excluded.
Replication	The manuscript text described replication. Compounds for the "seven-drug set" were selected based on years of observation and our prior publications, and were chosen to give a broad range of phenotypes to allow us to develop a general methodology for visualization and analysis.
Randomization	Not applicable
Blinding	Not applicable

Reporting for specific materials, systems and methods

We require information from authors about some types of materials, experimental systems and methods used in many studies. Here, indicate whether each material, system or method listed is relevant to your study. If you are not sure if a list item applies to your research, read the appropriate section before selecting a response.

Materials & experimental systems

n/a	Involved in the study	
☒	☐	Antibodies
☒	☐	Eukaryotic cell lines
☒	☐	Palaeontology
☐	☒	Animals and other organisms
☒	☐	Human research participants
☒	☐	Clinical data

Methods

n/a	Involved in the study	
☒	☐	ChIP-seq
☒	☐	Flow cytometry
☒	☐	MRI-based neuroimaging

Animals and other organisms

Policy information about studies involving animals; ARRIVE guidelines recommended for reporting animal research

Laboratory animals	Hamsters (male golden Syrian) and Biomphalaria snails (NMRI) are required to maintain the Schistosoma parasite (NMRI strain).
Wild animals	N/A
Field-collected samples	N/A
Ethics oversight	Vertebrate animal maintenance and use were performed in accordance with UCSF's Institutional Animal Care and Use Committee protocol AN086607-03.

Note that full information on the approval of the study protocol must also be provided in the manuscript.
